# De novo assembly of the complete mitochondrial genomes of two Camellia-oil tree species reveals their multibranch conformation and evolutionary relationships

**DOI:** 10.1038/s41598-025-86411-2

**Published:** 2025-01-23

**Authors:** Zhun Xiao, Yiyang Gu, Junqin Zhou, Mengqi Lu, Jinfeng Wang, Kaizheng Lu, Yanling Zeng, Xiaofeng Tan

**Affiliations:** 1 The Key Laboratory of Cultivation and Protection for Non-Wood Forest Trees, Ministry of Education, Central South University of Forestry and Technology, Changsha, 410004 China; 2 School of Foreign Languages, Changsha Social Work College, Changsha, 410004 China; 3 Key Laboratory of Non-Wood Forest Products of State Forestry Administration, College of Forestry, Central South University of Forestry and Technology, Changsha, 410004 China; 4 Camellia Oil Tree Research Academy, Central South University of Forestry and Technology, Changsha, 410004 China; 5 Hunan Academy of Forestry, Changsha, 410004 China

**Keywords:** *Camellia Oleifera*, *Camellia lanceoleosa*, Mitochondrial genome, De novo assembly, Phylogenetic analysis, Plant biotechnology, Plant evolution, Plant molecular biology

## Abstract

Camellia-oil trees are economically valuable, oil-rich species within the genus *Camellia*, family Theaceae. Among these species, *C. oleifera*, a member of Section *Oleifera* in the genus, is the most extensively cultivated in China. In this study, we assembled the mitochondrial genomes (mitogenomes) of two Camellia species, namely *C. oleifera* and *C. lanceoleosa*. These two species are closely related and belong to the same genus and section, with *C. oleifera* being hexaploid and *C. lanceoleosa* being diploid. The mitogenome of *C. oleifera* is comprised of 1,039,838 base pairs (bp), and *C. lanceoleosa* is comprised of 934,155 bp. Both genomes exhibit a multipartite genome structure, which is supported by our PCR experiments. We conducted codon usage and RNA editing site analysis on these two mitogenomes, which showed highly consistent results. However, analysis of repetitive sequences and mitochondrial plastid sequences (MTPTs) revealed differences between the two mitogenomes. Phylogenetic analysis indicated that these two species clustered together, suggesting a close evolutionary relationship. The collinearity analysis results showed extensive genome rearrangements in the mitogenomes of *Camellia* species. We successfully assembled the mitogenomes of *C. oleifera* and *C. lanceoleosa*, marking a significant advancement in understanding camellia-oil tree mitogenomes. Unlike circular mitogenomes reported before, our research confirms multiple-branched configurations in these two species. This sheds light on mitogenome structural complexities and contributes to our understanding of evolutionary processes. Additionally, these results enrich *Camellia* genetic resources and expand our knowledge of mitogenome variation.

**Supplementary Information:**

The online version contains supplementary material available at 10.1038/s41598-025-86411-2.

## Introduction

Camellia-oil trees generally refer to species within the *Camellia* genus of the Theaceae family that have seeds with high oil content and are of economic cultivation value^1,2^. In China, the primary camellia-oil tree species cultivated include *C. oleifera*, *C. meiocarpa*, *C. chekiangoleosa*, and *C. vietnamensis*. Among these, *C. oleifera*, along with the Mediterranean *Olea europaea* and the Southeast Asian *Elaeis guineensis*, is recognized as one of the top three woody oil-bearing plants globally^3^. Camellia-oil tree seeds have an oil content of 55–60%, and the liquid oil extracted from these seeds is called camellia oil^4^. Camellia oil has a high content of unsaturated fatty acids, up to 90%, including up to 80% oleic acid and about 8% linoleic acid. It is also rich in nutrients and health components such as squalene, phytosterols, and vitamin E. Long-term consumption is beneficial for human health and is recommended by the World Health Organization as one of the highest quality edible plant oils, with more than two thousand years of cultivation history in China^5^. Moreover, camellia oil can serve as a lubricant in the industry, as an ingredient in cosmetics, and has anti-inflammatory and anti-itch medicinal properties^6^. *C. oleifera* is the most extensively cultivated and significant species in China, accounting for over 95% of the national camellia-oil tree cultivation area. As of the end of 2022, China’s *C. oleifera* cultivation area was approximately 467 square kilometers, projected to reach 600 square kilometers by 2025^4^. The *C. oleifera* and the *C. lanceoleosa* tree both belong to Section *Oleifera* of the Genus *Camellia*, with the former being hexaploid (2n = 6x = 90) and the latter diploid (2n = 2x = 30), the only diploid wild species in the Section *Oleifera* of the genus *Camellia*, having the closest genetic relationship with the polyploid *C. oleifera*^7^.

Mitochondria are often referred to as the cell’s powerhouses, essential for energy production through oxidative phosphorylation. Besides energy metabolism, mitochondrial genomes (mitogenome) play vital roles in signaling pathways, stress responses, and programmed cell death^8^. Understanding the structure, organization, and function of plant mitogenomes is crucial for elucidating their contribution to plant biology, evolution, and adaptability. The main features of plant mitogenomes include significant variation in genome size and structure, highly conserved genes, sparse gene distribution, a large amount of non-coding sequences, and extensive RNA editing^9^. The assembly of mitogenomes can present in several different forms. The mitogenome of the majority of plants are presented as a circular DNA. However, the mitogenome of *Lactuca sativa* is reported as linear form^10^. And some plants, such as *Picea sitchensis*^11^, *Thuja sutchuenensis*^12^, and *Abelmoschus esculentus*^13^, their mitogenomes may exhibit a branched structure. For some plants, such as *Scutellaria tsinyunensis*^14^, and *Actinidia chinensis*^15^, the mitogenomes may exhibit different structures through recombination.

In recent years, with the rapid advancement of genomics and molecular biology technologies, there has been increasing research on the genomes of *C. oleifera* and *C. lanceoleosa*, aiming to reveal their genetic backgrounds, improve plant traits, and enhance crop resilience to environmental stresses. Particularly, mitogenome research has been emphasized due to its unique genetic features and critical role in plant growth, development, and adaptability. The published genomes of camellia-oil tree species to date are *C. oleifera* var. “Nanyongensis”, *C. lanceoleos*, and *C. chekiangoleosa*^7,16,17^. Compared to nuclear and chloroplast genome studies, the mitogenome information is still relatively scarce, and only a few mitogenome of *Camellia* species have been published, such as *C. gigantocarpa*^18^. Deepening the study of the mitogenome in the *Camellia* genus enhances our understanding of mitogenome evolution and offers potential insights into identifying key genetic factors influencing important agronomic traits, such as cytoplasmic male sterility. While the current research mainly focuses on genomic characteristics and phylogenetic relationships, similar studies in other plant species have successfully identified mitochondrial genes associated with traits like male sterility^19–21^. Therefore, the data from this study will serve as a valuable resource for future functional genomics research, helping to uncover the genetic mechanisms underlying key agronomic traits in *Camellia*.

In this article, we assembled and annotated the complete mitogenomes of *C. oleifera* and *C. lanceoleosa*. We investigated their mitogenomes’ basic characteristics, structure, sequence migration, and RNA editing. Further analyses on mitochondrial structure and phylogenetics were conducted to ascertain the unique conformations of their mitogenomes and the phylogenetic position of Section *Oleifera* plants. This comparative analysis offers a more comprehensive perspective for understanding the complexity of the mitogenomes in the *Camellia* genus.

## Materials and methods

### Plant materials, DNA extraction, and sequencing

The *C. oleifera* cv. Huashuo was cultivated in an experimental field located in Wangcheng District, Changsha City, Hunan Province (coordinates: 28° 30′ 38” N, 112° 50′ 38” E). The plant material has been preserved in the herbarium of Central South University of Forestry and Technology with the accession number 20200430CS-1, identified by Professor Tan Xiaofeng from Central South University of Forestry and Technology. Similarly, fresh leaves of *C. lanceoleosa* were collected from the Jiangxi Provincial Tree Breeding Center in Yongxiu County, Jiujiang City, Jiangxi Province (coordinates: 28° 57′ 22” N, 115°39′ 55” E). The material has also been deposited in the same herbarium with the accession number 20210918JX-1, identified by Researcher Yang Shixiong from the Kunming Institute of Botany, Chinese Academy of Sciences. Genomic DNA was extracted from leaves of *Camellia* species using DNeasy Plant Mini Kit (QIAGEN). The integrity of the DNA was determined with the Agilent 4200 Bioanalyzer (Agilent Technologies, Palo Alto, California). Eight micrograms of genomic DNA were sheared using g-Tubes (Covaris), and concentrated with AMPure PB magnetic beads.

We constructed Circular Consensus Sequencing (CCS) libraries and performed sequencing on the PacBio Sequel II platform, utilizing four cells. After removing adaptors and discarding low-quality sequences, we conducted quality control using SMRT Link software (version 9.0) and acquired consensus HiFi reads through the ccs tool (https://github.com/PacificBiosciences/ccs). A paired-end library was sequenced on the DNBSEQ T10 platform for additional data.

For the mitogenome assembly of *C. oleifera* and *C. lanceoleosa*, we randomly sampled 10 GB of HiFi data and 10 GB DNBSEQ data from the raw sequencing data, respectively. All DNBSEQ data have a fixed length of 100 bp. In the *C. oleifera* HiFi data, the shortest reads are 1790 bp, the longest reads are 28,869 bp, and the average length is 17,529 bp. For the *C. lanceoleosa* HiFi data, the shortest reads are 1,489 bp, the longest reads are 35,005 bp, and the average length is 14,524 bp. Considering the high copy number of organellar genomes, these data will be sufficient to ensure the accurate and comprehensive assembly of the mitochondrial genomes for both *Camellia* species. The short read data are not involved in assembly, but are used for reads mapping to confirm assembly accuracy.

### Genome assembly and annotation

For the mitogenome assembly of two *Camellia* species, we using Flye assembler^22^ with default parameters to directly assemble the HiFi long-reads and obtain the graphical fragment assembly (GFA) results in GFA format. The obtained GFA includes sequences from the nucleus, mitochondria, and plastids. Due to intracellular sequence migration, contigs from these different sources may be interconnected, forming a complex graph. We adopted a method similar to Fischer’s^23^, identifying the source of each contig based on its coverage depth and sequence similarity to plastid or mitochondrial sequences. First, for all the assembled contigs in FASTA format, we created a BLAST database using makeblastdb and then used the BLASTn program to identify contig fragments containing mitochondrial genes. The query sequences were mitochondrial genes from *C. gigantocarpa* (accession number OP270590.1 in GenBank). The BLASTn parameters were set as “-evalue 1e-5 -outfmt 6 -max_hsps 10-word_size 7”. Additionally, we used the plastid genome of *Camellia oleifera* (NC_023084.1) as the query sequence to identify some plastid-derived contigs, and removed them manually from the GFA. We also removed contigs with low coverage that were likely to originate from the nucleus. The final graph only contained mitochondrial contigs, which had similar coverage depth and included typical mitochondrial genes. Due to our HiFi reads with an average sequencing length close to 15,000 bp and high sequencing accuracy, we did not require additional reassembly or polishing steps. We then visualizing the GFA file using Bandage software (v0.8.1)^24^. Following the assembly, we aligned the short reads to the constructed genome sequences. The outcomes demonstrated that our assembly yielded a contiguous, gapless mitogenome (Figs. S1 and S2).

The protein-coding genes (PCGs) of the mitogenome was annotated using PMGA software^25^. The tRNA genes of the mitogenome was annotated using tRNAscan-SE software (v.2.0.11)^26^. The rRNA genes of the mitochondrial genome were annotated using BLASTN software (v2.13.0)^27^. Any annotation errors in the mitogenome were manually corrected using Apollo software (v1.11.8)^28^.

### Validating the assembled conformation

To confirm the existence of complex conformation in *C. oleifera* and *C. lanceoleosa*, we try to performance PCR experiments to amplify the overlapping regions in GFA. Based on the mitogenome in GFA file of *C. oleifera* and *C. lanceoleosa*, sequences of 2000 bp upstream and downstream of the repetitive region were extracted. Then we using Primer-BLAST (https://www.ncbi.nlm.nih.gov/tools/primer-blast)^29^ to design primers. A total of 16 and 8 pairs of primers were designed for *C. oleifera* and *C. lanceoleosa*, respectively. Detail information can be found in additional file Table S1.1 and S1.2. The primers were synthesized by Sangon Biotech (Shanghai) Co., Ltd. The PCR reactions were carried out in a 50 µl mixture, including 25 µl 2×Phanta Max Master Mix, 2 µl total DNA, 2 µl forward and reverse primers, and 19 µl ddH2O. After an initial denaturation at 95 °C for 3 min, 35 cycles of PCR were performed. Each cycle consisted of denaturation at 95 °C for 15 s, annealing at 55 °C for 15 s, and extension at 72 °C for 15 s. The PCR products were sequenced using Sanger sequencing.

### Codon usage

The extraction of protein-coding sequences from the genome was performed using PhyloSuite software (v1.1.16)^30^. Subsequently, Mega software (v7.0)^31^ was employed to conduct bias analysis of codon usage on the mitochondrial PCGs, we calculate the RSCU (Relative Synonymous Codon Usage) values.

### Repeat elements analysis

The identification of repetitive sequences, including simple sequence repeats (SSRs), tandem repeats, and dispersed repeats, was performed using MISA (v2.1)(https://webblast.ipk-gatersleben.de/misa/)^32^, TRF (v4.09) (https://tandem.bu.edu/trf/trf.unix.help.html)^33^, and the REPuter web server (https://bibiserv.cebitec.uni-bielefeld.de/reputer/)^34^. The results were visualized using Excel software (2021) and the Circos package (v0.69.9)^35^.

### Identification of MTPTs

To compare whether there is sequence migration between chloroplast and mitochondrial genomes. We employed GetOrganelle software^36^ to assemble the chloroplast genome sequence with the parameter ‘-F embplant_pt -R 15 -k 27,47,63,77,89,99,105′. We using CPGAVAS2^37^ to annotate the chloroplast genomes of two *Camellia* species. The chloroplast genome of *C. oleifera* was used as reference, with the accession number NC_023084.1 in GenBank. The annotation results of the chloroplast genome were corrected with CPGView^38^. Homologous fragments were analyzed using BLASTN software (v2.13.0) with specific parameters: -evalue 1*e*-5, -gapopen 5, - gapextend 2, -reward 2, -penalty − 3, and the results were visualized using the Circos package (v0.69.9)^35^.

### RNA editing event analysis

We extracted the sequences of all PCGs encoded by the mitogenome of *C. oleifera* and *C. lanceoleosa*, and then using Deepred-mt^39^ to predict the C-to-U RNA editing sites. Deepred-mt analyzes all the C base in the sequence and gives a prediction of whether they have been edited based on the built-in trained model. To improve accuracy, we reserved only predictions with probability values above 0.9 as C-to-U edited sites.

### Phylogenetic analysis

Based on phylogenetic relationships, we select closely related species and download their mitogenomes. Including our two newly sequences mitogenomes, a total of 39 mitogenomes were used for phylogenetic analysis. The specific accession number of these mitogenome can be found in Table S2. Among them, two mitogenomes from the order Lamiales were used as outgroups: *Sedum plumbizincicola* (NC_069572.1) and *Rhodiola tangutica* (OP573219.1). Subsequently, we utilized PhyloSuite software (v1.1.16)^30^ to extract the 24 conserved shared genes (refer to the legend of the phylogenetic tree for detail gene list). Next, we conduct multiple sequence alignment using MAFFT software (v7.505)^40^, then all aligned shared gene sequences were concatenated. We perform phylogenetic analysis using method of maximum likelihood by employing IQ-TREE software (v1.6.12)^41^, the parameters are: --alrt 1000 -B 1000. The best-fit model was GTR + F + R2. Finally, we visualize the results of the phylogenetic tree using ITOL software (v6)^42^.

### Synteny analysis

The BLAST program was used to obtain pairwise BLASTN results for comparing each mitogenome. Homologous sequences with a length exceeding 500 bp were retained as conserved collinear blocks for constructing the Multiple Synteny Plot. The parameters of BLASTN are: “-word_size 7 -evalue 1*e*-10 -outfmt 6 -num_threads 10”.

## Results

### Mitogenomic structure

**Fig. 1:**
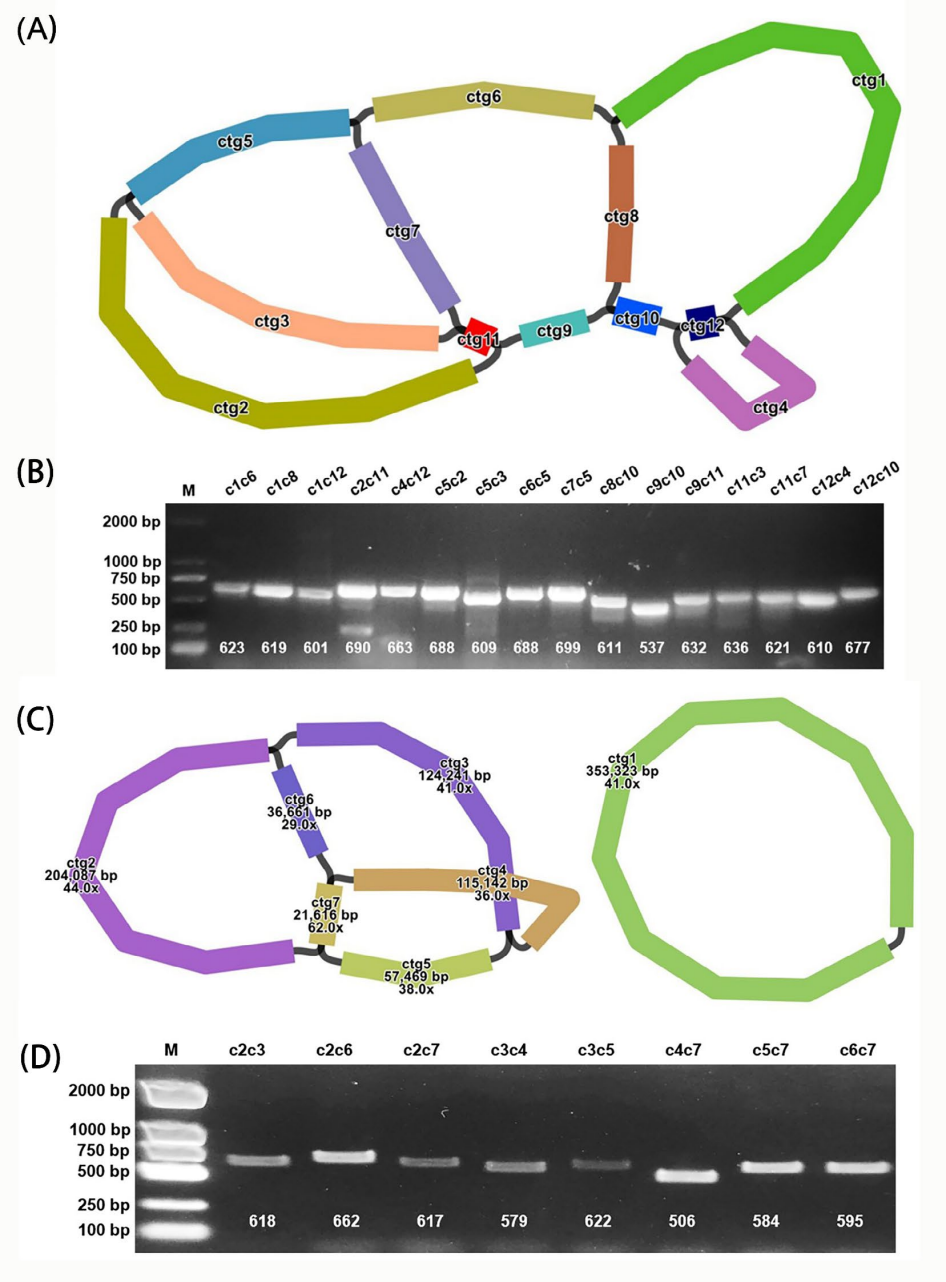
Diagram of mitogenome assembly of *C. oleifera* and *C. lanceoleosa*. (**A**) Graphical fragment assembly (GFA) results of *C. oleifera* based on Flye assembler. (**B**) Validation of each linkage in the *(C) oleifera* GFA. (**C**) Graphical fragment assembly (GFA) results of *C. lanceoleosa* based on Flye assembler. (**D**) Validation of each linkage in the *C. lanceoleosa* GFA. Each sequence is represented by a node in the GFA, and detailed information for each node can be found in Table 1. Linkages between nodes represent the existence of overlapping regions. Verified regions are indicated above the sample wells of gel electrophoresis plot. For example, ‘c1c6’ represents PCR validation of the linkage between ctg1 and ctg6. ‘M’ stands for marker. Expected lengths (bp) of each PCR product are indicated below the electrophoresis plot. The full uncropped Gels image can be seen in Figs. S3 and S5. Our results confirm the presence of these linkages, indicating the diversity of genome structures. For *C. oleifera*, we randomly selected the path ctg8-ctg10-ctg12-ctg4-ctg12-ctg1-ctg6-ctg5-ctg2-ctg11-ctg7-ctg5-ctg3-ctg11-ctg9 to represent its complete mitogenome sequence. For *C. lanceoleosa*, we randomly used ctg1 and ctg5-ctg7-ctg4-ctg3-ctg2-ctg7-ctg6 to represent its complete mitogenome sequence.

We utilized Bandage software^24^ to visualize the assembly graph of the mitochondrial genome obtained from long-read data. The final graphs are presented in Fig. 1. The graph for the mitogenome of *C. oleifera* comprises 12 nodes (Fig. 1A), with each node representing an assembled contig and denoted by a specific label (start with ctg). The linkage between nodes, representing overlap regions between the adjacent nodes. The assembly graph for *C. lanceoleosa* mitogenome includes 7 nodes (Fig. 1C). Details regarding the length and sequencing depth for each node of both species are enumerated in Table 1.

**Table 1:** Nodes Information of *C. oleifera* and *C. Lanceoleosa* in GFA.

Species	Contigs	Length (bp)	Depth
*C. oleifera*	ctg1	179,675	54×
*C. oleifera*	ctg2	227,538	54×
*C. oleifera*	ctg3	134,021	50×
*C. oleifera*	ctg4	90,003	47×
*C. oleifera*	ctg5	84,318	64×
*C. oleifera*	ctg6	74,468	48×
*C. oleifera*	ctg7	65,145	48×
*C. oleifera*	ctg8	45,596	46×
*C. oleifera*	ctg9	20,758	36×
*C. oleifera*	ctg10	14,358	83×
*C. oleifera*	ctg11	5697	91×
*C. oleifera*	ctg12	4123	111×
*C. lanceoleosa*	ctg1	353,323	41×
*C. lanceoleosa*	ctg2	204,087	44×
*C. lanceoleosa*	ctg3	124,241	41×
*C. lanceoleosa*	ctg4	115,142	36×
*C. lanceoleosa*	ctg5	57,469	38×
*C. lanceoleosa*	ctg6	36,661	29×
*C. lanceoleosa*	ctg7	21,616	62×

For each linkage of the GFA, we conducted PCR experiments to confirm their validity. The results corroborating the linkages in the *C. oleifera* mitogenome are depicted in Fig. 1B (The full uncropped Gels image can be seen in Fig. S3). The PCR amplification of the 16 linkage generated bands corresponding to the expected sizes, and subsequent Sanger sequencing confirmed the accuracy of these amplified products by compare to the template sequences(Graphical results of the key linkage positions from Sanger sequencing are shown in Fig. S4). This establishes the veracity and dependability of the contig linkages in the mitogenome of *C. oleifera*.

Similarly, we verified the linkages in the mitogenome of *C. lanceoleosa*, with results highlighted in Fig. 1D (The full uncropped Gels image can be seen in Fig. S5). PCR amplification successfully generated bands of the expected sizes at all 8 linkages. The consensus of Sanger sequencing results with the template sequences further authenticates the contig linkages, confirming them as both actual and trustworthy (Graphical results of the key linkage positions from Sanger sequencing are shown in Fig. S6).

### Genome content

The mitogenome of *C. oleifera* spanning 1,039,838 bp with a GC content of 45.71%. Upon annotation, we identified 38 unique PCGs within the *C. oleifera* mitogenome. Additionally, there are 22 tRNA genes (featuring 8 duplicates), alongside 3 rRNA genes (comprising 2 duplicates). The PCGs encompass various subcategories: 5 ATP synthase genes (*atp1*, *atp4*, *atp6*, *atp8*, *atp9*), 9 NADH dehydrogenase genes (*nad1*, *nad2*, *nad3*, *nad4*, *nad4L*, *nad5*, *nad6*, *nad7* and *nad9*), 4 cytochrome *c* biogenesis genes (*ccmB*, *ccmC*, *ccmFC*, *ccmFN*), 3 cytochrome c oxidase genes (*cox1*, *cox2* and *cox3*), and membrane transport protein gene (*mttB*), maturation enzyme gene (*matR*), and cytochrome *b* oxidase genes (*cob*), 4 ribosomal large subunit genes (*rpl2*, *rpl5*, *rpl10*, *rpl16*), 8 ribosomal small subunit genes (*rps1*, *rps3*, *rps4*, *rps7*, *rps12*, *rps13*, *rps14*, *rps19*), and 2 succinate dehydrogenase genes (*sdh3* and *sdh4*). It is worth noting that four PCGs have two copies, they are *cox1*, *rpl16*, *rps3* and *rpl2*.

**Fig. 2:**
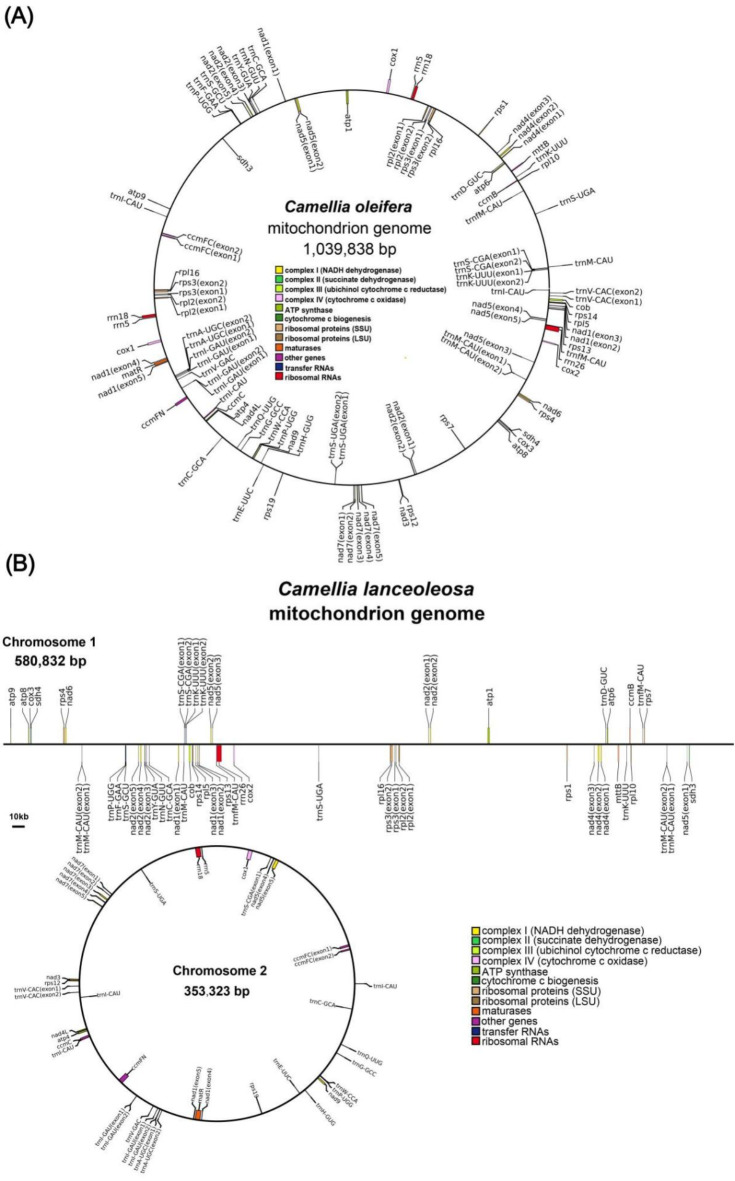
Mitochondrial genome maps. (**A**) Mitogenome map for *C. oleifera*; (**B**) Mitogenome map for *(C) lanceoleosa*. Using blocks of different colors to represent mitochondria genes with different functions.

For *C. lanceoleosa* mitogenome, the total of the two parts is 934,155 bp with a GC content of 45.70%. There is a total of 38 unique PCGs, 22 tRNA genes (inclusive of 9 duplicates), and 3 rRNA genes were identified in *C. lanceoleosa*. Compared to *C. oleifera*, All PCGs are single-copy. Figure 2A and B respectively depict the mitogenome maps of these two species.

### Mitochondrial genome codon usage bias analysis

**Fig. 3:**
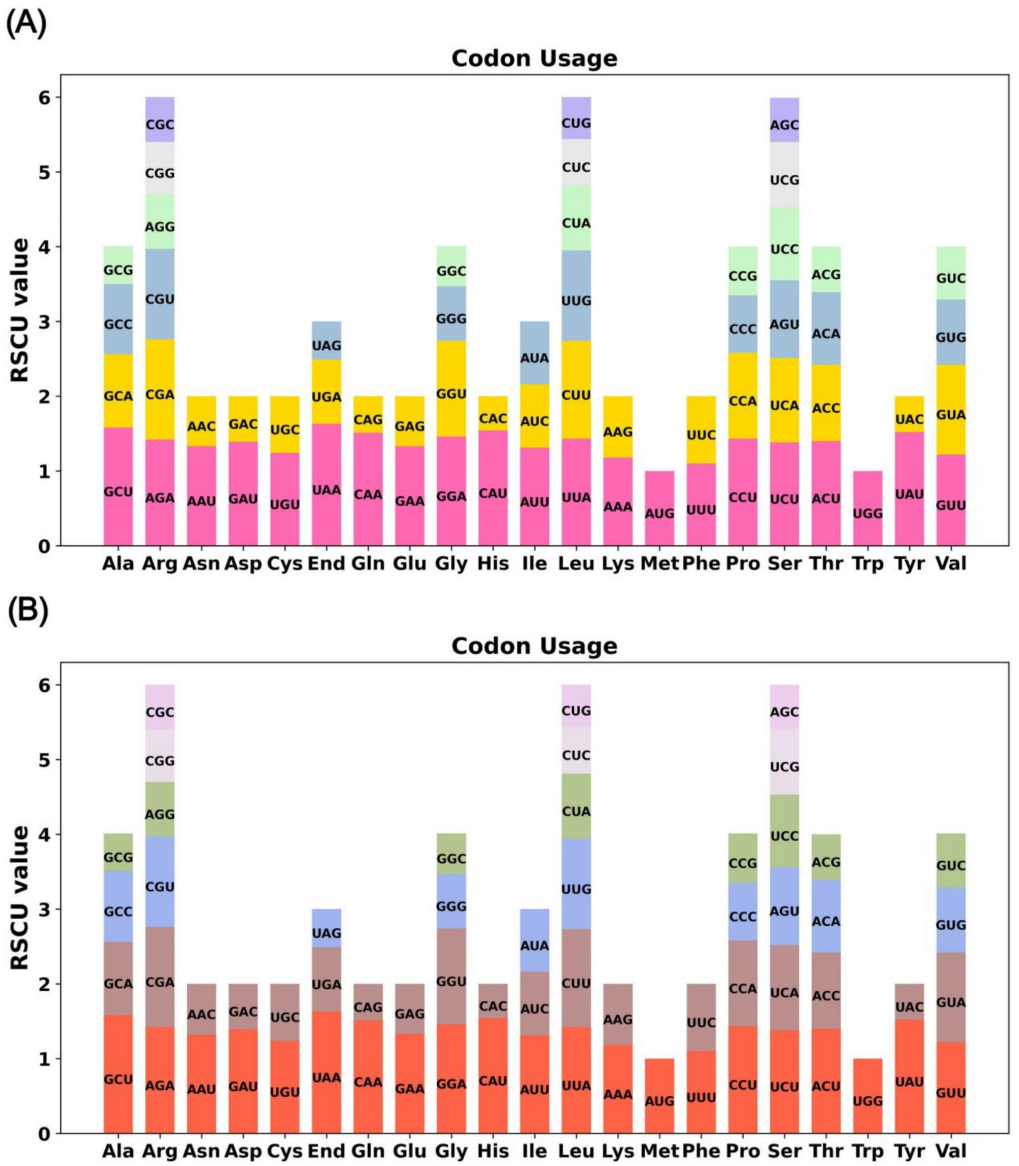
Codon usage of mitochondrial protein-coding genes. (**A**) Codon usage bias analysis results for *C. oleifera*. (**B**) Codon usage bias analysis results for *(C) lanceoleosa*.

We performed an analysis of codon usage bias on the 38 unique PCGs of *C. oleifera*. The frequencies of each codon usage for every amino acid are provided in Table S3.1. The codons exhibiting a RSCU value greater than 1 indicate a preference in usage. Figure 3A reveals a widespread bias in mitochondrial PCGs, except for the start codon AUG and the tryptophan codon UGG. Notably, the UAA stop codon exhibits a pronounced preference, with an RSCU of 1.63, the highest among the mitochondrial PCGs. Then closely followed by the alanine (Ala) codon GCU with an RSCU of 1.58. However, the maximum RSCU values for lysine (Lys) and phenylalanine (Phe), being below 1.2, suggest a minimal codon usage bias for these amino acids. A similar analysis was conducted on the 38 unique PCGs of the *C. lanceoleosa* mitochondrial genome. Each codon’s usage for their respective amino acids can be found in Table S3.2. *C. lanceoleosa* shows a similar codon usage preference to species *C. oleifera* (Fig. 3B).

### Repetitive elements

**Fig. 4:**
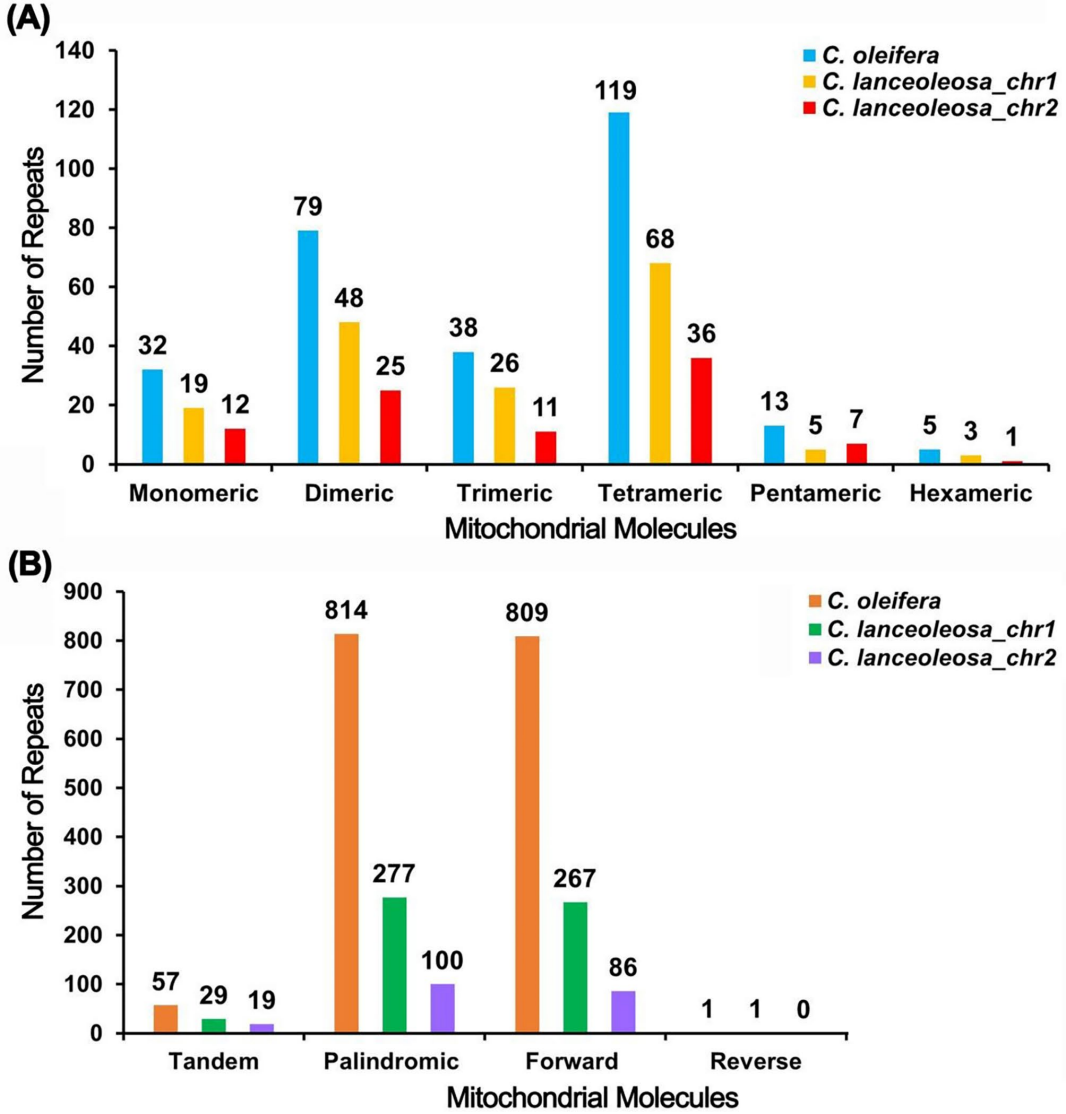
Bar chart of repetitive sequence in the two mitogenomes. (**A**) The X-axis represents the types of SSRs, while the Y-axis represents its number. The blue color of bar represents species *C. oleifera*, the yellow and red color represents the two parts of mitogenome of species *C. lanceoleosa*. (**B**) The X-axis represents the types of tandem repeats and three type of dispersed repetitive sequences, while the Y-axis represents the number of repetitive fragments. The orange color represents species *(C) oleifera*, the green and purple color represents the two parts of mitogenome of species *C. lanceoleosa*.

The mitogenome of *C. oleifera* was analyzed, revealing 286 SSRs (Table S4.1, Fig. 4A), with monomers and dimers making up 38.81% of the total SSRs. Of these, adenine (A) repeats constituted 56.25% of the monomeric SSRs (18 out of 32). Tandem repeat sequences, consisting of core repeat units approximately 7 to 200 base pairs long, were found to be common features in mitogenome. In *C. oleifera* mitogenome, 57 tandem repeats with over 70% sequence similarity and lengths ranging from 12 to 70 base pairs were identified (Table S4.2, Fig. 4A). Additionally, the genome contained 1624 dispersed repetitive sequences of 30 bp or longer, comprising 814 palindromic repeats, 809 forward repeats, and a single reverse repeat. No complementary repeats were detected. The longest palindromic and forward repeats measured 5697 and 84,318 base pairs, respectively. This extensive repetitive fragment has resulted in some PCGs obtaining multiple copies (Table S4.3).

In the analysis of *C. lanceoleosa* mitogenome, chromosome 1 including 169 SSRs were found, with 39.64% being monomeric and dimeric SSRs (Table S4.1, Fig. 4B). Thymine (T) repeats accounted for 57.89% of the monomeric SSRs (11 out of 19). We identified 29 tandem repeats with a similarity exceeding 76% and lengths from 8 to 39 base pairs (Table S4.2). This chromosome also featured 545 pairs of dispersed repeat sequences, each at least 30 base pairs in size, including 277 palindromic and 267 forward repeats, with one reverse repeat (Table S4.3). The longest palindromic and forward repeats were 791 and 21,616 base pairs, respectively.

Furthermore, chromosome 2 exhibited a total of 92 SSRs, with monomeric and dimeric types accounting for 40.22%. Adenine (A) and thymine (T) repeats made up 83.33% of monomeric SSRs (10 out of 12). We found 19 tandem repeat sequences that had a similarity of more than 79% and were between 14 and 61 base pairs in length. Analysis revealed 186 dispersed repeat sequences of at least 30 base pairs, including 100 palindromic and 86 forward repeats. There were no reverse or complementary repeats present. The longest palindromic repeat was 89 base pairs, and the longest forward repeat measured 144 base pairs.

Overall, *C. oleifera* has more and longer repetitive sequences compared to *C. lanceoleosa*, which may account for the difference in genome sizes between the two.

### Identification of mitochondrial plastid DNAs (MTPTs)

**Fig. 5:**
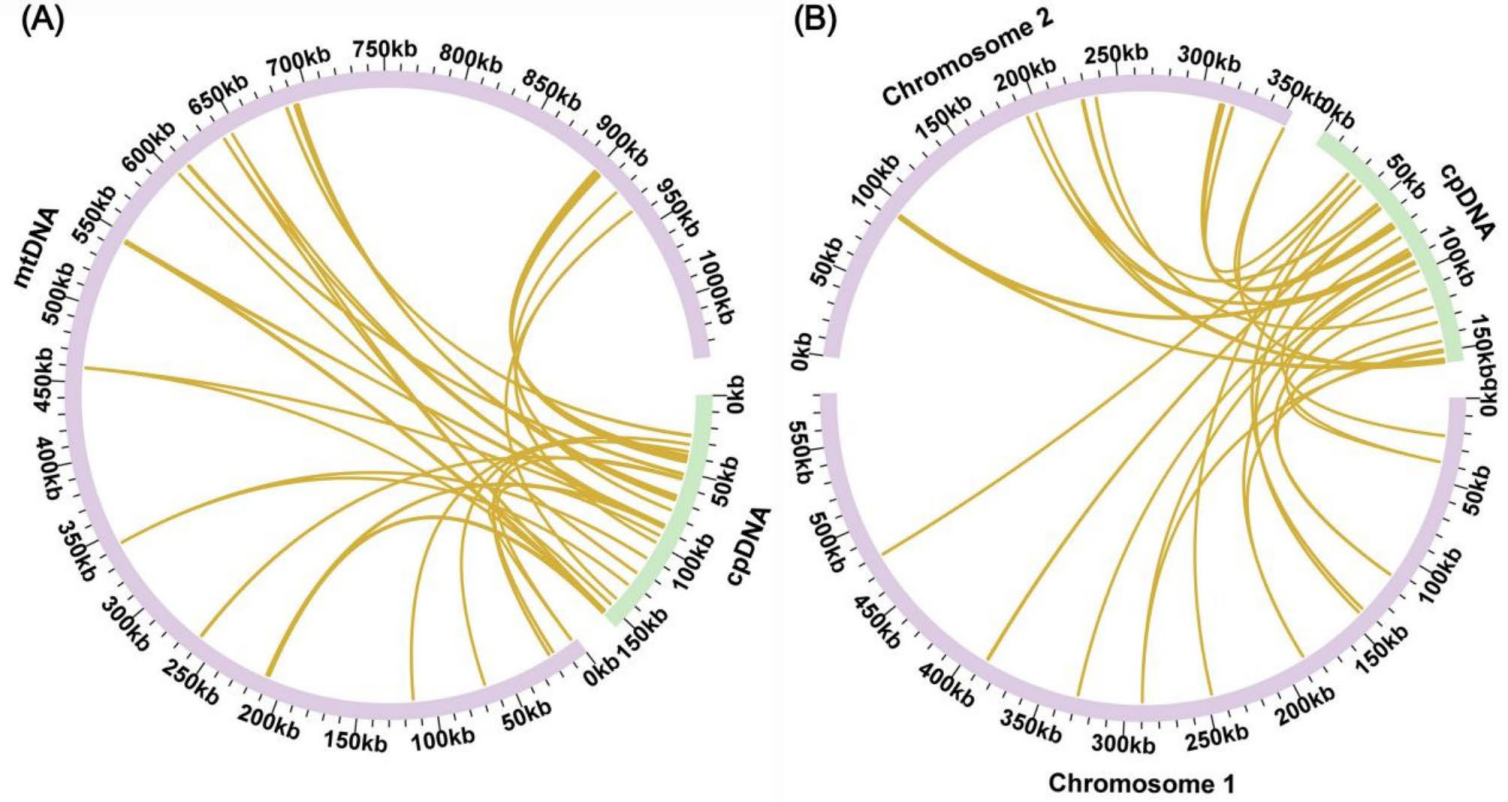
Sequence migration analysis. (**A**) Chord diagram of homologous sequences of *C. oleifera*. (**B**) Chord diagram of homologous sequences of *(C) lanceoleosa*. In the figure, purple arcs represent mitochondrial genomes, green arcs represent chloroplast genomes, and yellow lines between arcs correspond to homologous genomic segments.

We used a chord diagram to illustrate the homologous sequences between the two organelle genomes of two *Camellia* species, where Fig. 5A represents *C. oleifera* and Fig. 5B represents *C. lanceoleosa*. In *C. oleifera*, our analysis reveals there are 20 homologous segments between the mitochondrial and chloroplast genomes, totaling 12,382 bp (Table S5.1). This accounts for 1.19% of the mitogenome. The longest of these segments, MTPT4, is 4311 bp in length. The annotation of these segments has identified 14 complete genes, comprised of 8 PCGs (*rpl2*,* petG*,* petL*,* psbJ*,* psbL*,* psbF*,* psbE*, and *ndhJ*) along with 6 tRNA genes (*trnD-GUC*,* trnI-CAU*,* trnN-GUU*,* trnM-CAU*,* trnP-UGG*,* trnW-CCA*).

Similarly, in *C. lanceoleosa*, 20 homologous segments are found between the mitochondrial and chloroplast genomes, spanning a total of 6,604 bp, which is 0.71% of the mitochondrial genome’s length (Table S5.2). Here, the longest segment is MTPT2, measuring 1689 bp. Annotations of these segments show an equivalent set of 14 complete genes, including the same 8 PCGs and 6 tRNA genes as found in *C. oleifera*.

### RNA editing event

In the mitochondria of *C. oleifera*, RNA editing was explored in 38 unique PCGs, utilizing a cutoff value of 0.9 for identification. This analysis revealed 576 potential RNA editing sites, all reflecting cytosine to uracil (C to U) transitions, within these PCGs (Fig. S7). Among these, the *ccmB* gene stood out with 39 editing sites, marking it as the most extensively edited mitochondrial gene. Following closely were the *ccmC* and *mttB* genes, each with 35 RNA editing events. Due to the complete identity of the coding regions between *C. oleifera* and *C. lanceoleosa*, we obtained consistent results in the analysis of RNA editing sites for the two species.

### Phylogenetic analysis

**Fig. 6:**
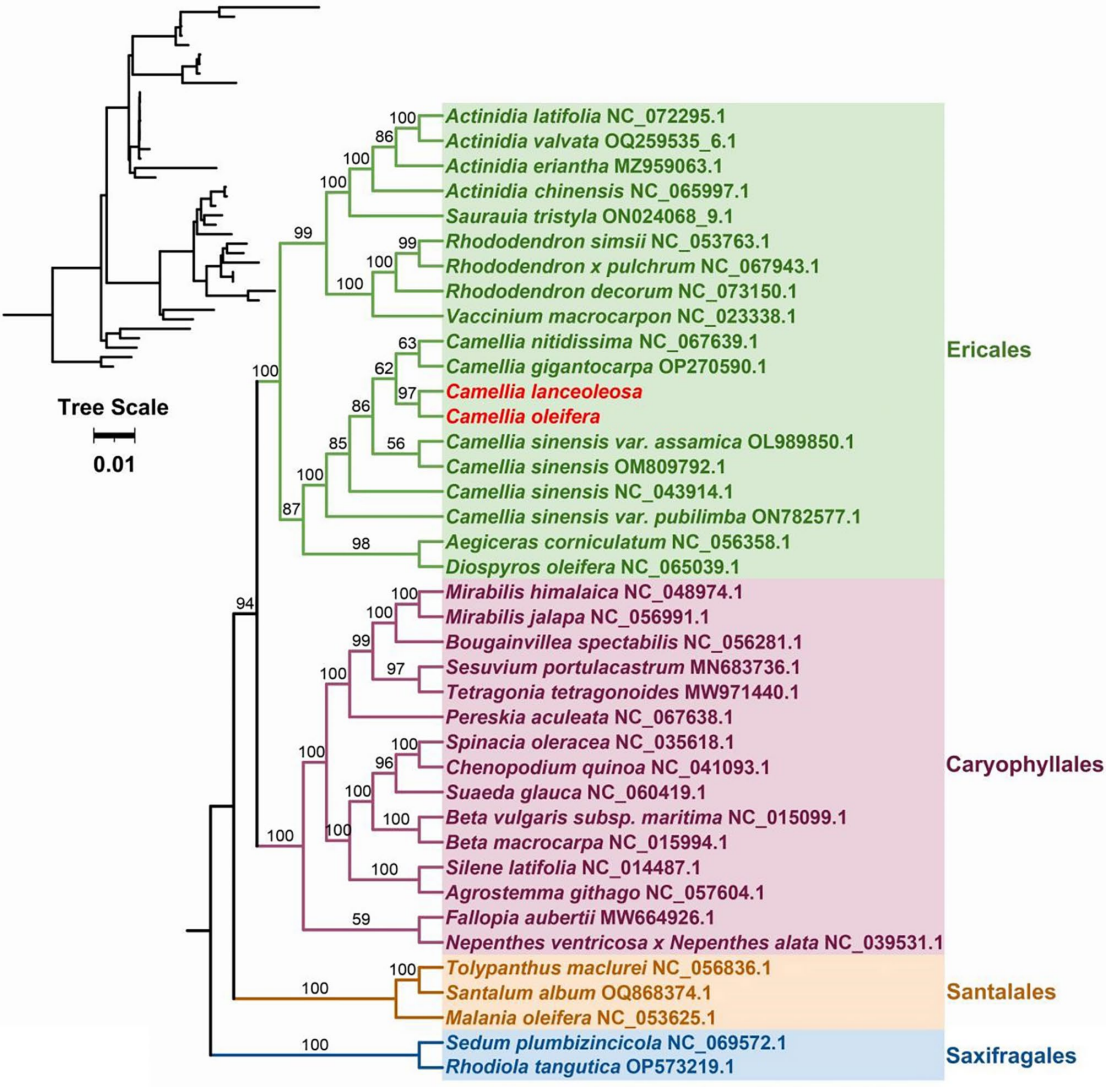
Phylogenetic tree based on shared 24 mitochondrial genes. Two species, *Sedum plumbizincicola* (NC_069572.1) and *Rhodiola tangutica* (OP573219.1) were used as outgroups. The accession numbers of the remaining downloaded mitogenome from GenBank are marked to the right of their Latin names. We utilized 24 shared genes to construct the data matrix, including: *atp1*, *atp4*, *atp6*, *atp8*, *atp9*, *nad1*, *nad2*, *nad3*, *nad4*, *nad4L*, *nad5*, *nad6*, *nad7*, *nad9*, *ccmB*, *ccmC*, *ccmFC*, *ccmFN*, *cox1*, *cox2*, *cox3*, *mttB*, *cob* and *matR*. In the top left corner is a thumbnail of the phylogenetic tree with branch lengths preserved, and the scale of the tree is labeled below. The two mitogenomes we sequenced in this study are highlighted in red font.

The topology of the phylogenetic tree based on mitochondrial DNA is consistent with the latest classification by the Angiosperm Phylogeny Group (APG). Species *C. oleifera* and *C. lanceoleosa* both belong to the family Theaceae within the order Ericales and are clustered together in the phylogenetic tree (Fig. 6). Our results indicate that the two *Camellia* species, *C. oleifera* and *C. lanceoleosa*, are closely related in terms of phylogenetics based on mitochondrial DNAs, compared to other *Camellia* species.

### Collinearity of mitogenomes of *Camellia* species

**Fig. 7:**
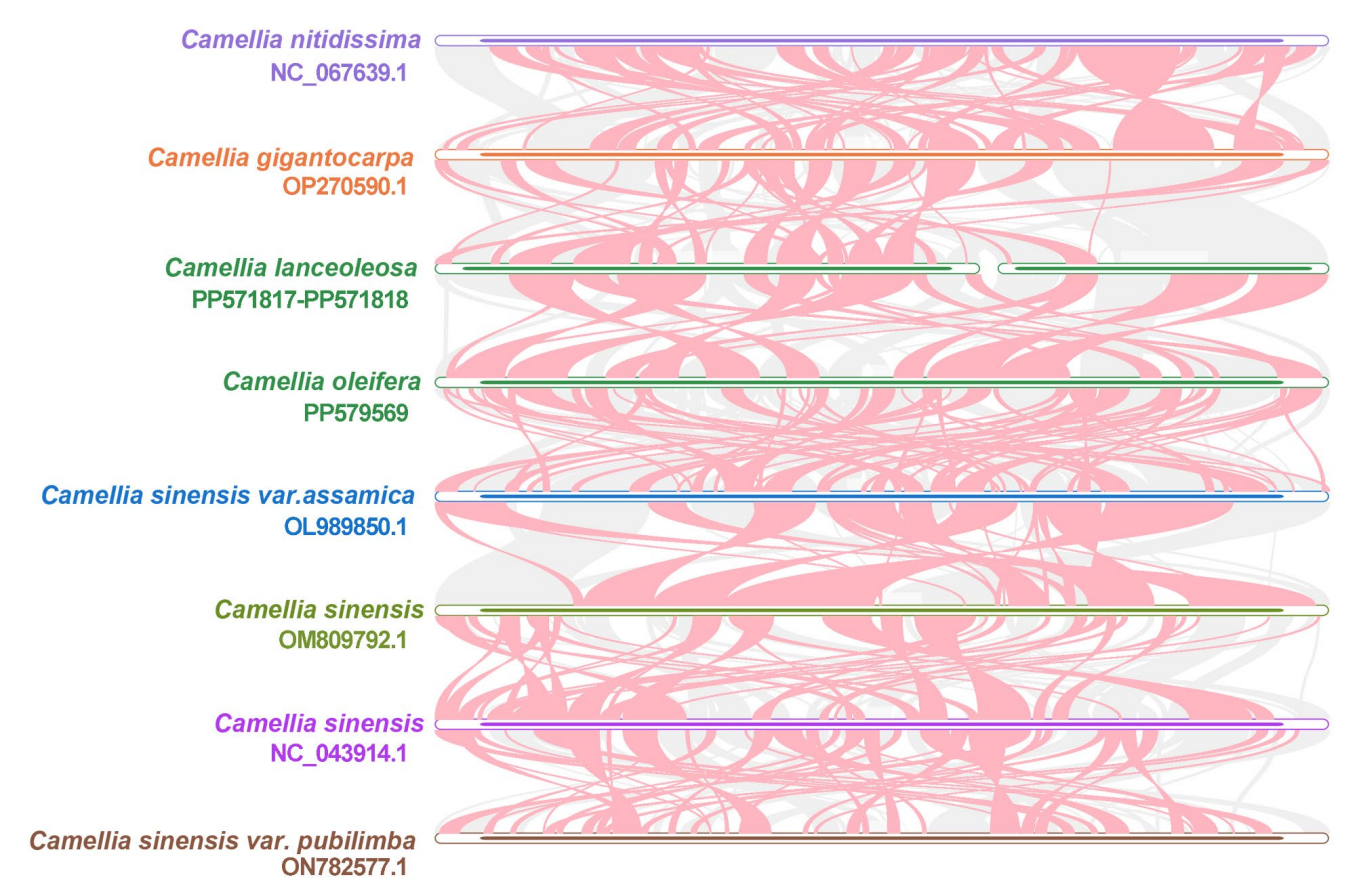
Collinearity comparison of mitogenomes among species of the *Camellia* genus. The red arc regions denote inverted regions, while the gray areas highlight homologues regions without inverted. To enhance clarity, collinear blocks shorter than 0.5 kilobases (kb) were excluded from the analysis. Additionally, certain areas appeared blank, signifying sequences unique to the two *Camellia* species, with no homology to sequences in other species.

Based on sequence similarity, we utilized the source code of the MCscanX software^43^ to generate Multiple Synteny Plots for two *Camellia* species and their closely related species. The results are as follows:

Figure 7 illustrates the homologous regions of mitogenomes between different *Camellia* species. This approach revealed numerous homologous collinear blocks between the two *Camellia* species and other closely related species within the genus. Additionally, certain areas appeared blank, signifying sequences unique to the two *Camellia* species, with no homology to sequences in other species.

These observations indicate a varied arrangement of collinear blocks among the mitochondrial genomes of different *Camellia* species, suggesting that these genomes have experienced significant rearrangements when compared to those of closely related species. Furthermore, the mitochondrial genome sequence arrangements in eight *Camellia* species demonstrate a high degree of variability, pointing to frequent genomic recombination events. This variability underscores the complex nature of mitochondrial genome evolution within the *Camellia* genus, highlighting the importance of further research to understand these genomic changes.

## Discussion

### Mitochondrial genome size variation

Mitochondria are essential to cellular energy conversion, providing the energy necessary for plant life. Plant mitogenomes are notably more complex than those of animals because they are larger and contain more repetitive sequences^10^. This article reports the mitogenomes of *C. oleifera* and *C. lanceoleosa*, two important oil crops in the *Camellia* genus. *C. oleifera*, primarily a hexaploid, stands as an important oil crop. In contrast, *C. lanceoleosa*, a diploid, is its close wild relative. Researching their mitochondrial genomes sheds light on their evolutionary connections.

We particularly focus on the structural characteristics of these two plants’ mitogenomes. The mitogenome of *C. oleifera* is 1,039,838 bp in length, with a GC content of 45.71%. However, *C. lanceoleosa* mitogenome is divided into two parts, totaling 934,155 bp, with a GC content of 45.7%. The GC content in both species is consistent with other plants. For instance, *Ipomoea batatas* at 44.08%^45^, *Actinidia chinensis* at 45.87%^46^, *Prunus salicina* at 45.43%^47^, and *Panax notoginseng* at 45.09%^48^, showing the conservation of GC content in angiosperms. Among *Camellia* species, *C. oleifera* mitogenome is second in size only to that of *C. sinensis*^47^. The differences in mitogenome size are more pronounced among species of the *Camellia* genus. Sizes of published *Camellia* mitogenomes had significant disparity, ranging from 707,441 bp to 1,081,966 bp. The minimal is *C. assamica* mitogenome, with only 707,441 bp^48^. One possibility is that the assembly of the mitogenome of *C. assamica* is incomplete, and there might be an independent portion overlooked, like what was reported for our *C. lanceoleosa*. This is because their report lacks core mitochondrial genes, such as *cox1*, *cox3*, *atp8*, *nad7*, and *nad9*. Excluding this case, other species usually have genomes approximately between 0.9 and 1 mb. That is, there is a fluctuation of approximately 10–15 kb. Similarity in genome size and structure is evident among related species, such as *Toona sinensis* and *Toona ciliata*^49^, as well as different *Broussonetia* species^50^. Repetitive sequences may account for this difference in *Camellia*, as there is a noticeable distinction in the number of dispersed repeats (1624 vs. 731) between the two species we sequenced, particularly with the largest repetitive sequences being different in *C. oleifera* (84,318 bp) and *C. lanceoleosa* (21,616 bp). This difference also results in certain Protein-Coding Genes (PCGs) forming multiple copies in species *C. oleifera*.

Different types of repetitive sequences, including tandem repeats, SSRs, and dispersed repeats, are pivotal components of the mitogenome^51,52^. These sequences are critical for intermolecular recombination, significantly influencing mitochondrial genome assembly^53^. Notably, numerous repeat sequences in the mitogenomes of *C. oleifera* and *C. lanceoleosa* suggest frequent intermolecular recombination. As our PCR experiments have revealed, there may be multiple distinct genome structures coexisting within the cell. Such activity implies dynamic alterations in the mitogenome structures through recombination processes. We have also compared these genomes with those of other terrestrial plants to discern their organizational patterns.

### Multi-branch structure in plant mitogenomes

Plant mitogenomes exhibit diverse conformation, a phenomenon largely attributed to mitogenome recombination facilitated by repetitive sequences and subsequent DNA rearrangements^54^. Such recombination involves the reshuffling of repeat sequences across identical or different chromosomes, playing an integral role in defining mitogenome architecture.

Mitogenome rearrangement occurs at a notable frequency, a trend linked to the specialized DNA repair mechanisms inherent to these organelles. Reports indicate that in the coding regions of plant mitogenomes, repair is primarily conducted through base excision repair and gene conversion^14^. These methods ensure meticulous repair fidelity within mitochondria. Conversely, non-coding regions are repaired through less precise methods, such as non-homologous end joining or break-induced replication. Often, these non-precise repairs encompass the integration of exogenous DNA sequences deriving from nuclear or plastid genomes. This integration encourages the broadening of repeat sequences in non-coding regions and consequently, increases the rate of recombination events.

These repeat sequence-mediated chromosomal recombinations are significant evolutionary drivers, contributing to the dynamic evolution of mitogenomes. When examining the assembly drafts of *C. oleifera* and *C. lanceoleosa*, a distinctive multi-branch conformation is observed, a structural complexity not previously detected in the mitogenomes of other *Camellia* species. Because previously reported ones are all circular mitogenome. This study not only highlights these unique configurations but also provides experimental verification of their presence within the mitogenomes of these two camellia-oil tree species. While the structural differences between camellia-oil trees and other *Camellia* species can be explained by genome rearrangement, it remains unclear what impact these structural changes may have on *Camellia*.

### Homologous sequences in the mitochondrial and chloroplast genomes

The presence of homologous sequences across mitochondrial, chloroplast, and nuclear genomes can be ascribed to sequence transfer events that have shaped their evolution. Mitochondria and chloroplasts, despite their distinct functions and disparate evolutionary origins, harbor a collection of shared genes. These shared genes serve as molecular vestiges of their shared ancient ancestry and their endosymbiotic inception^55^.

A comparative analysis of the chloroplast and mitochondrial genomes in *C. oleifera* and *C. lanceoleosa* reveals the presence of 20 homologous segments within both species, suggesting horizontal sequence transfer might occur between these cellular organelles. Among the genomic crossovers in these *Camellia* species are 9 tRNA genes, which have notably transferred from the chloroplast to the mitochondrial genome. Such transfers of tRNA genes from chloroplasts to mitochondria are not uncommon; they are, in fact, a frequent occurrence within the phylum of angiosperms^56^. We compared the MTPTs of the two camellia-oil tree species and found both 13 similarities and 6 differences. The shared MTPTs might have undergone sequence transfer before species differentiation, whereas the distinct MTPTs could have transferred after species divergence. We speculate that this sequence transfer is ongoing and persistent. Consequently, chloroplast genomes also provide the original sequence material for mitogenome variation, accelerating the process of mitogenome variation.

## Conclusion

We have successfully assembled the complete mitogenomes of *C. oleifera* and *C. lanceoleosa*, which represents a significant advancement in our understanding of the mitogenome of camellia-oil trees. In contrast to previously reported circular mitogenomes in genus *Camellia*, our research has verified the existence of multiple-branched configurations in the two camellia-oil trees. Our findings bring to light the complexities of mitogenome structure in these species and contribute valuable knowledge towards the comprehension of evolutionary processes. Furthermore, these results enrich the genetic resources of the genus *Camellia*, and expanding our understanding of mitogenomes variation.

## Electronic supplementary material

Below is the link to the electronic supplementary material.

Supplementary Material 1

Supplementary Material 2

## Data Availability

The mitogenome sequence is available in nucleotide database of NCBI with accession numbers: PP579569, PP571817 and PP571818. The sequencing reads used for mitogenome assembly in this study have been released on the NCBI with those accession numbers: PRJNA1099614(BioProject); SAMN40944252 and SAMN40944253 (BioSample); SRR28672258, SRR28672259, SRR28674428 and SRR28674429 (SRA).
