# Supplementary material for: De novo assembly of the complete mitochondrial genomes of two Camellia-oil tree species reveals their multibranch conformation and evolutionary relationships
Source: Sci Rep. 2025 Jan 23;15:2899. doi: 10.1038/s41598-025-86411-2 (PMC11754599; doi:10.1038/s41598-025-86411-2)
Supplement: Supplementary file 2 — Supplementary Material 2 [file 41598_2025_86411_MOESM2_ESM.pdf]

Supplementary Figures

(Figures S1-S6)

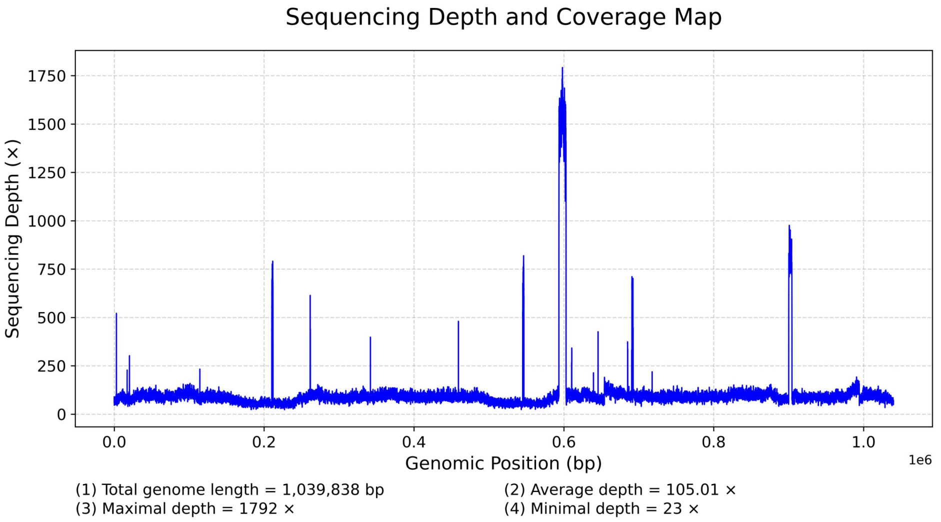

Figure S1. Depth and coverage of the assembled *C. oleifera* mitogenome using short-reads: the abscissa shows the genomic positions, and the ordinate shows the depth of mapped raw reads.

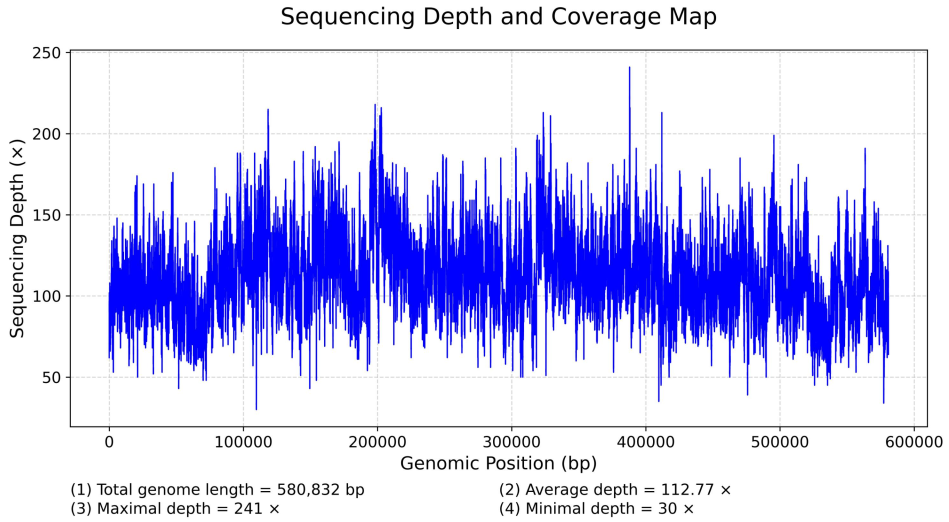

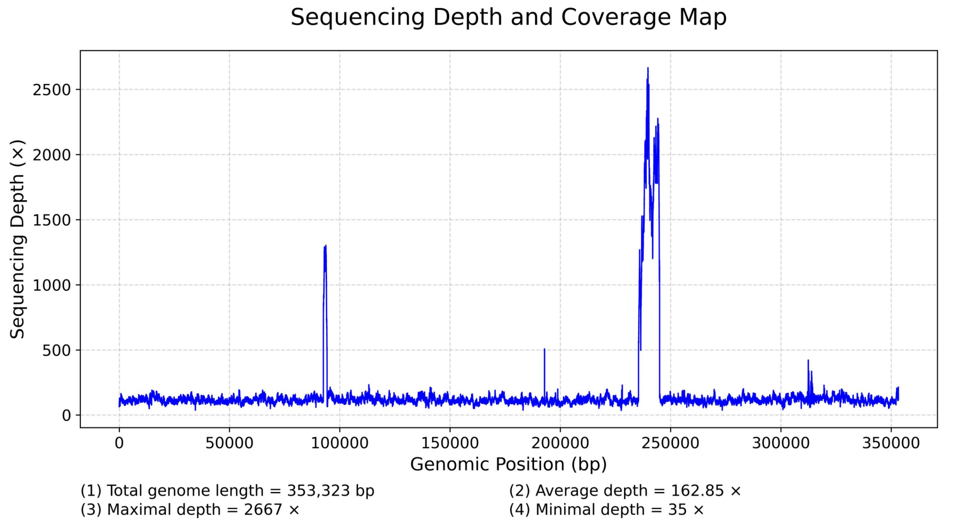

Figure S2. Depth and coverage of the assembled *C. lanceoleosa* mitogenome using short-reads: the abscissa shows the genomic positions, and the ordinate shows the depth of mapped raw reads.

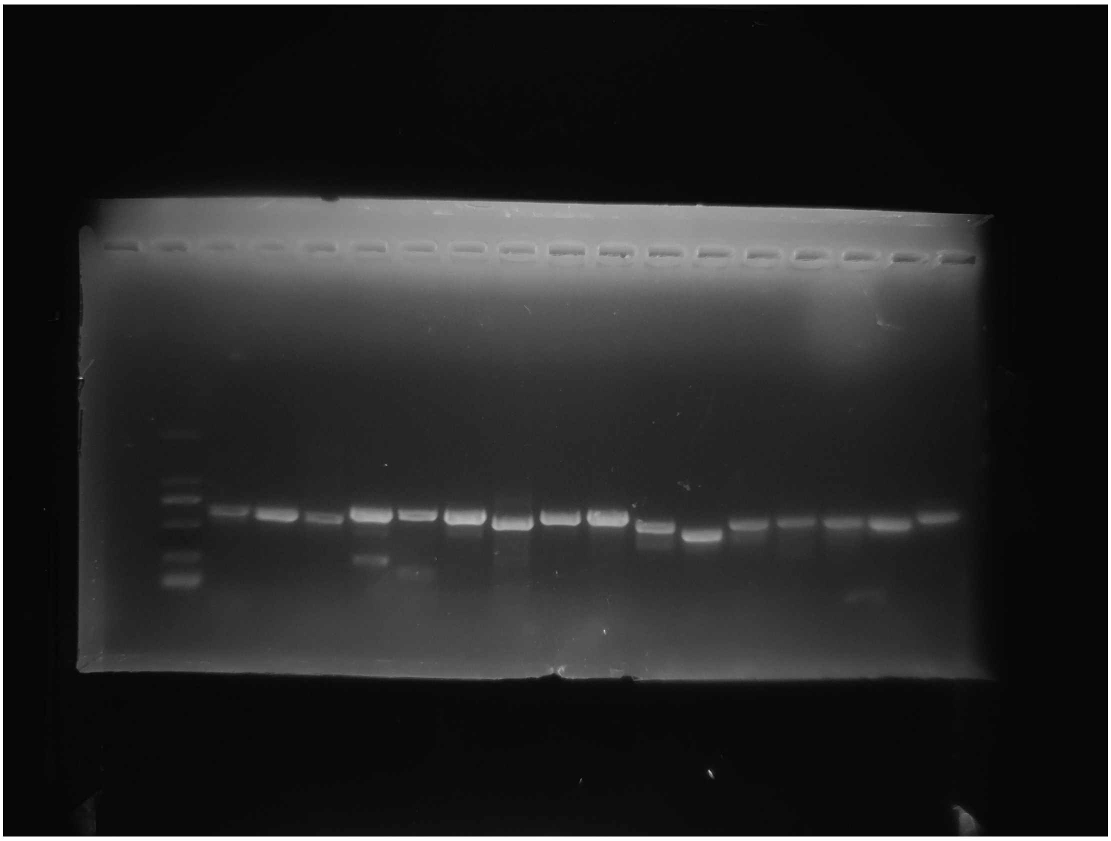

Figure S3. The raw Gel diagram of agarose gel electrophoresis for *Camellia oleifera.*

Figure S4 Graphical results of key linkage positions in the *Camellia oleifera* mitochondrial genome obtained from Sanger sequencing

10 20 30 40 50 60 70 80

ATGCGGTTGCGGAATGCTCCTTTTCACAAGGAGCAACTTGACCTATCTTCCCGCTAGGAAGAAAGACTGAGGGATCGTATTCCCAGAGA

c1c6(1>1000) → ATGCGGTTGCGGAATGCTCCTTTTCACAAGGAGCAACTTGACCTATCTTCCCGCTAGGAAGAAAGACTGAGGGATCGTATTCCCAGAGA

90 100 110 120 130 140 150 160 170

CTGACAGACGGGAGGGAAGAAAAGCTTATCGTTCGGGTCACCCACCCGGGAAGACCCTTCACTACACTGCTCGTAATATGCTTAGATAC

c1c6(1>1000) → CTGACAGACGGGAGGGAAGAAAAGCTTATCGTTCGGGTCACCCACCCGGGAAGACCCTTCACTACACTGCTCGTAATATGCTTAGATAC

180 190 200 210 220 230 240 250 260

GATTATACACCAGGGGGAGATCTAGATAGATTAACTCACTATTGACTCTTGGATGGAAAATAGCTACTGACTTAATGACTGGTGGCTTA

c1c6(1>1000) → GATTATACACCAGGGGGAGATCTAGATAGATTAACTCACTATTGACTCTTGGATGGAAAATAGCTACTGACTTAATGACTGGTGGCTTA
c1c6.ab1(32>586) →

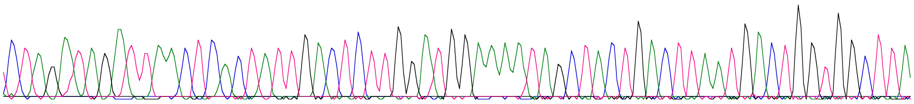

CTAGATAGATTAACTCACTATTGACTCTTGGATGGAAAATAGCTACTGACTTAATGACTGGTGGCTTA

270 280 290 300 310 320 330 340 350

CAATCAGGAATGCCTATTGGATGCAAATCCGGAATGACTCAAGGATGCCAATAGGCCCTGACTCAAGGATCCGAAGCAGTACTTATTGG

c1c6(1>1000) → CAATCAGGAATGCCTATTGGATGCAAATCCGGAATGACTCAAGGATGCCAATAGGCCCTGACTCAAGGATCCGAAGCAGTACTTATTGG
c1c6.ab1(32>586) →

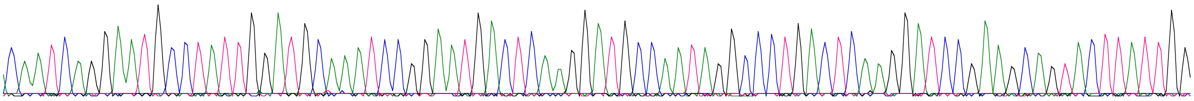

CAATCAGGAATGCCTATTGGATGCAAATCCGGAATGACTCAAGGATGCCAATAGGCCCTGACTCAAGGATCCGAAGCAGTACTTATTGG

360 370 380 390 400 410 420 430 440

TGGATTCCAGGCAGTACTTCCTCTGGGGATGACTCCTCAGATTAGCTGCCTTTTAGCAGATTCCGGGGGTCTCACTCCAGCTTGAACTA

c1c6(1>1000) → TGGATTCCAGGCAGTACTTCCTCTGGGGATGACTCCTCAGATTAGCTGCCTTTTAGCAGATTCCGGGGGTCTCACTCCAGCTTGAACTA
c1c6.ab1(32>586) →

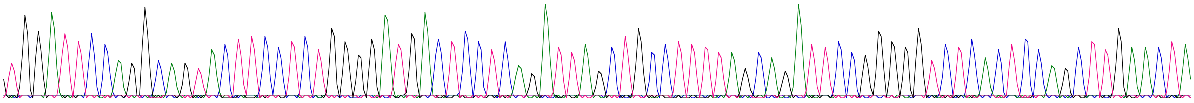

TGGATTCCAGGCAGTACTTCCTCTGGGGATGACTCCTCAGATTAGCTGCCTTTTAGCAGATTCCGGGGGTCTCACTCCAGCTTGAACTA

450 460 470 480 490 500 510 520 530

CTTGGATAGAACTAAGGGGCAAACTCCTTAGCGCTTTCTTTATACTTTGGCCTAGCTCTTCCGACCGAAGGTATTCATTCGTTGAGTTG

c1c6(1>1000) → CTTGGATAGAACTAAGGGGCAAACTCCTTAGCGCTTTCTTTATACTTTGGCCTAGCTCTTCCGACCGAAGGTATTCATTCGTTGAGTTG
c1c6.ab1(32>586) →

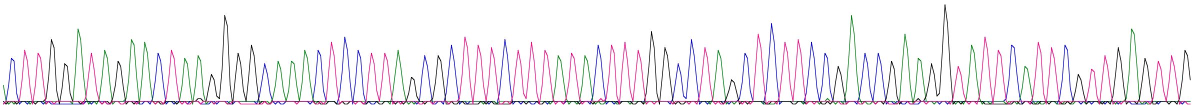

CTTGGATAGAACTAAGGGGCAAACTCCTTAGCGCTTTCTTTATACTTTGGCCTAGCTCTTCCGACCGAAGGTATTCATTCGTTGAGTTG

540 550 560 570 580 590 600 610 620

AAATGTCGACCGACAGGAAAGGGGGACTGACTTCAGTTCTCAGAGTGGGGCGGCGTGACCGCATGGCCCCTTGCTTGGCTTGCGGGATG

c1c6(1>1000) → AAATGTCGACCGACAGGAAAGGGGGACTGACTTCAGTTCTCAGAGTGGGGCGGCGTGACCGCATGGCCCCTTGCTTGGCTTGCGGGATG
c1c6.ab1(32>586) →

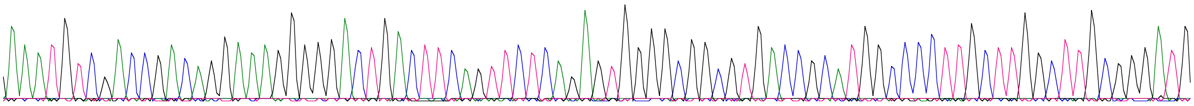

AAATGTCGACCGACAGGAAAGGGGGACTGACTTCAGTTCTCAGAGTGGGGCGGCGTGACCGCATGGCCCCTTGCTTGGCTTGCGGGATG

```
                630       640       650       660       670       680       690       700       710
                GGACTTCCTTTACTTAGCAACACGAAAGAGAAGGCAAAAAGAAAGCCTTCTAACTAGAGGGAGCCCGTTCTCTAGTGTCCACCTTATTC
c1c6(1>1000)       →  GGACTTCCTTTACTTAGCAACACGAAAGAGAAGGCAAAAAGAAAGCCTTCTAACTAGAGGGAGCCCGTTCTCTAGTGTCCACCTTATTC
c1c6.ab1(32>586)   →
```

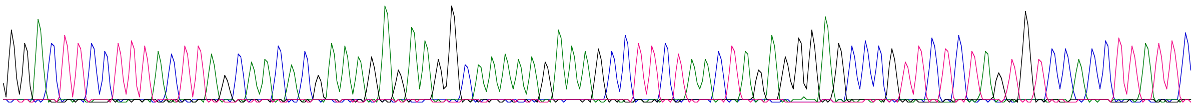

```
                GGACTTCCTTTACTTAGCAACACGAAAGAGAAGGCAAAAAGAAAGCCTTCTAACTAGAGGGAGCCCGTTCTCTAGTGTCCACCTTATTC

                 720       730       740       750       760       770       780       790       800
                TACTCACTTTAAACTGCCATTCTGTCTACATGAAAATGCCCATAGAAATGATATCACTACTAGACTGTGACTGACTCTACTACTACTTC
c1c6(1>1000)       →  TACTCACTTTAAACTGCCATTCTGTCTACATGAAAATGCCCATAGAAATGATATCACTACTAGACTGTGACTGACTCTACTACTACTTC
c1c6.ab1(32>586)   →
```

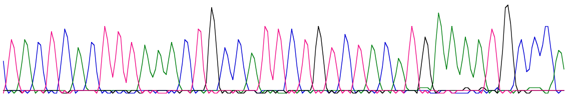

```
                TACTCACTTTAAACTGCCATTCTGTCTACATGAAAATGCCCA

                  810       820       830       840       850       860       870       880       890
                CTACTTCCGACTCCTAACCGATACTACATACTACTACAGATAGTTAGGAAAGGACACCCCTGACTCCATTCCCGGTGTAAATACGAAAA
c1c6(1>1000)       →  CTACTTCCGACTCCTAACCGATACTACATACTACTACAGATAGTTAGGAAAGGACACCCCTGACTCCATTCCCGGTGTAAATACGAAAA

                   900       910       920       930       940       950       960       970
                TAGTGTTTCTTTTTGAGGGAAATCTTTGAATTTGGAATGAAAAGAAGAGTAATGCTTCCTAGCACGGGACACAGAATAAGACCTCGAGG
c1c6(1>1000)       →  TAGTGTTTCTTTTTGAGGGAAATCTTTGAATTTGGAATGAAAAGAAGAGTAATGCTTCCTAGCACGGGACACAGAATAAGACCTCGAGG

                980       990       1000
                AGGAGATTGTCCCTTTCTACT
c1c6(1>1000)       →  AGGAGATTGTCCCTTTCTACT
```

10 20 30 40 50 60 70 80

ATGCGGTTGCGGAATGCTCCTTTTCACAAGGAGCAACTTGACCTATCTTCCCGCTAGGAAGAAAGACTGAGGGATCGTATTCCCAGAGA

c1c8(1>1000) ATGCGGTTGCGGAATGCTCCTTTTCACAAGGAGCAACTTGACCTATCTTCCCGCTAGGAAGAAAGACTGAGGGATCGTATTCCCAGAGA
c1c8.ab1(36>573)

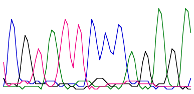

CGTATTCCCAGAGA

90 100 110 120 130 140 150 160 170

CTGACAGACGGGAGGGAAGAAAAGCTTATCGTTCGGGTCACCCACCCGGGAAGACCCTTCACTACACTGCTCGTAATATGCTTAGATAC

c1c8(1>1000) CTGACAGACGGGAGGGAAGAAAAGCTTATCGTTCGGGTCACCCACCCGGGAAGACCCTTCACTACACTGCTCGTAATATGCTTAGATAC
c1c8.ab1(36>573)

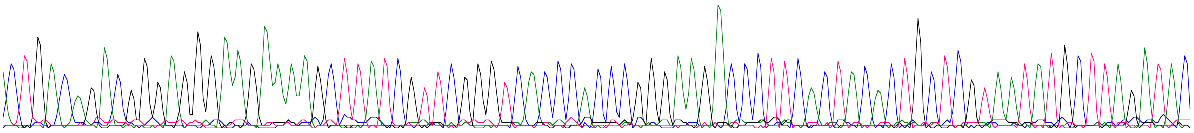

CTGACAGACGGGAGGGAAGAAAAGCTTATCGTTCGGGTCACCCACCCGGGAAGACCCTTCACTACACTGCTCGTAATATGCTTAGATAC

180 190 200 210 220 230 240 250 260

GATTATACACCAGGGGGAGATCTAGATAGATTAACTCACTATTGACTCTTGGATGGAAAATAGCTACTGACTTAATGACTGGTGGCTTA

c1c8(1>1000) GATTATACACCAGGGGGAGATCTAGATAGATTAACTCACTATTGACTCTTGGATGGAAAATAGCTACTGACTTAATGACTGGTGGCTTA
c1c8.ab1(36>573)

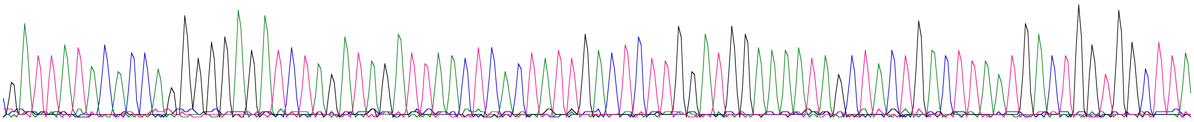

GATTATACACCAGGGGGAGATCTAGATAGATTAACTCACTATTGACTCTTGGATGGAAAATAGCTACTGACTTAATGACTGGTGGCTTA

270 280 290 300 310 320 330 340 350

CAATCAGGAATGCCTATTGGATGCAAATCCGGAATGACTCAAGGATGCCAATAGGCCCTGACTCAAGGATCCGAAGCAGTACTTATTGG

c1c8(1>1000) CAATCAGGAATGCCTATTGGATGCAAATCCGGAATGACTCAAGGATGCCAATAGGCCCTGACTCAAGGATCCGAAGCAGTACTTATTGG
c1c8.ab1(36>573)

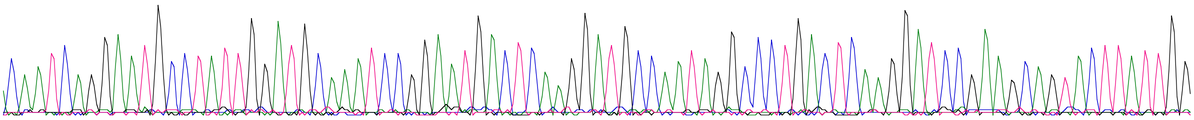

CAATCAGGAATGCCTATTGGATGCAAATCCGGAATGACTCAAGGATGCCAATAGGCCCTGACTCAAGGATCCGAAGCAGTACTTATTGG

360 370 380 390 400 410 420 430 440

TGGATTCCAGGCAGTACTTCCTCTGGGGATGACTCCTCAGATTAGCTGCCTTTTAGCAGATTCCGGGGGTCTCACTCCAGCTTGAACTA

c1c8(1>1000) TGGATTCCAGGCAGTACTTCCTCTGGGGATGACTCCTCAGATTAGCTGCCTTTTAGCAGATTCCGGGGGTCTCACTCCAGCTTGAACTA
c1c8.ab1(36>573)

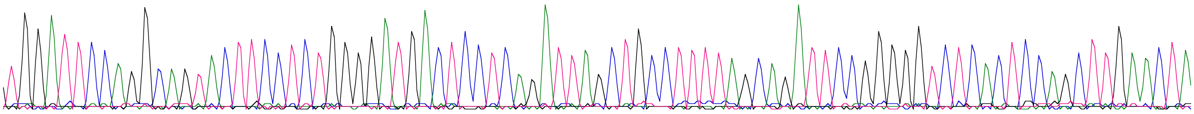

TGGATTCCAGGCAGTACTTCCTCTGGGGATGACTCCTCAGATTAGCTGCCTTTTAGCAGATTCCGGGGGTCTCACTCCAGCTTGAACTA

450 460 470 480 490 500 510 520 530

CTTGGATAGAACTAAGGGGCAAACTCCTTAGCGCTTTCTTTATACTTTGGCCTAGGGGTCGGAAGAGCTGGAGTTATGGAACCTAGCTC

c1c8(1>1000) CTTGGATAGAACTAAGGGGCAAACTCCTTAGCGCTTTCTTTATACTTTGGCCTAGGGGTCGGAAGAGCTGGAGTTATGGAACCTAGCTC
c1c8.ab1(36>573)

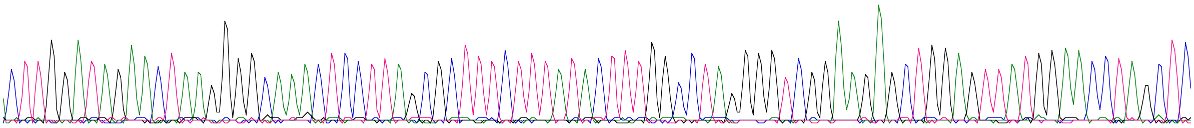

CTTGGATAGAACTAAGGGGCAAACTCCTTAGCGCTTTCTTTATACTTTGGCCTAGGGGTCGGAAGAGCTGGAGTTATGGAACCTAGCTC

```
                 540       550       560       570       580       590       600       610       620
                 CTGCCGTCCGGCCCACGATCAACAAGATAATGAATGAAGGCGGGGGAAGGGAAGGAAGTAAGATACGTACTAGCAACCCGAAGGCATCC
c1c8(1>1000)       → CTGCCGTCCGGCCCACGATCAACAAGATAATGAATGAAGGCGGGGGAAGGGAAGGAAGTAAGATACGTACTAGCAACCCGAAGGCATCC
c1c8.ab1(36>573)   →
```

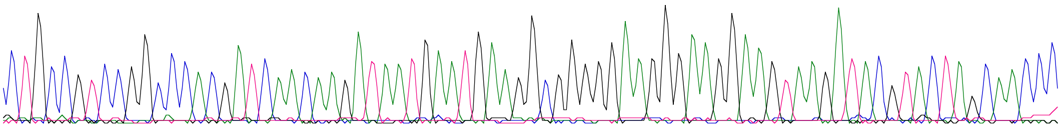

```
                 CTGCCGTCCGGCCCACGATCAACAAGATAATGAATGAAGGCGGGGGAAGGGAAGGAAGTAAGATACGTACTAGCAACCC

                 630       640       650       660       670       680       690       700       710
                 TTGTTCTTTGTGTTCTTTGGCAGGACAGATGGGTACTGAGCTAAGGGGAAGCCTTTCAATCCAAGCAAGTAAAGGCGAAGCCTTTCTGC
c1c8(1>1000)       → TTGTTCTTTGTGTTCTTTGGCAGGACAGATGGGTACTGAGCTAAGGGGAAGCCTTTCAATCCAAGCAAGTAAAGGCGAAGCCTTTCTGC

                 720       730       740       750       760       770       780       790       800
                 AAGCCAGTATCGCCACTACGATTGAGAGAACTTCCTCTTTCCGGAAAGAGGGGGAAACTCCCCCAGCCAAGACAGGCAAGACTCGATCC
c1c8(1>1000)       → AAGCCAGTATCGCCACTACGATTGAGAGAACTTCCTCTTTCCGGAAAGAGGGGGAAACTCCCCCAGCCAAGACAGGCAAGACTCGATCC

                 810       820       830       840       850       860       870       880       890
                 CTCCTAGGCCGGTGTCAAACCCTCCACCTAAGTTGATCAAAAAGCACACTGTGAGTACCACATGGGCACGCCGGGACACTGGACTGATA
c1c8(1>1000)       → CTCCTAGGCCGGTGTCAAACCCTCCACCTAAGTTGATCAAAAAGCACACTGTGAGTACCACATGGGCACGCCGGGACACTGGACTGATA

                 900       910       920       930       940       950       960       970
                 GATGCTACAACCTAAGGCACAAGATTCAAGACTTGATTGACCAGCACTTATTGGAATTCCAGGAAGGAGGAAGCAACCCCGTCATTACA
c1c8(1>1000)       → GATGCTACAACCTAAGGCACAAGATTCAAGACTTGATTGACCAGCACTTATTGGAATTCCAGGAAGGAGGAAGCAACCCCGTCATTACA

                 980       990       1000
                 AAGAATCCATCCCCTACGCGT
c1c8(1>1000)       → AAGAATCCATCCCCTACGCGT
```

10 20 30 40 50 60 70 80

TTTCGGTAAGCATCCAGTATCTCAGCAAATGAACATTTCTCTAAGCTTATCCTGTGACTTATACGTCGTTTGAAAGCTCCTTCAAGGA

c1c12(1>1000) TTTCGGTAAGCATCCAGTATCTCAGCAAATGAACATTTCTCTAAGCTTATCCTGTGACTTATACGTCGTTTGAAAGCTCCTTCAAGGA

90 100 110 120 130 140 150 160 170

TCCAACGAATAGCTAAGGTTTGTTGACGATCCCTGGCTACAATCCCAGGGACATCATAAATAGTACCTGCTATTCCTACTTTTTCCAC

c1c12(1>1000) TCCAACGAATAGCTAAGGTTTGTTGACGATCCCTGGCTACAATCCCAGGGACATCATAAATAGTACCTGCTATTCCTACTTTTTCCAC
c1c12.ab1(20>569)

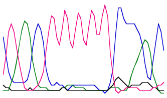

TACTTTTTCCAC

180 190 200 210 220 230 240 250 260

TTCGCATATGGGCTTTATATTCTCTAGGGCGTCAACCATAAGTTTGATTACATCGCGTTCAGTTCGAGCTGGGCGATGAAAAGTTTGA

c1c12(1>1000) TTCGCATATGGGCTTTATATTCTCTAGGGCGTCAACCATAAGTTTGATTACATCGCGTTCAGTTCGAGCTGGGCGATGAAAAGTTTGA
c1c12.ab1(20>569)

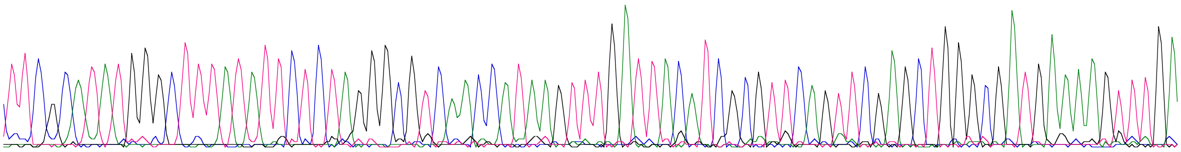

TTCGCATATGGGCTTTATATTCTCTAGGGCGTCAACCATAAGTTTGATTACATCGCGTTCAGTTCGAGCTGGGCGATGAAAAGTTTGA

270 280 290 300 310 320 330 340 350

TAAACAATAGCACGAACTCTTGTTCTTTTACCTTCTTTCATGCGAAAGTTGACCAACTTCTTGATCAATTGTTTTTGCTCACCATCCA

c1c12(1>1000) TAAACAATAGCACGAACTCTTGTTCTTTTACCTTCTTTCATGCGAAAGTTGACCAACTTCTTGATCAATTGTTTTTGCTCACCATCCA
c1c12.ab1(20>569)

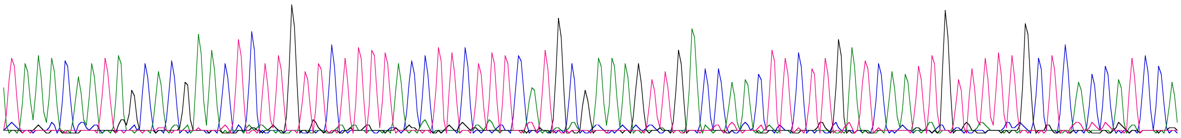

TAAACAATAGCACGAACTCTTGTTCTTTTACCTTCTTTCATGCGAAAGTTGACCAACTTCTTGATCAATTGTTTTTGCTCACCATCCA

360 370 380 390 400 410 420 430 440

AGCCCCCCATATAGCTGAGAATCTCCGAGCAATTGGAAGCCGCTTTCGATGACGAGGCCGACAAATTAATATTACGCGATCCTTACTG

c1c12(1>1000) AGCCCCCCATATAGCTGAGAATCTCCGAGCAATTGGAAGCCGCTTTCGATGACGAGGCCGACAAATTAATATTACGCGATCCTTACTG
c1c12.ab1(20>569)

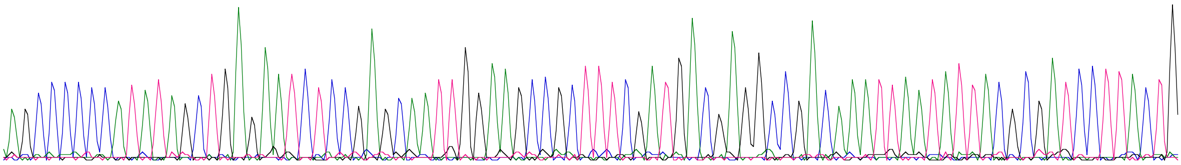

AGCCCCCCATATAGCTGAGAATCTCCGAGCAATTGGAAGCCGCTTTCGATGACGAGGCCGACAAATTAATATTACGCGATCCTTACTG

450 460 470 480 490 500 510 520

TCCATCTTTATCAGCCAACTCCCCGGTTTCCATCTTTCAAAGTCGACCATCCCTCATAGCTTTCACTAATCGGGGAAGTGATCCTTAA

c1c12(1>1000) TCCATCTTTATCAGCCAACTCCCCGGTTTCCATCTTTCAAAGTCGACCATCCCTCATAGCTTTCACTAATCGGGGAAGTGATCCTTAA
c1c12.ab1(20>569)

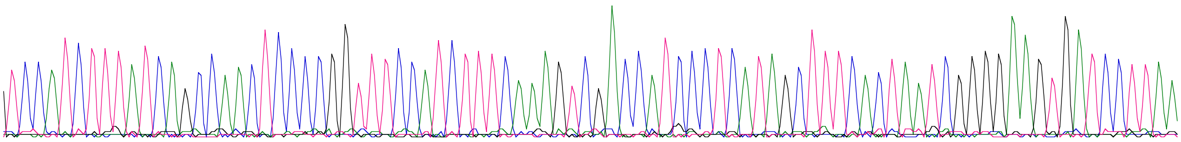

TCCATCTTTATCAGCCAACTCCCCGGTTTCCATCTTTCAAAGTCGACCATCCCTCATAGCTTTCACTAATCGGGGAAGTGATCCTTAA

530 540 550 560 570 580 590 600 610

TGAGGGATTTCAGGAGAGCTGGGTCTTTGACGGCCTCGTTCCAGCTTCGGAGGGTGTCTGCATCCATCTCCGAAGAGATCTCCAAATC

c1c12(1>1000) TGAGGGATTTCAGGAGAGCTGGGTCTTTGACGGCCTCGTTCCAGCTTCGGAGGGTGTCTGCATCCATCTCCGAAGAGATCTCCAAATC

c1c12.ab1(20>569)

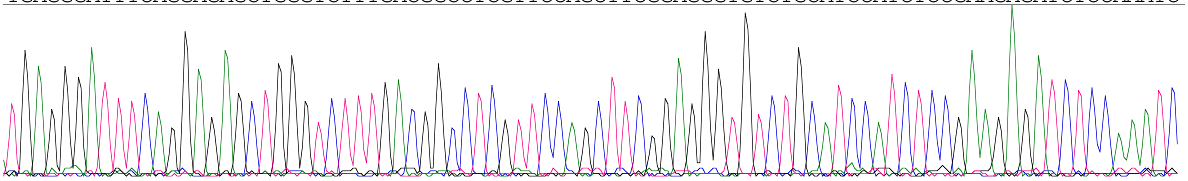

TGAGGGATTTCAGGAGAGCTGGGTCTTTGACGGCCTCGTTCCAGCTTCGGAGGGTGTCTGCATCCATCTCCGAAGAGATCTCCAAATC

620 630 640 650 660 670 680 690 700

CCCCATCAAGTTCCTTGCCCCCTGGGAGTAGATTTCGTCTTGCTCGGGAAAGCGAGACATCCCCCTCCGCCACGGTTGACGTTCACAA

c1c12(1>1000) CCCCATCAAGTTCCTTGCCCCCTGGGAGTAGATTTCGTCTTGCTCGGGAAAGCGAGACATCCCCCTCCGCCACGGTTGACGTTCACAA

c1c12.ab1(20>569)

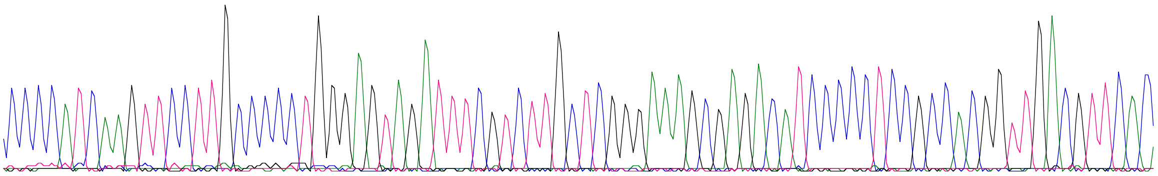

CCCCATCAAGTTCCTTGCCCCCTGGGAGTAGATTTCGTCTTGCTCGGGAAAGCGAGACATCCCCCTCCGCCACGGTTGACGTTCACA

710 720 730 740 750 760 770 780 790

CGGTGCCGAATAAGTTCACCTATTTGAGCGCGTATTGACCGGAGAAGTTCCTCCCCTTGGTGAACTTCCGCCGGAGCATTCTCCGGCG

c1c12(1>1000) CGGTGCCGAATAAGTTCACCTATTTGAGCGCGTATTGACCGGAGAAGTTCCTCCCCTTGGTGAACTTCCGCCGGAGCATTCTCCGGCG

800 810 820 830 840 850 860 870 880

CAGGCTCAGCAGGAGGGGCCGGCGCAGGCTCAGCAGGAGGGGCCGGCTCAGGCTCAGCAGGAGGGGGGCCGTTGAGATCTAGCCCCGG

c1c12(1>1000) CAGGCTCAGCAGGAGGGGCCGGCGCAGGCTCAGCAGGAGGGGCCGGCTCAGGCTCAGCAGGAGGGGGGCCGTTGAGATCTAGCCCCGG

890 900 910 920 930 940 950 960

CTCAGGAGCTGGAGGTTGGTTTAGATCGAGCTCCGGGCGCCCCGAACTCGAAGGCCCGGCATCGCTTCCGCCGCCTCCAACGGAAAGA

c1c12(1>1000) CTCAGGAGCTGGAGGTTGGTTTAGATCGAGCTCCGGGCGCCCCGAACTCGAAGGCCCGGCATCGCTTCCGCCGCCTCCAACGGAAAGA

970 980 990 1000

GGGAGTCCGTCCATGGAAGACAGAATCCAATC

c1c12(1>1000) GGGAGTCCGTCCATGGAAGACAGAATCCAATC

```
                  10        20        30        40        50        60        70        80
                  CATGATCTGGCCTGGTCGACCCAATCATGATATTGAAGGATGGACCTTTTCTCGAAAAACTCCGGATCCCGAGAAGAGTTGAAGACG
c2c11(1>1000)  →  CATGATCTGGCCTGGTCGACCCAATCATGATATTGAAGGATGGACCTTTTCTCGAAAAACTCCGGATCCCGAGAAGAGTTGAAGACG

                  90        100       110       120       130       140       150       160       170
                  GTGCAAGATCGAATCCTCTTACGTTACTTCGAATGGAATCTTAGCCCGCCTCACTTGCTTTCTTTACTGCATAAGGGCATAAGCGAAG
c2c11(1>1000)  →  GTGCAAGATCGAATCCTCTTACGTTACTTCGAATGGAATCTTAGCCCGCCTCACTTGCTTTCTTTACTGCATAAGGGCATAAGCGAAG

                  180       190       200       210       220       230       240       250       260
                  TCAAGGGATTTCCTTCTAACTAGTGGCTGAGAGTAAGCAAGCTACCTTCATTCAACAGATTTGTGGTTGAGTCAATAACATGCTCTGG
c2c11(1>1000)  →  TCAAGGGATTTCCTTCTAACTAGTGGCTGAGAGTAAGCAAGCTACCTTCATTCAACAGATTTGTGGTTGAGTCAATAACATGCTCTGG
c2c11.ab1(19>649) →
```

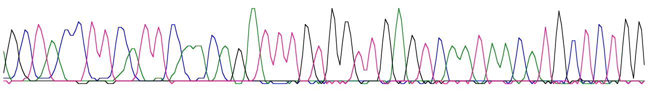


```
                  270       280       290       300       310       320       330       340       350
                  GTCGGGAATCTTTGCTCTTCTCTTTTGCATTTCCGCTGCCTGCGTTCTTCTTCGTGTCCGTCCGTATCGCAAAGCTGGTCGACGCCAG
c2c11(1>1000)  →  GTCGGGAATCTTTGCTCTTCTCTTTTGCATTTCCGCTGCCTGCGTTCTTCTTCGTGTCCGTCCGTATCGCAAAGCTGGTCGACGCCAG
c2c11.ab1(19>649) →
```

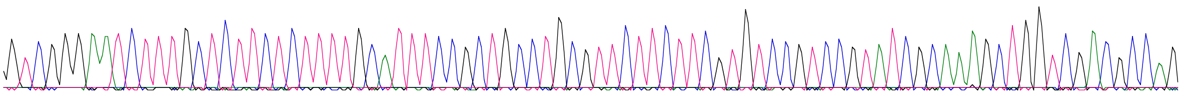


```
                  360       370       380       390       400       410       420       430       440
                  CTGTTGAAAATAGGGGGCCAACCAAGACCCCCTAATAAAGCTTTGTTCGATAAAGCAAACGATTCGTTCCGACAACTTCGGACCAGAT
c2c11(1>1000)  →  CTGTTGAAAATAGGGGGCCAACCAAGACCCCCTAATAAAGCTTTGTTCGATAAAGCAAACGATTCGTTCCGACAACTTCGGACCAGAT
c2c11.ab1(19>649) →
```

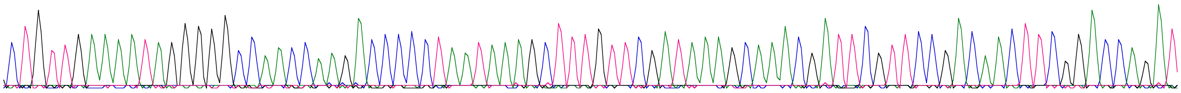


```
                  450       460       470       480       490       500       510       520
                  TAATCCCTTTGAATTAGCCGATCTTACAAGATCGATCGGGCGGGCTGGTTGGGAAGGGGTGATTCGCGGAGAAAAGAATTGCTGAGAG
c2c11(1>1000)  →  TAATCCCTTTGAATTAGCCGATCTTACAAGATCGATCGGGCGGGCTGGTTGGGAAGGGGTGATTCGCGGAGAAAAGAATTGCTGAGAG
c2c11.ab1(19>649) →
```

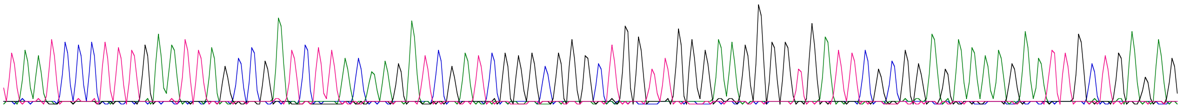


```
                  530       540       550       560       570       580       590       600       610
                  ATAGATTCTGGTCAGGACCCTCTAATTTAGGGATTAGTCAGAAAGCAAGTCTCTCTCACGAAACAACAGAGAATTTTTTATCCTGTCT
c2c11(1>1000)  →  ATAGATTCTGGTCAGGACCCTCTAATTTAGGGATTAGTCAGAAAGCAAGTCTCTCTCACGAAACAACAGAGAATTTTTTATCCTGTCT
c2c11.ab1(19>649) →
```

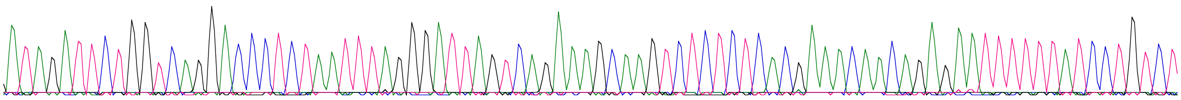

```
                       620       630       640       650       660       670       680       690       700
                       AACCTCTGCTACTGCAGGTCTGGTATGATAGAATCCCTAGGTTATCAGTTCACGAAGTGCAACGAGTTCAGTAGGGAACGGCCTGCGA
c2c11(1>1000)      →   AACCTCTGCTACTGCAGGTCTGGTATGATAGAATCCCTAGGTTATCAGTTCACGAAGTGCAACGAGTTCAGTAGGGAACGGCCTGCGA
c2c11.ab1(19>649)  →
```

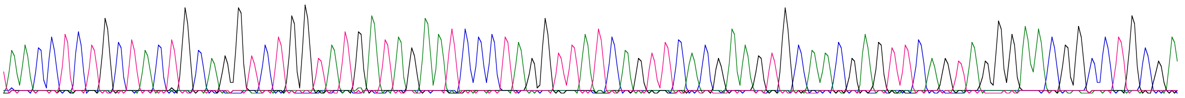


```
                       710       720       730       740       750       760       770       780       790
                       GTCATTTTTGGGAAGAGAGCTTAAATAGCTATTTTGAACTATACCATTTAGTAAACAGTCACAAAGGGTTTGCTTTAATTTCATACCT
c2c11(1>1000)      →   GTCATTTTTGGGAAGAGAGCTTAAATAGCTATTTTGAACTATACCATTTAGTAAACAGTCACAAAGGGTTTGCTTTAATTTCATACCT
c2c11.ab1(19>649)  →
```

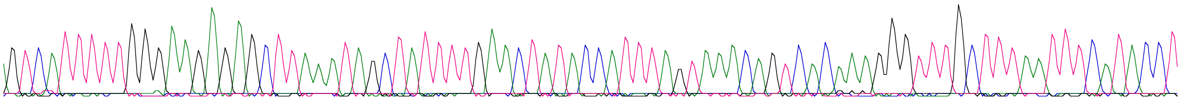


```
                       800       810       820       830       840       850       860       870       880
                       TTCAATCATCAGTCATAATTAGGAAGAGAAGACAGAAAAGCAGCCGGTAGAATAGCTCACTCCATTGGGGAAAGAAAAAAAGTTAGTG
c2c11(1>1000)      →   TTCAATCATCAGTCATAATTAGGAAGAGAAGACAGAAAAGCAGCCGGTAGAATAGCTCACTCCATTGGGGAAAGAAAAAAAGTTAGTG
c2c11.ab1(19>649)  →
```

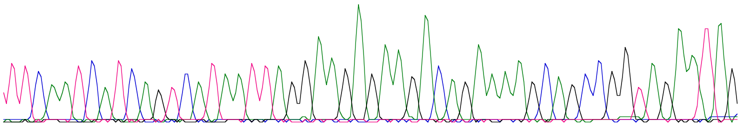


```
                       890       900       910       920       930       940       950       960
                       ACCTCATCCCCTTCAGGTGGAATCAGAGAGAGAGAGGAGCGGAACAATTGGAAGATCGCATCGGGACACTTTAGCGGGAACAGATCCT
c2c11(1>1000)      →   ACCTCATCCCCTTCAGGTGGAATCAGAGAGAGAGAGGAGCGGAACAATTGGAAGATCGCATCGGGACACTTTAGCGGGAACAGATCCT

                       970       980       990       1000
                       CTATATTAGCTCTAGGGGACTAGGGCTCCCTT
c2c11(1>1000)      →   CTATATTAGCTCTAGGGGACTAGGGCTCCCTT
```

10 20 30 40 50 60 70 80

AAGGCAGGGAGTGTGACAACGTACACGCTTCCCTTTACTCCTTACTCCATAGGGCCTTCTCATCAGTTGCAGGGTTTGGTGGCCCGAA

c4c12(1>1000) → AAGGCAGGGAGTGTGACAACGTACACGCTTCCCTTTACTCCTTACTCCATAGGGCCTTCTCATCAGTTGCAGGGTTTGGTGGCCCGAA

90 100 110 120 130 140 150 160 170

ATTGACTTGGCTTCGCCAAAAGGCCTCATCTCTAGAAGCCCGGCTCGCGAGGCGTCCCTCTCGCAGTAGGGTTCCTCGCCTCGCTCTT

c4c12(1>1000) → ATTGACTTGGCTTCGCCAAAAGGCCTCATCTCTAGAAGCCCGGCTCGCGAGGCGTCCCTCTCGCAGTAGGGTTCCTCGCCTCGCTCTT

180 190 200 210 220 230 240 250 260

GTGACATGCTATGTTTCCCCTCTCCATTCCCAAGTAAAGGGTGAGTCCCATGCGTCAGTTTGTGGATCGCGGTCTCACGCACTAATCC

c4c12(1>1000) → GTGACATGCTATGTTTCCCCTCTCCATTCCCAAGTAAAGGGTGAGTCCCATGCGTCAGTTTGTGGATCGCGGTCTCACGCACTAATCC

270 280 290 300 310 320 330 340 350

CTACAGGTGCCCGGAGTAGGCCGGCCGCCCTACCTAAACCAATCATCATATCGGTCCCTAGGCCCCATTGCTGGAAAGGCTCGGCTTC

c4c12(1>1000) → CTACAGGTGCCCGGAGTAGGCCGGCCGCCCTACCTAAACCAATCATCATATCGGTCCCTAGGCCCCATTGCTGGAAAGGCTCGGCTTC

360 370 380 390 400 410 420 430 440

AAAACCGTACGTGGAGCTTCCGCCTCATACGGCTCCTCTAGGGATGGAGGTAGGCCCAGCCCAGGCTTGTGCGGTTAAGGTTGTTGTA

c4c12(1>1000) → AAAACCGTACGTGGAGCTTCCGCCTCATACGGCTCCTCTAGGGATGGAGGTAGGCCCAGCCCAGGCTTGTGCGGTTAAGGTTGTTGTA
c4c12.ab1(28>564) →

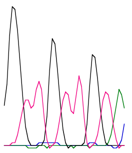

450 460 470 480 490 500 510 520

CACGACCTCATAAGAAAAGAATGAAAAAAACGATGTTTCCATCTTTCAGAGCGGACCAGTTTTCACTAATCGGGGAAGTGATCCTTAA

c4c12(1>1000) → CACGACCTCATAAGAAAAGAATGAAAAAAACGATGTTTCCATCTTTCAGAGCGGACCAGTTTTCACTAATCGGGGAAGTGATCCTTAA
c4c12.ab1(28>564) →

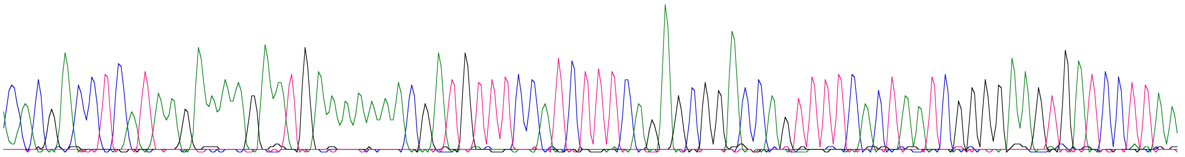

530 540 550 560 570 580 590 600 610

TGAGGGATTTCAGGAGAGCTGGGTCTTTGACGGCCTCGTTCCAGCTTCGGAGGGTGTCTGCATCCATCTCCGAAGAGATCTCCAAATC

c4c12(1>1000) → TGAGGGATTTCAGGAGAGCTGGGTCTTTGACGGCCTCGTTCCAGCTTCGGAGGGTGTCTGCATCCATCTCCGAAGAGATCTCCAAATC
c4c12.ab1(28>564) →

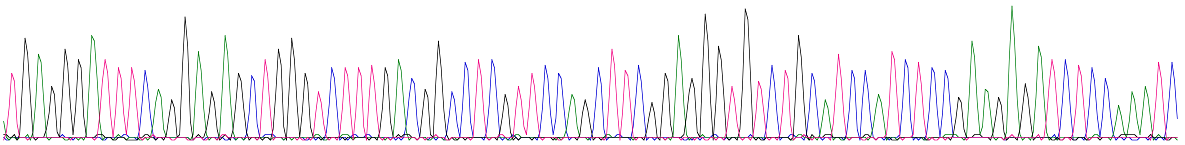

620 630 640 650 660 670 680 690 700

CCCCATCAAGTTCCTTGCCCCCTGGGAGTAGATTTCGTCTTGCTCGGGAAAGCGAGACATCCCCCTCCGCCACGGTTGACGTTCACAA

c4c12(1>1000) → CCCCATCAAGTTCCTTGCCCCCTGGGAGTAGATTTCGTCTTGCTCGGGAAAGCGAGACATCCCCCTCCGCCACGGTTGACGTTCACAA
c4c12.ab1(28>564) →

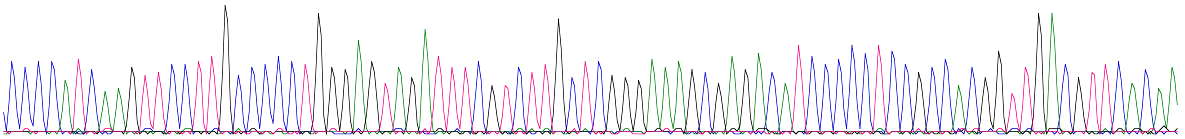

710 720 730 740 750 760 770 780 790

CGGTGCCGAATAAGTTCACCTATTTGAGCGCGTATTGACCGGAGAAGTTCCTCCCCTTGGTGAACTTCCGCCGGAGCATTCTCCGGCG

c4c12(1>1000) → CGGTGCCGAATAAGTTCACCTATTTGAGCGCGTATTGACCGGAGAAGTTCCTCCCCTTGGTGAACTTCCGCCGGAGCATTCTCCGGCG
c4c12.ab1(28>564) →

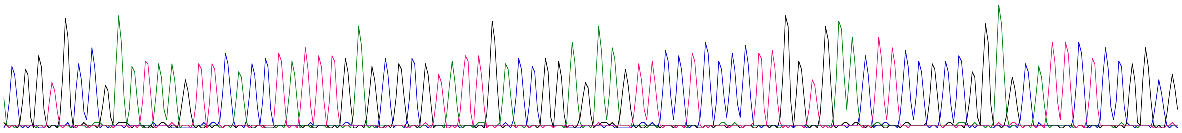

CGGTGCCGAATAAGTTCACCTATTTGAGCGCGTATTGACCGGAGAAGTTCCTCCCCTTGGTGAACTTCCGCCGGAGCATTCTCCGGCG

800 810 820 830 840 850 860 870 880

CAGGCTCAGCAGGAGGGGCCGGCGCAGGCTCAGCAGGAGGGGCCGGCTCAGGCTCAGCAGGAGGGGGGCCGTTGAGATCTAGCCCCGG

c4c12(1>1000) → CAGGCTCAGCAGGAGGGGCCGGCGCAGGCTCAGCAGGAGGGGCCGGCTCAGGCTCAGCAGGAGGGGGGCCGTTGAGATCTAGCCCCGG
c4c12.ab1(28>564) →

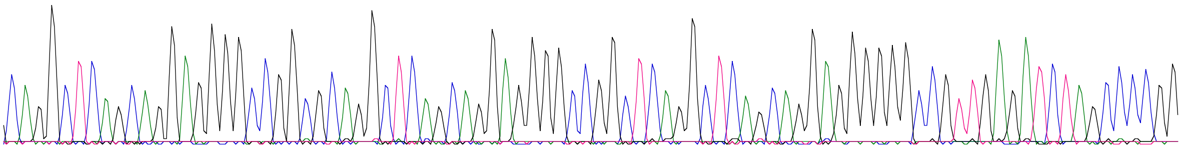

CAGGCTCAGCAGGAGGGGCCGGCGCAGGCTCAGCAGGAGGGGCCGGCTCAGGCTCAGCAGGAGGGGGGCCGTTGAGATCTAGCCCCGG

890 900 910 920 930 940 950 960

CTCAGGAGCTGGAGGTTGGTTTAGATCGAGCTCCGGGCGCCCCGAACTCGAAGGCCCGGCATCGCTTCCGCCGCCTCCAACGGAAAGA

c4c12(1>1000) → CTCAGGAGCTGGAGGTTGGTTTAGATCGAGCTCCGGGCGCCCCGAACTCGAAGGCCCGGCATCGCTTCCGCCGCCTCCAACGGAAAGA
c4c12.ab1(28>564) →

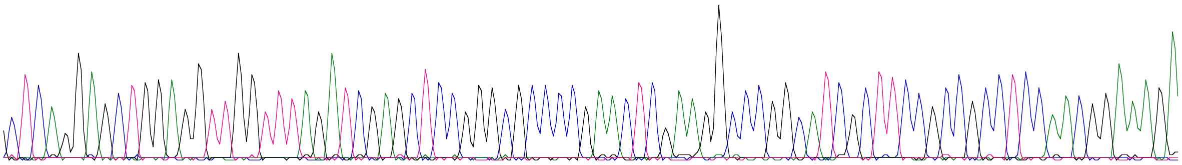

CTCAGGAGCTGGAGGTTGGTTTAGATCGAGCTCCGGGCGCCCCGAACTCGAAGGCCCGGCATCGCTTCCGCCGCCTCCAACGGAAAGA

970 980 990 1000

GGGAGTCCGTCCATGGAAGACAGAATCCAATC

c4c12(1>1000) → GGGAGTCCGTCCATGGAAGACAGAATCCAATC

10 20 30 40 50 60 70 80

AGCAGCACTTCCACTGCTAGGACTCTTCACACAAGACAAGACGGTCGGGCTGACCCAAGGGATCAATCAACTGGGACTTCTCCCTCTCG

c5c2(1>1000) → AGCAGCACTTCCACTGCTAGGACTCTTCACACAAGACAAGACGGTCGGGCTGACCCAAGGGATCAATCAACTGGGACTTCTCCCTCTCG

90 100 110 120 130 140 150 160 170

CTCTCTTCTCATTGGATTCTTCTCATTCCACTTTGAAATAGGAACAACAACAACAAGAAGATGGCGCACGCGGGTGCTAGTATAAATGC

c5c2(1>1000) → CTCTCTTCTCATTGGATTCTTCTCATTCCACTTTGAAATAGGAACAACAACAACAAGAAGATGGCGCACGCGGGTGCTAGTATAAATGC

180 190 200 210 220 230 240 250 260

TGCACCTCTAGTGGTTTCCTCCGACGAAGCTGCCTTTGTTTTTGATGCCGAAGCTGTCGATGTTGTTTCTTTGGTGACCTTTCCCCGTT

c5c2(1>1000) → TGCACCTCTAGTGGTTTCCTCCGACGAAGCTGCCTTTGTTTTTGATGCCGAAGCTGTCGATGTTGTTTCTTTGGTGACCTTTCCCCGTT

270 280 290 300 310 320 330 340 350

CCACAGTGGGCTAGCCTCAATAGAAATTCTCTTGCCACTTTCTGTAGTGAGAAAAGCTCTTTGATAGGCTTTCACGCCAATTCAGTACT

c5c2(1>1000) → CCACAGTGGGCTAGCCTCAATAGAAATTCTCTTGCCACTTTCTGTAGTGAGAAAAGCTCTTTGATAGGCTTTCACGCCAATTCAGTACT
c5c2.ab1(21>645) →

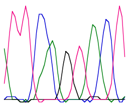

TTCAGTACT

360 370 380 390 400 410 420 430 440

CGTAATCAACGACTTCGATAAAAGGGCCGAAACCTGGCCTAGGAGCAAAAAACCTAAACACAGAAGAAGCGCCTTCAAGCTTAGCCCGC

c5c2(1>1000) → CGTAATCAACGACTTCGATAAAAGGGCCGAAACCTGGCCTAGGAGCAAAAAACCTAAACACAGAAGAAGCGCCTTCAAGCTTAGCCCGC
c5c2.ab1(21>645) →

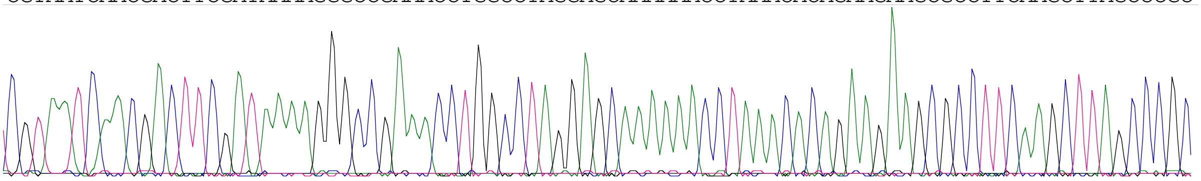

CGTAATCAACGACTTCGATAAAAGGGCCGAAACCTGGCCTAGGAGCAAAAAACCTAAACACAGAAGAAGCGCCTTCAAGCTTAGCCCGC

450 460 470 480 490 500 510 520 530

TCACTCCTACCCGTTCAGTCCTAGCTGTCTGTCCTTGCTTGGCTTGCCTGCCTGGGAATTCCAGTTGATTAGAGTTCCCACTGGCTGGA

c5c2(1>1000) → TCACTCCTACCCGTTCAGTCCTAGCTGTCTGTCCTTGCTTGGCTTGCCTGCCTGGGAATTCCAGTTGATTAGAGTTCCCACTGGCTGGA
c5c2.ab1(21>645) →

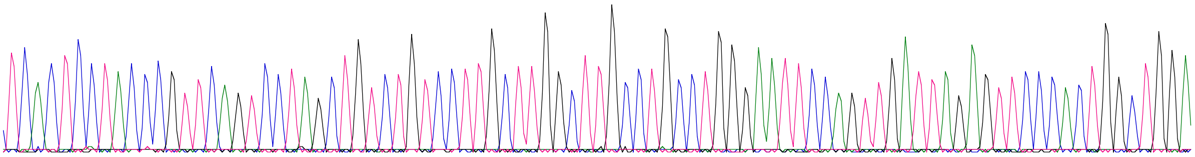

TCACTCCTACCCGTTCAGTCCTAGCTGTCTGTCCTTGCTTGGCTTGCCTGCCTGGGAATTCCAGTTGATTAGAGTTCCCACTGGCTGGA

540 550 560 570 580 590 600 610 620

CGGTGAAATCTTTAGTTGGTTTCCGACAGCAACCCTTCTTTATCTATAGTCCCGTTCTTCGGTTGATTTACTTGATTGGCTAGTCTCAC

c5c2(1>1000) → CGGTGAAATCTTTAGTTGGTTTCCGACAGCAACCCTTCTTTATCTATAGTCCCGTTCTTCGGTTGATTTACTTGATTGGCTAGTCTCAC
c5c2.ab1(21>645) →

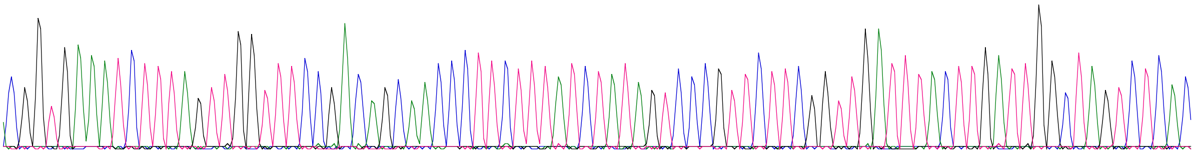

CGGTGAAATCTTTAGTTGGTTTCCGACAGCAACCCTTCTTTATCTATAGTCCCGTTCTTCGGTTGATTTACTTGATTGGCTAGTCTCAC

630 640 650 660 670 680 690 700 710

ACAAGTCGATCAATAAATCTGTTGAAGATGTCACCATCAGTCGATCCGATCAACTAGTAATCGTAATAGAAGCAAATGAACTGAGATCT

c5c2(1>1000) → ACAAGTCGATCAATAAATCTGTTGAAGATGTCACCATCAGTCGATCCGATCAACTAGTAATCGTAATAGAAGCAAATGAACTGAGATCT
c5c2.ab1(21>645) →

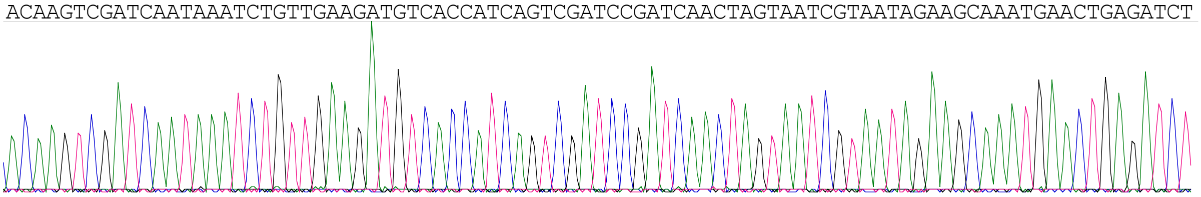

ACAAGTCGATCAATAAATCTGTTGAAGATGTCACCATCAGTCGATCCGATCAACTAGTAATCGTAATAGAAGCAAATGAACTGAGATCT

720 730 740 750 760 770 780 790 800

AACTCCTTTACTCTCAAGCTTGTGTTGTTCAATCAAGCTAAATGAACAGATTGGAAAAAGGAAGCCACATCACTGAGCGGGACTGAACC

c5c2(1>1000) → AACTCCTTTACTCTCAAGCTTGTGTTGTTCAATCAAGCTAAATGAACAGATTGGAAAAAGGAAGCCACATCACTGAGCGGGACTGAACC

c5c2.ab1(21>645) →

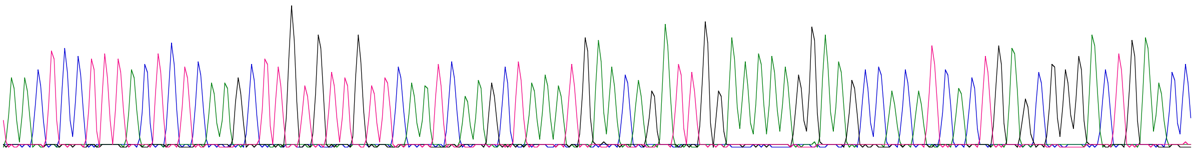

AACTCCTTTACTCTCAAGCTTGTGTTGTTCAATCAAGCTAAATGAACAGATTGGAAAAAGGAAGCCACATCACTGAGCGGGACTGAACC

810 820 830 840 850 860 870 880 890

CAATCAATCTGTCAACCTAGGGCGGACTGAATCACAGACAGGGATCACCGGGCCGGGCACTTCTGGAAGCTGAGAACGTCCATTCCCTG

c5c2(1>1000) → CAATCAATCTGTCAACCTAGGGCGGACTGAATCACAGACAGGGATCACCGGGCCGGGCACTTCTGGAAGCTGAGAACGTCCATTCCCTG

c5c2.ab1(21>645) →

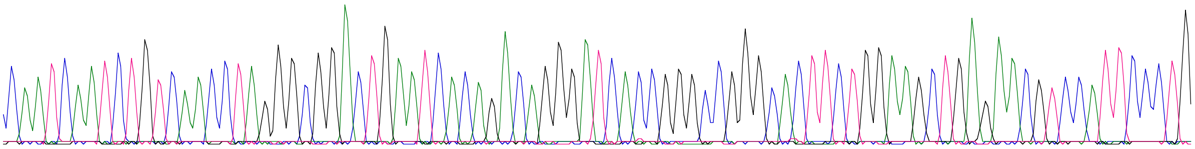

CAATCAATCTGTCAACCTAGGGCGGACTGAATCACAGACAGGGATCACCGGGCCGGGCACTTCTGGAAGCTGAGAACGTCCATTCCCTG

900 910 920 930 940 950 960 970

GCTATCTTTTTTATCTGTTTACGCGCATTCTATGATTCTGGTAACCAATCTCATAGGGGAGAAGTCATCTGTCACACGCGGGTGTTAGG

c5c2(1>1000) → GCTATCTTTTTTATCTGTTTACGCGCATTCTATGATTCTGGTAACCAATCTCATAGGGGAGAAGTCATCTGTCACACGCGGGTGTTAGG

c5c2.ab1(21>645) →

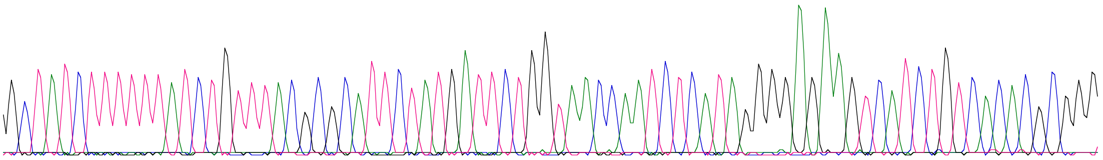

GCTATCTTTTTTATCTGTTTACGCGCATTCTATGATTCTGGTAACCAATCTCATAGGGGAGAAGTCATCTGTCACACGCGGG

980 990 1000

GCGAACTCCGGACCTCAAACA

c5c2(1>1000) → GCGAACTCCGGACCTCAAACA

10 20 30 40 50 60 70 80

AGCAGCACTTCCACTGCTAGGACTCTTCACACAAGACAAGACGGTCGGGCTGACCCAAGGGATCAATCAACTGGGACTTCTCCCTCTCG

c5c3(1>1000) AGCAGCACTTCCACTGCTAGGACTCTTCACACAAGACAAGACGGTCGGGCTGACCCAAGGGATCAATCAACTGGGACTTCTCCCTCTCG

90 100 110 120 130 140 150 160 170

CTCTCTTCTCATTGGATTCTTCTCATTCCACTTTGAAATAGGAACAACAACAACAAGAAGATGGCGCACGCGGGTGCTAGTATAAATGC

c5c3(1>1000) CTCTCTTCTCATTGGATTCTTCTCATTCCACTTTGAAATAGGAACAACAACAACAAGAAGATGGCGCACGCGGGTGCTAGTATAAATGC
c5c3.ab1(36>649)

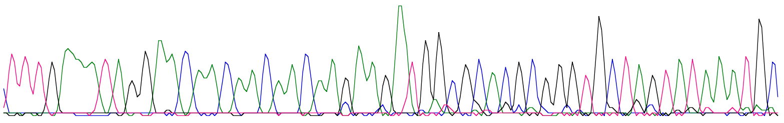

180 190 200 210 220 230 240 250 260

TGCACCTCTAGTGGTTTCCTCCGACGAAGCTGCCTTTGTTTTTGATGCCGAAGCTGTCGATGTTGTTTCTTTGGTGACCTTTCCCCGTT

c5c3(1>1000) TGCACCTCTAGTGGTTTCCTCCGACGAAGCTGCCTTTGTTTTTGATGCCGAAGCTGTCGATGTTGTTTCTTTGGTGACCTTTCCCCGTT
c5c3.ab1(36>649)

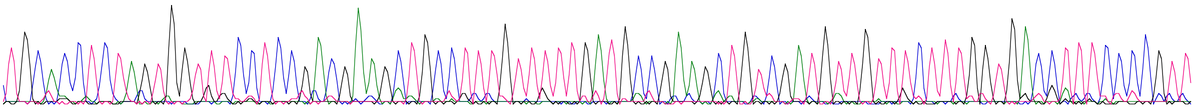

270 280 290 300 310 320 330 340 350

CCACAGTGGGCTAGCCTCAATAGAAATTCTCTTGCCACTTTCTGTAGTGAGAAAAGCTCTTTGATAGGCTTTCACGCCAATTCAGTACT

c5c3(1>1000) CCACAGTGGGCTAGCCTCAATAGAAATTCTCTTGCCACTTTCTGTAGTGAGAAAAGCTCTTTGATAGGCTTTCACGCCAATTCAGTACT
c5c3.ab1(36>649)

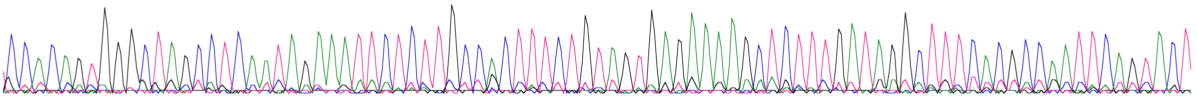

360 370 380 390 400 410 420 430 440

CGTAATCAACGACTTCGATAAAAGGGCCGAAACCTGGCCTAGGAGCAAAAAACCTAAACACAGAAGAAGCGCCTTCAAGCTTAGCCCGC

c5c3(1>1000) CGTAATCAACGACTTCGATAAAAGGGCCGAAACCTGGCCTAGGAGCAAAAAACCTAAACACAGAAGAAGCGCCTTCAAGCTTAGCCCGC
c5c3.ab1(36>649)

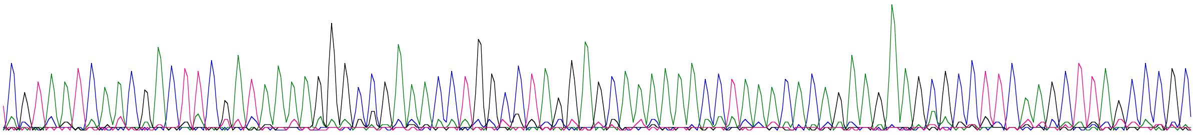

450 460 470 480 490 500 510 520 530

TCACTCCTACCCGTTCAGTCCTAGCTGTCTGTCCTTGCTTGGCTTGCCTGCCTGGTTCTTCCTGTTGATTCGGGAGACCTCAGAACTAC

c5c3(1>1000) TCACTCCTACCCGTTCAGTCCTAGCTGTCTGTCCTTGCTTGGCTTGCCTGCCTGGTTCTTCCTGTTGATTCGGGAGACCTCAGAACTAC
c5c3.ab1(36>649)

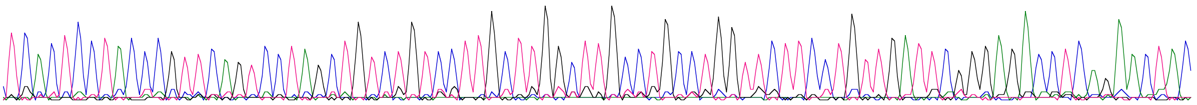

```
                    540       550       560       570       580       590       600       610       620
                    AGACCCGGCTTATTAGCGTGCCTATCTATTTTTTTACTTCGAAGAATTCGTTCGAGAGGCTTCCGTGCCTATCTAGTTAGTTAGTCAGT
c5c3(1>1000)      → AGACCCGGCTTATTAGCGTGCCTATCTATTTTTTTACTTCGAAGAATTCGTTCGAGAGGCTTCCGTGCCTATCTAGTTAGTTAGTCAGT
c5c3.ab1(36>649)  →
```

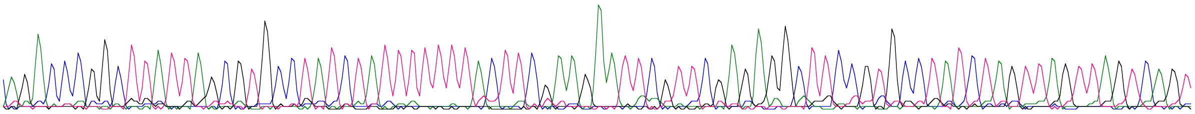

```
                    AGACCCGGCTTATTAGCGTGCCTATCTATTTTTTTACTTCGAAGAATTCGTTCGAGAGGCTTCCGTGCCTATCTAGTTAGTTAGTCAGT

                      630       640       650       660       670       680       690       700       710
                    CCTTTCCCGGTTCCTAGACCGCGGGCCGGGTCACTCCACCACACCAATCAAGTTCCCAAGCGCCTGAAAGCCAACCCTGGTCACTCCGA
c5c3(1>1000)      → CCTTTCCCGGTTCCTAGACCGCGGGCCGGGTCACTCCACCACACCAATCAAGTTCCCAAGCGCCTGAAAGCCAACCCTGGTCACTCCGA
c5c3.ab1(36>649)  →
```

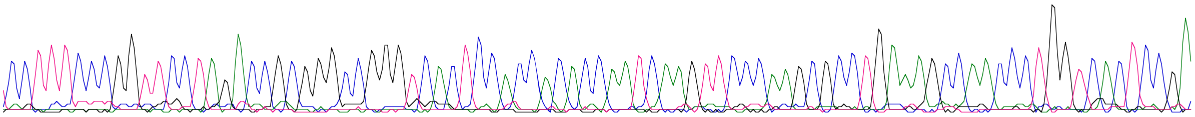

```
                    CCTTTCCCGGTTCCTAGACCGCGGGCCGGGTCACTCCACCACACCAATCAAGTTCCCAAGCGCCTGAAAGCCAACCCTGGTCACTCCGA

                       720       730       740       750       760       770       780       790       800
                    CCCAGACCCCGCCTCTACAAGCCTTTCTCATTTCACATATACGGAATTTCGGCTATGACCAATGCCCCACTTGTAGAAGTTACTTCTTA
c5c3(1>1000)      → CCCAGACCCCGCCTCTACAAGCCTTTCTCATTTCACATATACGGAATTTCGGCTATGACCAATGCCCCACTTGTAGAAGTTACTTCTTA
c5c3.ab1(36>649)  →
```

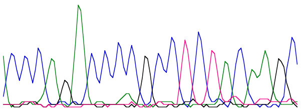

```
                    CCCAGACCCCGCCTCTACAAGC

                        810       820       830       840       850       860       870       880       890
                    CTATGAGAAACGTCAAACAATACATTAGGAAAAAGATTGAAAGCCGTCATCCTGGATCTGGTCGAAAGTGATCCTTTCTTTGTATTCCC
c5c3(1>1000)      → CTATGAGAAACGTCAAACAATACATTAGGAAAAAGATTGAAAGCCGTCATCCTGGATCTGGTCGAAAGTGATCCTTTCTTTGTATTCCC

                         900       910       920       930       940       950       960       970
                    TGGGGAGTTCCCCACTCCGCTAACGCTATGACGTAGAAGTGACTCCTGCACTATACGGCTACGCTCTAACGCTCGACCTTAACCAGTTC
c5c3(1>1000)      → TGGGGAGTTCCCCACTCCGCTAACGCTATGACGTAGAAGTGACTCCTGCACTATACGGCTACGCTCTAACGCTCGACCTTAACCAGTTC

                   980       990       1000
                    AATGTAACGTGAAAACGCCGT
c5c3(1>1000)      → AATGTAACGTGAAAACGCCGT
```

10 20 30 40 50 60 70 80

CCCCCAGGTTCCAGACCCAGGAGTCAACTTTCCCGTATGAGCATTCGGTACATGTATCAGTCCGTGGAAGAGTGAAAGGGTCACCACTA

c6c5(1>1000) → CCCCCAGGTTCCAGACCCAGGAGTCAACTTTCCCGTATGAGCATTCGGTACATGTATCAGTCCGTGGAAGAGTGAAAGGGTCACCACTA
c6c5.ab1(24>610) →

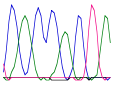

CACCACTA

90 100 110 120 130 140 150 160 170

CTGAGGATCTCCCCCCTAATCTTAGATAGGTCGTCTGAGGGTTCGCCGCGGTTCATTGCTGTGCTTACACACTAGGCTACCCTTCTCCG

c6c5(1>1000) → CTGAGGATCTCCCCCCTAATCTTAGATAGGTCGTCTGAGGGTTCGCCGCGGTTCATTGCTGTGCTTACACACTAGGCTACCCTTCTCCG
c6c5.ab1(24>610) →

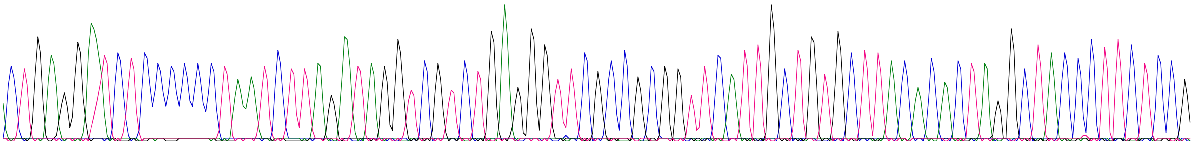

CTGAGGATCTCCCCCCTAATCTTAGATAGGTCGTCTGAGGGTTCGCCGCGGTTCATTGCTGTGCTTACACACTAGGCTACCCTTCTCCG

180 190 200 210 220 230 240 250 260

AAAGCTCCGCGGGACCACCTACCACTAGTCTTCGGCCGGAGGGGTTTATTGCACAAAAACGCCGGGACGCAGGCTCCCGAAGAGGGAAG

c6c5(1>1000) → AAAGCTCCGCGGGACCACCTACCACTAGTCTTCGGCCGGAGGGGTTTATTGCACAAAAACGCCGGGACGCAGGCTCCCGAAGAGGGAAG
c6c5.ab1(24>610) →

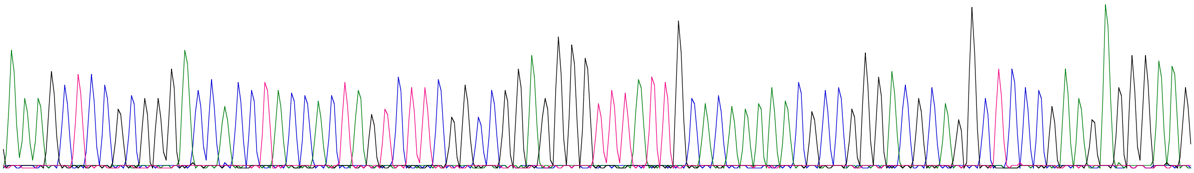

AAAGCTCCGCGGGACCACCTACCACTAGTCTTCGGCCGGAGGGGTTTATTGCACAAAAACGCCGGGACGCAGGCTCCCGAAGAGGGAAG

270 280 290 300 310 320 330 340 350

CCCAACGAATGTCAGATGCAAAGCCCCGCACCTCATTAAGATCATATTGGCATACTCTCCCAAAAAAGAAAGAGCAGACCCCATTGAAG

c6c5(1>1000) → CCCAACGAATGTCAGATGCAAAGCCCCGCACCTCATTAAGATCATATTGGCATACTCTCCCAAAAAAGAAAGAGCAGACCCCATTGAAG
c6c5.ab1(24>610) →

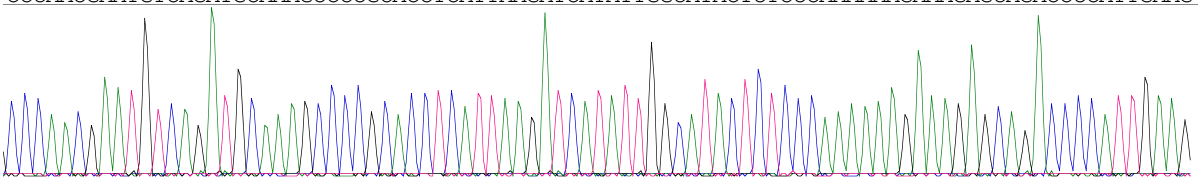

CCCAACGAATGTCAGATGCAAAGCCCCGCACCTCATTAAGATCATATTGGCATACTCTCCCAAAAAAGAAAGAGCAGACCCCATTGAAG

360 370 380 390 400 410 420 430 440

ACGGGAGTGAGGTACAACAAGGCCATTTCTGTCCGCCGCCCTTCTCACGGAGCCGTACGTGGACGTTACCGCTCATACAGCTCCCAGCC

c6c5(1>1000) → ACGGGAGTGAGGTACAACAAGGCCATTTCTGTCCGCCGCCCTTCTCACGGAGCCGTACGTGGACGTTACCGCTCATACAGCTCCCAGCC
c6c5.ab1(24>610) →

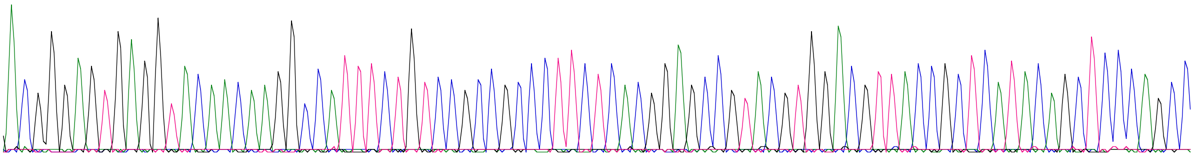

ACGGGAGTGAGGTACAACAAGGCCATTTCTGTCCGCCGCCCTTCTCACGGAGCCGTACGTGGACGTTACCGCTCATACAGCTCCCAGCC

450 460 470 480 490 500 510 520 530

AGCAAGCAGTTAGCCTTCCTCTACAAGGAATGGAAGTGTGGATGAATCGACATCAAATAGAGGAATTTTGTTTTTCTTTTCAGCAATAA

c6c5(1>1000) → AGCAAGCAGTTAGCCTTCCTCTACAAGGAATGGAAGTGTGGATGAATCGACATCAAATAGAGGAATTTTGTTTTTCTTTTCAGCAATAA
c6c5.ab1(24>610) →

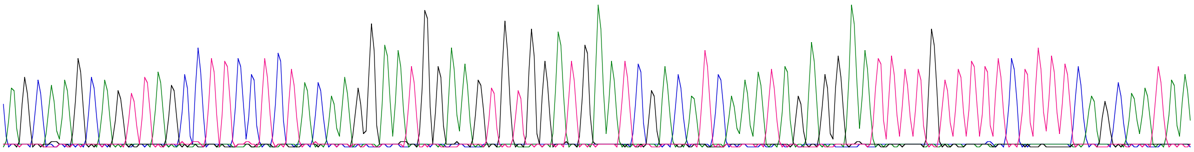

AGCAAGCAGTTAGCCTTCCTCTACAAGGAATGGAAGTGTGGATGAATCGACATCAAATAGAGGAATTTTGTTTTTCTTTTCAGCAATAA

540 550 560 570 580 590 600 610 620

CATGATAAGAGCATCCCTTTCACAAAAACCTACAGACCCCCCTATCCTGCCACTTCAGCACCTTGTGATCTTTGAGAAGATCATTACGA

c6c5(1>1000) → CATGATAAGAGCATCCCTTTCACAAAAACCTACAGACCCCCCTATCCTGCCACTTCAGCACCTTGTGATCTTTGAGAAGATCATTACGA
c6c5.ab1(24>610) →

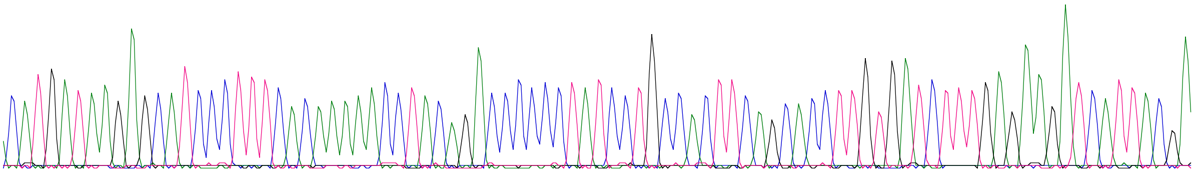

CATGATAAGAGCATCCCTTTCACAAAAACCTACAGACCCCCCTATCCTGCCACTTCAGCACCTTGTGATCTTTGAGAAGATCATTACGA

630 640 650 660 670 680 690 700 710

GCCCTCTCCGTTAATGTTTTTGTAGATTTCGAAAATTCCATGGTGGATCAAGACGAGAATGAATCCGGGAATCCAGGACATACTCATCC

c6c5(1>1000) → GCCCTCTCCGTTAATGTTTTTGTAGATTTCGAAAATTCCATGGTGGATCAAGACGAGAATGAATCCGGGAATCCAGGACATACTCATCC
c6c5.ab1(24>610) →

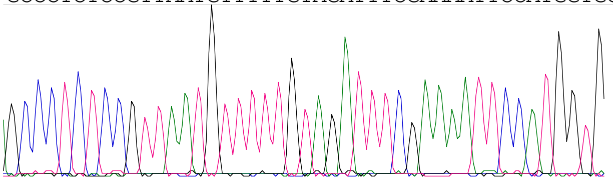

GCCCTCTCCGTTAATGTTTTTGTAGATTTCGAAAATTCCATGGTG

720 730 740 750 760 770 780 790 800

TGGAGCTTCTGCTCTCCTCTGGGGAGGGGTTTTTTATAGAGAGAGAGGTGAGTCGAGGGTAGCCCCCCGCTTGACCGATTTCTCGGAGT

c6c5(1>1000) → TGGAGCTTCTGCTCTCCTCTGGGGAGGGGTTTTTTATAGAGAGAGAGGTGAGTCGAGGGTAGCCCCCCGCTTGACCGATTTCTCGGAGT

810 820 830 840 850 860 870 880 890

CGGAGGAAGATCTCTCATCTGATGACCCTGAACAAGAAGGAGCTGCTCTCGATGTGAGATCAGCAGCAAAAGAAAGAAGAGGAATTGGA

c6c5(1>1000) → CGGAGGAAGATCTCTCATCTGATGACCCTGAACAAGAAGGAGCTGCTCTCGATGTGAGATCAGCAGCAAAAGAAAGAAGAGGAATTGGA

900 910 920 930 940 950 960 970

TGCCCCAGAGCAGCAGCCCCCGGATAAGCCTTAAAAAAAAACACACACAACGGACCGGACACAAACAAAAGAAAGAAGAAAAGGGCCAT

c6c5(1>1000) → TGCCCCAGAGCAGCAGCCCCCGGATAAGCCTTAAAAAAAAACACACACAACGGACCGGACACAAACAAAAGAAAGAAGAAAAGGGCCAT

980 990 1000

GATCCTATGTTCGTTTTCGCC

c6c5(1>1000) → GATCCTATGTTCGTTTTCGCC

10 20 30 40 50 60 70 80

AGCCGGCTCTAGTCTGAGTACATATTGTTTACCAGAAGAGGTTGTTGGAGAGGATAGGCAGATCCAGATAAATGAAAGGGGCTTGTCCT

c7c5(1>1000) → AGCCGGCTCTAGTCTGAGTACATATTGTTTACCAGAAGAGGTTGTTGGAGAGGATAGGCAGATCCAGATAAATGAAAGGGGCTTGTCCT

90 100 110 120 130 140 150 160 170

CACTTACTTGAGCAAGGAATGAAAGACTCGCTTGCTTAGCGCAGGACGGAACAAGAAAGAAAAAGAGAAAGACCTATTATGAAACTAAG

c7c5(1>1000) → CACTTACTTGAGCAAGGAATGAAAGACTCGCTTGCTTAGCGCAGGACGGAACAAGAAAGAAAAAGAGAAAGACCTATTATGAAACTAAG
c7c5.ab1(16>651) →

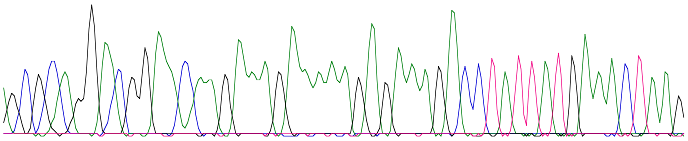

GCGCAGGACGGAACAAGAAAGAAAAAGAGAAAGACCTATTATGAAACTAAG

180 190 200 210 220 230 240 250 260

AGAAAAGCAAGTAGCACAATCCCCTCACCTAGGGGAAGACCCCTTCTTATTCTTGTACGTATACTAGATCTGTCTGGATTGGATCTACT

c7c5(1>1000) → AGAAAAGCAAGTAGCACAATCCCCTCACCTAGGGGAAGACCCCTTCTTATTCTTGTACGTATACTAGATCTGTCTGGATTGGATCTACT
c7c5.ab1(16>651) →

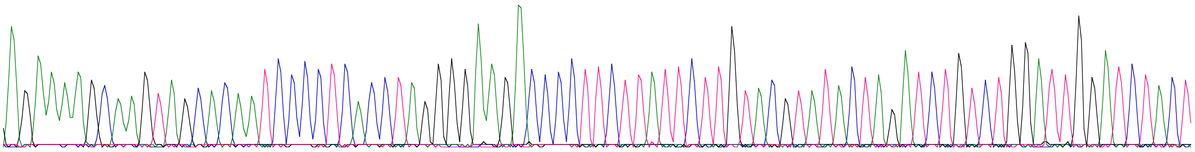

AGAAAAGCAAGTAGCACAATCCCCTCACCTAGGGGAAGACCCCTTCTTATTCTTGTACGTATACTAGATCTGTCTGGATTGGATCTACT

270 280 290 300 310 320 330 340 350

AGAAGAGTAGAAGAGGAATTATTCTATTTATGCCTCTATTTATGTTTTATGCCTTTAGTGCAACAAATAGATTTGAGTTTTGTAACCTA

c7c5(1>1000) → AGAAGAGTAGAAGAGGAATTATTCTATTTATGCCTCTATTTATGTTTTATGCCTTTAGTGCAACAAATAGATTTGAGTTTTGTAACCTA
c7c5.ab1(16>651) →

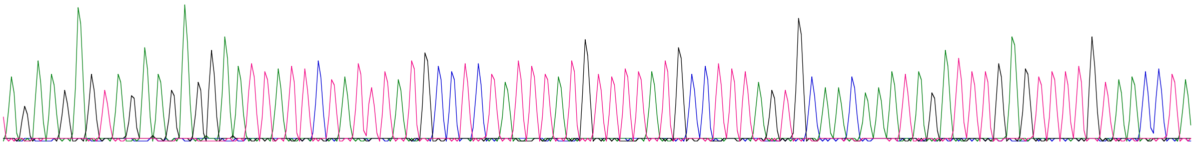

AGAAGAGTAGAAGAGGAATTATTCTATTTATGCCTCTATTTATGTTTTATGCCTTTAGTGCAACAAATAGATTTGAGTTTTGTAACCTA

360 370 380 390 400 410 420 430 440

ATAGGGTTGTGTTCATCCTGCGTACTATCTGCTATACGCATTAATTGGTGCATCTCCTGTATAGGAATGACCCTCTAGTAGCTGCACAT

c7c5(1>1000) → ATAGGGTTGTGTTCATCCTGCGTACTATCTGCTATACGCATTAATTGGTGCATCTCCTGTATAGGAATGACCCTCTAGTAGCTGCACAT
c7c5.ab1(16>651) →

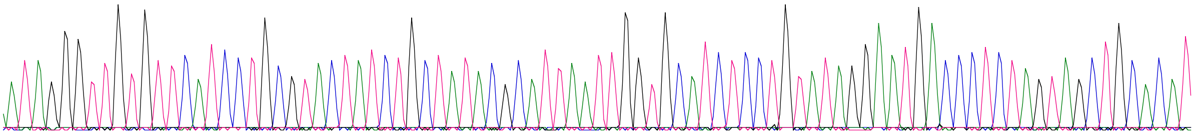

ATAGGGTTGTGTTCATCCTGCGTACTATCTGCTATACGCATTAATTGGTGCATCTCCTGTATAGGAATGACCCTCTAGTAGCTGCACAT

450 460 470 480 490 500 510 520 530

CGTAGTCAACAACTGATTTAGGAATAGAATAGGTTCTTCCCTCTCTCTCCTATCTAATAGAGGAATTTTGTTTTTCTTTTCAGCAATAA

c7c5(1>1000) → CGTAGTCAACAACTGATTTAGGAATAGAATAGGTTCTTCCCTCTCTCTCCTATCTAATAGAGGAATTTTGTTTTTCTTTTCAGCAATAA
c7c5.ab1(16>651) →

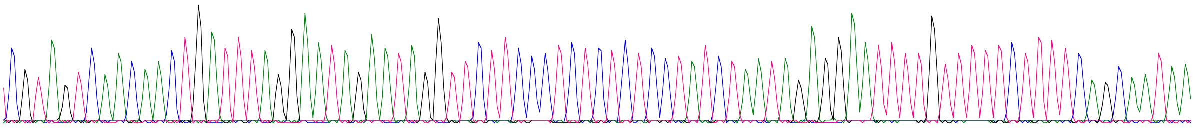

CGTAGTCAACAACTGATTTAGGAATAGAATAGGTTCTTCCCTCTCTCTCCTATCTAATAGAGGAATTTTGTTTTTCTTTTCAGCAATAA

```
            540       550       560       570       580       590       600       610       620
            CATGATAAGAGCATCCCTTTCACAAAAACCTACAGACCCCCCTATCCTGCCACTTCAGCACCTTGTGATCTTTGAGAAGATCATTACGA
c7c5(1>1000)     →  CATGATAAGAGCATCCCTTTCACAAAAACCTACAGACCCCCCTATCCTGCCACTTCAGCACCTTGTGATCTTTGAGAAGATCATTACGA
c7c5.ab1(16>651) →
```

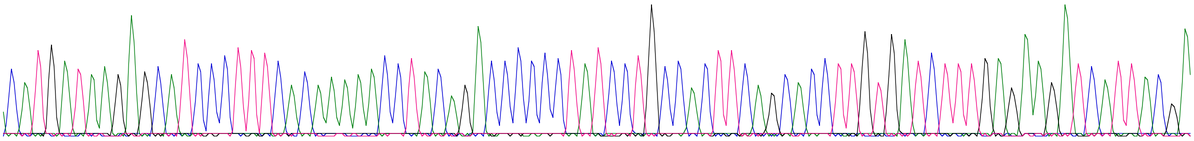

```
CATGATAAGAGCATCCCTTTCACAAAAACCTACAGACCCCCCTATCCTGCCACTTCAGCACCTTGTGATCTTTGAGAAGATCATTACGA

            630       640       650       660       670       680       690       700       710
            GCCCTCTCCGTTAATGTTTTTGTAGATTTCGAAAATTCCATGGTGGATCAAGACGAGAATGAATCCGGGAATCCAGGACATACTCATCC
c7c5(1>1000)     →  GCCCTCTCCGTTAATGTTTTTGTAGATTTCGAAAATTCCATGGTGGATCAAGACGAGAATGAATCCGGGAATCCAGGACATACTCATCC
c7c5.ab1(16>651) →
```

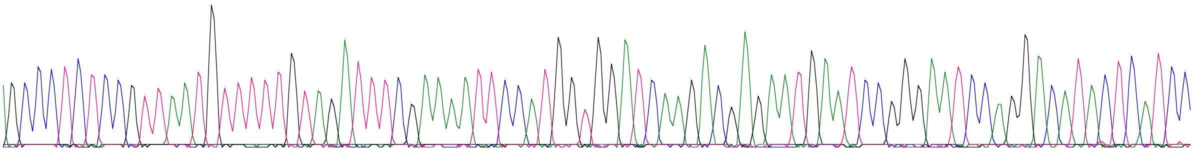

```
GCCCTCTCCGTTAATGTTTTTGTAGATTTCGAAAATTCCATGGTGGATCAAGACGAGAATGAATCCGGGAATCCAGGACATACTCATCC

            720       730       740       750       760       770       780       790       800
            TGGAGCTTCTGCTCTCCTCTGGGGAGGGGTTTTTTATAGAGAGAGAGGTGAGTCGAGGGTAGCCCCCCGCTTGACCGATTTCTCGGAGT
c7c5(1>1000)     →  TGGAGCTTCTGCTCTCCTCTGGGGAGGGGTTTTTTATAGAGAGAGAGGTGAGTCGAGGGTAGCCCCCCGCTTGACCGATTTCTCGGAGT
c7c5.ab1(16>651) →
```

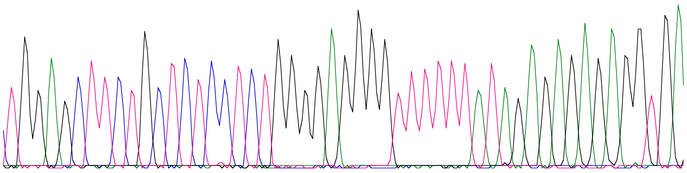

```
TGGAGCTTCTGCTCTCCTCTGGGGAGGGGTTTTTTATAGAGAGAGAGGTGA

            810       820       830       840       850       860       870       880       890
            CGGAGGAAGATCTCTCATCTGATGACCCTGAACAAGAAGGAGCTGCTCTCGATGTGAGATCAGCAGCAAAAGAAAGAAGAGGAATTGGA
c7c5(1>1000)     →  CGGAGGAAGATCTCTCATCTGATGACCCTGAACAAGAAGGAGCTGCTCTCGATGTGAGATCAGCAGCAAAAGAAAGAAGAGGAATTGGA

            900       910       920       930       940       950       960       970
            TGCCCCAGAGCAGCAGCCCCCGGATAAGCCTTAAAAAAAAACACACACAACGGACCGGACACAAACAAAAGAAAGAAGAAAAGGGCCAT
c7c5(1>1000)     →  TGCCCCAGAGCAGCAGCCCCCGGATAAGCCTTAAAAAAAAACACACACAACGGACCGGACACAAACAAAAGAAAGAAGAAAAGGGCCAT

            980       990       1000
            GATCCTATGTTCGTTTTCGCC
c7c5(1>1000)     →  GATCCTATGTTCGTTTTCGCC
```

10 20 30 40 50 60 70 80

CCATTGAGTATAACAGAGCAAATGATGGTATAGCAATGAACATCGGGATGATACTAGGAAATATGGTCCGAAGAATCTCGATAGTAGT

c8c10(1>1000) → CCATTGAGTATAACAGAGCAAATGATGGTATAGCAATGAACATCGGGATGATACTAGGAAATATGGTCCGAAGAATCTCGATAGTAGT

90 100 110 120 130 140 150 160 170

TCCATGAACAATCCTTTGCGGGATTGGATTTTTTTTATAGTGGAAATGCCATAAAGCGCGAACCAAGATCCGTGATACGAAAACCAAA

c8c10(1>1000) → TCCATGAACAATCCTTTGCGGGATTGGATTTTTTTTATAGTGGAAATGCCATAAAGCGCGAACCAAGATCCGTGATACGAAAACCAAA

180 190 200 210 220 230 240 250 260

ATCAGAATGAGGAAGAAAAAGATATCGTGATGTAAGTCTATTATTCCTTGCATCATAGGTGTTGCTGCGTCTTGAGATCCTAATTGCC

c8c10(1>1000) → ATCAGAATGAGGAAGAAAAAGATATCGTGATGTAAGTCTATTATTCCTTGCATCATAGGTGTTGCTGCGTCTTGAGATCCTAATTGCC
c8c10.ab1(28>562) →

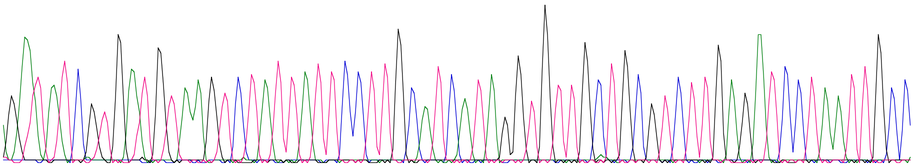

GATATCGTGATGTAAGTCTATTATTCCTTGCATCATAGGTGTTGCTGCGTCTTGAGATCCTAATTGCC

270 280 290 300 310 320 330 340 350

ATGGTTCTGCTGCATCACAAGGAGCAATTGTGAGGAATCGCCATTCTAGAACAATCATTTGGTTTGGTTCATTCTTTGACTGCTCTGC

c8c10(1>1000) → ATGGTTCTGCTGCATCACAAGGAGCAATTGTGAGGAATCGCCATTCTAGAACAATCATTTGGTTTGGTTCATTCTTTGACTGCTCTGC
c8c10.ab1(28>562) →

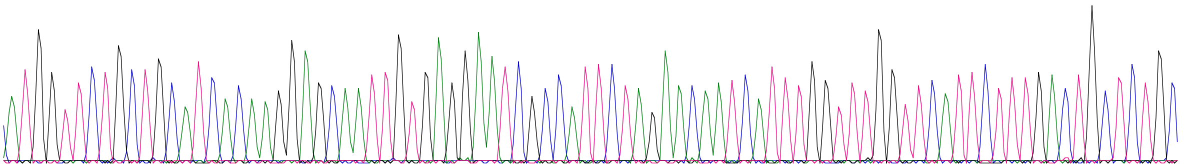

ATGGTTCTGCTGCATCACAAGGAGCAATTGTGAGGAATCGCCATTCTAGAACAATCATTTGGTTTGGTTCATTCTTTGACTGCTCTGC

360 370 380 390 400 410 420 430 440

TCCCCCAAAAAAAAGAGGGAGACTTGTTCTTGCTCTCCCAATTCAGGAAGATTTTAGATTGCTTGGTTGTTGACCAAGGAGAAAGAAA

c8c10(1>1000) → TCCCCCAAAAAAAAGAGGGAGACTTGTTCTTGCTCTCCCAATTCAGGAAGATTTTAGATTGCTTGGTTGTTGACCAAGGAGAAAGAAA
c8c10.ab1(28>562) →

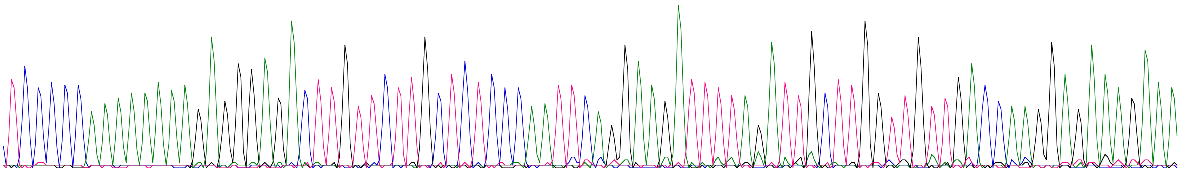

TCCCCCAAAAAAAAGAGGGAGACTTGTTCTTGCTCTCCCAATTCAGGAAGATTTTAGATTGCTTGGTTGTTGACCAAGGAGAAAGAAA

450 460 470 480 490 500 510 520

GATGCACAAACGAAAAAGGGCAATTTCATAAGGGACCATTTGAGCTGGAGCTTTCTAGATcTACTAAAGAAGTTAGTTACAACTTTTC

c8c10(1>1000) → GATGCACAAACGAAAAAGGGCAATTTCATAAGGGACCATTTGAGCTGGAGCTTTCTAGAT-TACTAAAGAAGTTAGTTACAACTTTTC
c8c10.ab1(28>562) →

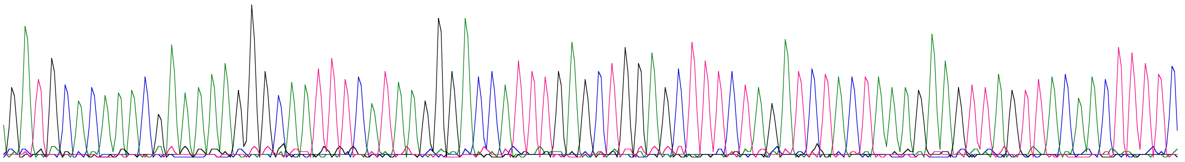

GATGCACAAACGAAAAAGGGCAATTTCATAAGGGACCATTTGAGCTGGAGCTTTCTAGATCTACTAAAGAAGTTAGTTACAACTTTTC

530 540 550 560 570 580 590 600 610

GTACACTGAACACCAATCCAATCTCGACGAAAATGGAATCAACTCGATCATTCAAGTCGGATAGCATTTATCTATGCCGACCCACATT

c8c10(1>1000) → GTACACTGAACACCAATCCAATCTCGACGAAAATGGAATCAACTCGATCATTCAAGTCGGATAGCATTTATCTATGCCGACCCACATT
c8c10.ab1(28>562) →

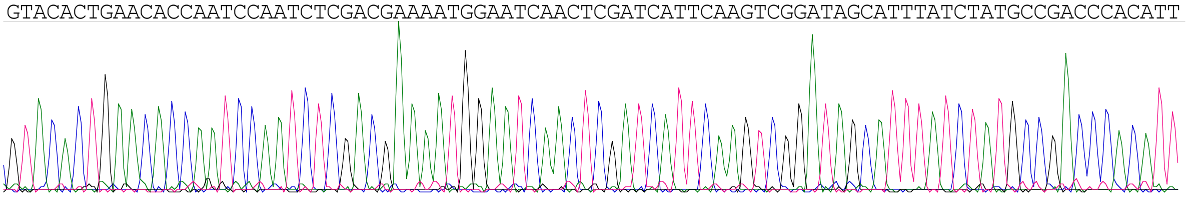

GTACACTGAACACCAATCCAATCTCGACGAAAATGGAATCAACTCGATCATTCAAGTCGGATAGCATTTATCTATGCCGACCCACATT

```
                          620       630       640       650       660       670       680       690       700
                          AACAGCTTTCTTCTCTTATAATTTCTCGAGTTACAGGGGAATCGAGTGACTTTCTCTTAAATTATATAAGTCTAAGAGAGAGTCATTC
c8c10(1>1000)        →    AACAGCTTTCTTCTCTTATAATTTCTCGAGTTACAGGGGAATCGAGTGACTTTCTCTTAAATTATATAAGTCTAAGAGAGAGTCATTC
c8c10.ab1(28>562)    →
```

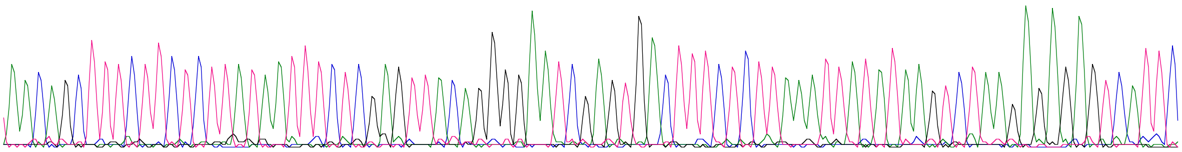

```
                          AACAGCTTTCTTCTCTTATAATTTCTCGAGTTACAGGGGAATCGAGTGACTTTCTCTTAAATTATATAAGTCTAAGAGAGAGTCATTC

                             710       720       730       740       750       760       770       780       790
                          TAGATTTAAAACTAACCCCATCAGGCAAGTGGAGTAAATCAATCAAGTGGCATACCTTGAATCTAGCCTACAATCCTTTCTTTCTTCT
c8c10(1>1000)        →    TAGATTTAAAACTAACCCCATCAGGCAAGTGGAGTAAATCAATCAAGTGGCATACCTTGAATCTAGCCTACAATCCTTTCTTTCTTCT
c8c10.ab1(28>562)    →
```

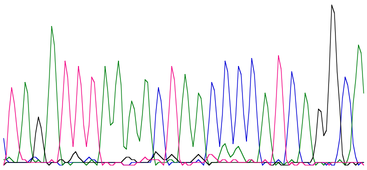

```
                          TAGATTTAAAACTAACCCCATCAGGCA

                               800       810       820       830       840       850       860       870       880
                          TTCAATTGATAGTTAGCCGGGCGTATCTTGTTCAAGAAAAAGCTCTTTCTTTTGGTGAATATCCGGATACCCGAAAGTGACAGCTAAA
c8c10(1>1000)        →    TTCAATTGATAGTTAGCCGGGCGTATCTTGTTCAAGAAAAAGCTCTTTCTTTTGGTGAATATCCGGATACCCGAAAGTGACAGCTAAA

                                 890       900       910       920       930       940       950       960
                          GATGAATGCCCAGAATCGTAGATTGTACGAGCTTCAAATCAAAACCACAATGAGAGACATTCGATCCCAGAATTAGCTCTATCCCACC
c8c10(1>1000)        →    GATGAATGCCCAGAATCGTAGATTGTACGAGCTTCAAATCAAAACCACAATGAGAGACATTCGATCCCAGAATTAGCTCTATCCCACC

                          970       980       990       1000
                          TCCAATGAGATGTGTTAAGCATATACTTACTT
c8c10(1>1000)        →    TCCAATGAGATGTGTTAAGCATATACTTACTT
```

10 20 30 40 50 60 70 80

TGAATAAAGACCTTATTCTACTCAAGACAATTAAGTCTTTTTCTGAAATTGCTGCGGGAGAGCTGGAGCTTCAACAAAAGTACAACCC

c9c10(1>1000) TGAATAAAGACCTTATTCTACTCAAGACAATTAAGTCTTTTTCTGAAATTGCTGCGGGAGAGCTGGAGCTTCAACAAAAGTACAACCC

90 100 110 120 130 140 150 160 170

AGAAAGGGTTAGCAATCCACTATGTGAATCCACAAAGGACCTACTTCATGATTCTCTCCGAAAGGGCATAGCCCAACTTCTGGAGAGA

c9c10(1>1000) AGAAAGGGTTAGCAATCCACTATGTGAATCCACAAAGGACCTACTTCATGATTCTCTCCGAAAGGGCATAGCCCAACTTCTGGAGAGA

180 190 200 210 220 230 240 250 260

CGAAACAAGCCGCTCCCCGAGAGAATGACCCACTCTGACCTAGTTGATAAATTAATTGATACGCAAGGGGTAGATCAATTTATTGTGG

c9c10(1>1000) CGAAACAAGCCGCTCCCCGAGAGAATGACCCACTCTGACCTAGTTGATAAATTAATTGATACGCAAGGGGTAGATCAATTTATTGTGG
c9c10.ab1(25>488)

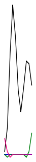

GG

270 280 290 300 310 320 330 340 350

AACCTCCGGATCTATTACGTATATACGATCTTTTAGAAGATCTTATTCTTTATGATGTCCACAGTTCGTGCTTTCAAACTGTGTTAAC

c9c10(1>1000) AACCTCCGGATCTATTACGTATATACGATCTTTTAGAAGATCTTATTCTTTATGATGTCCACAGTTCGTGCTTTCAAACTGTGTTAAC
c9c10.ab1(25>488)

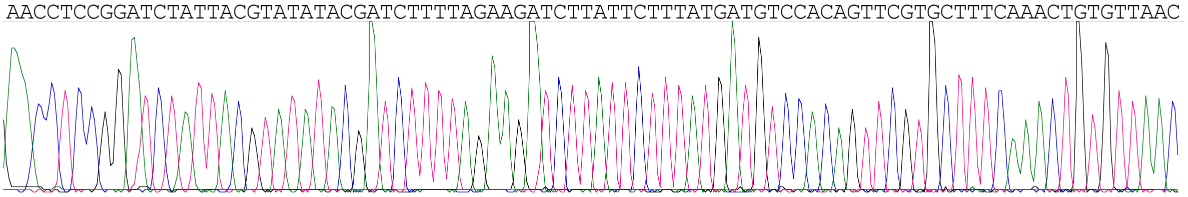

AACCTCCGGATCTATTACGTATATACGATCTTTTAGAAGATCTTATTCTTTATGATGTCCACAGTTCGTGCTTTCAAACTGTGTTAAC

360 370 380 390 400 410 420 430 440

CCTATTAAATGTCAATTAGTAATCACAAGCCTCATGCTGTCGGTATTTAAAACTTGTTAGCAGGTCCACGGCTGGCTAAGATTCTTTC

c9c10(1>1000) CCTATTAAATGTCAATTAGTAATCACAAGCCTCATGCTGTCGGTATTTAAAACTTGTTAGCAGGTCCACGGCTGGCTAAGATTCTTTC
c9c10.ab1(25>488)

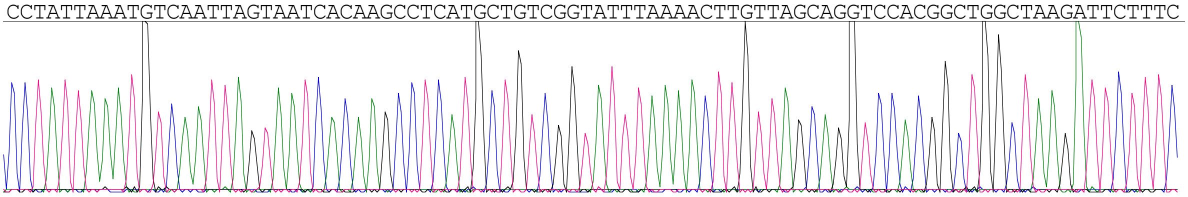

CCTATTAAATGTCAATTAGTAATCACAAGCCTCATGCTGTCGGTATTTAAAACTTGTTAGCAGGTCCACGGCTGGCTAAGATTCTTTC

450 460 470 480 490 500 510 520

TGACTTATGAAGAAAGTTACGATCTCGTTCTTTACTGACTCTTCCAGCGAGTGTTCAGTGTACTAAAGAAGTTAGTTACAACTTTTCG

c9c10(1>1000) TGACTTATGAAGAAAGTTACGATCTCGTTCTTTACTGACTCTTCCAGCGAGTGTTCAGTGTACTAAAGAAGTTAGTTACAACTTTTCG
c9c10.ab1(25>488)

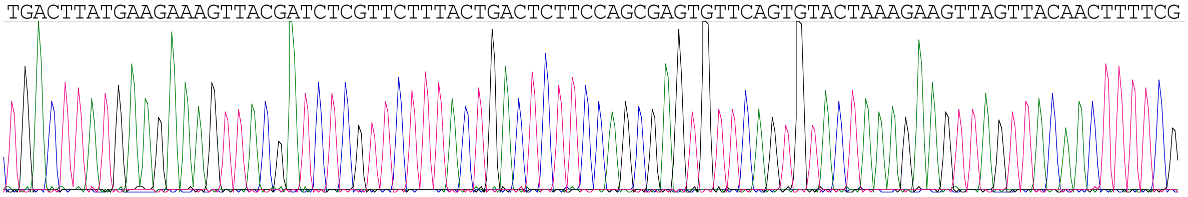

TGACTTATGAAGAAAGTTACGATCTCGTTCTTTACTGACTCTTCCAGCGAGTGTTCAGTGTACTAAAGAAGTTAGTTACAACTTTTCG

530 540 550 560 570 580 590 600 610

TACACTGAACACCAATCCAATCTCGACGAAAATGGAATCAACTCGATCATTCAAGTCGGATAGCATTTATCTATGCCGACCCACATTA

c9c10(1>1000) TACACTGAACACCAATCCAATCTCGACGAAAATGGAATCAACTCGATCATTCAAGTCGGATAGCATTTATCTATGCCGACCCACATTA
c9c10.ab1(25>488)

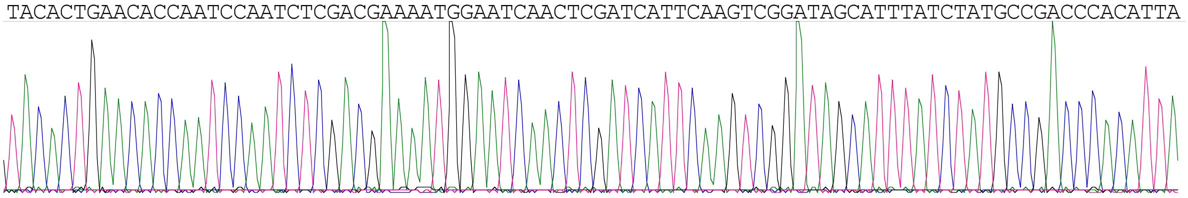

TACACTGAACACCAATCCAATCTCGACGAAAATGGAATCAACTCGATCATTCAAGTCGGATAGCATTTATCTATGCCGACCCACATTA

620 630 640 650 660 670 680 690 700

ACAGCTTTCTTCTCTTATAATTTCTCGAGTTACAGGGGAATCGAGTGACTTTCTCTTAAATTATATAAGTCTAAGAGAGAGTCATTCT

c9c10(1>1000) → ACAGCTTTCTTCTCTTATAATTTCTCGAGTTACAGGGGAATCGAGTGACTTTCTCTTAAATTATATAAGTCTAAGAGAGAGTCATTCT
c9c10.ab1(25>488) →

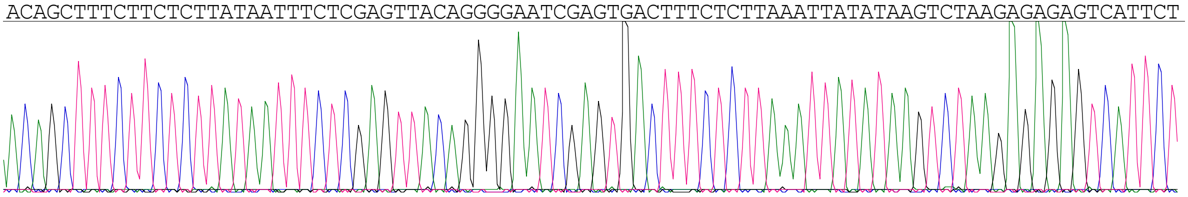

ACAGCTTTCTTCTCTTATAATTTCTCGAGTTACAGGGGAATCGAGTGACTTTCTCTTAAATTATATAAGTCTAAGAGAGAGTCATTCT

710 720 730 740 750 760 770 780 790

AGATTTAAAACTAACCCCATCAGGCAAGTGGAGTAGAATCAATCAAGTGGCATACCTTGAATCTAGCCTACAATCCTTTCTTTCTTCT

c9c10(1>1000) → AGATTTAAAACTAACCCCATCAGGCAAGTGGAGTAGAATCAATCAAGTGGCATACCTTGAATCTAGCCTACAATCCTTTCTTTCTTCT
c9c10.ab1(25>488) →

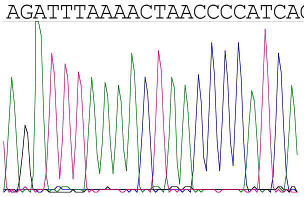

AGATTTAAAACTAACCCCATCA

800 810 820 830 840 850 860 870 880

TTCAATTGATAGTTAGCCGGGCGTATCTTGTTCAAGAAAAAGCTCTTTCTTTTGGTGAATATCCGGATACCCGAAAGTGACAGCTAAA

c9c10(1>1000) → TTCAATTGATAGTTAGCCGGGCGTATCTTGTTCAAGAAAAAGCTCTTTCTTTTGGTGAATATCCGGATACCCGAAAGTGACAGCTAAA

890 900 910 920 930 940 950 960

GATGAATGCCCAGAATCGTAGATTGTACGAGCTTCAAATCAAAACCACAATGAGAGACATTCGATCCCAGAATTAGCTCTATCCCACC

c9c10(1>1000) → GATGAATGCCCAGAATCGTAGATTGTACGAGCTTCAAATCAAAACCACAATGAGAGACATTCGATCCCAGAATTAGCTCTATCCCACC

970 980 990 1000

TCCAATGAGATGTGTTAAGCATATACTTACTT

c9c10(1>1000) → TCCAATGAGATGTGTTAAGCATATACTTACTT

10 20 30 40 50 60 70 80

CTCTAAAGGTCAGTGGTTCAAGTCAATAAGCGAGGAGGCCTTCTCACTCAAGCTCAAGCATTTACTTTTCTAGTTAGAGACCAACGTC

c9c11(1>1000) → CTCTAAAGGTCAGTGGTTCAAGTCAATAAGCGAGGAGGCCTTCTCACTCAAGCTCAAGCATTTACTTTTCTAGTTAGAGACCAACGTC

90 100 110 120 130 140 150 160 170

TTGGGGCTAACGTGGGATCCGCTCAAGGACCTACTGCTTTAGGTAAATATCTCATGCGTTCTCCGACTGGAGAGGTCATTTTTGGAGG

c9c11(1>1000) → TTGGGGCTAACGTGGGATCCGCTCAAGGACCTACTGCTTTAGGTAAATATCTCATGCGTTCTCCGACTGGAGAGGTCATTTTTGGAGG

180 190 200 210 220 230 240 250 260

AGAAACTATGCGCTTTTCAAAAAAGGTCGGAAGAACGGGTTTAAATATATATATATATATTTATATATTACCGGCTGGCTGCTTTTTA

c9c11(1>1000) → AGAAACTATGCGCTTTTCAAAAAAGGTCGGAAGAACGGGTTTAAATATATATATATATATTTATATATTACCGGCTGGCTGCTTTTTA
c9c11.ab1(32>590) →

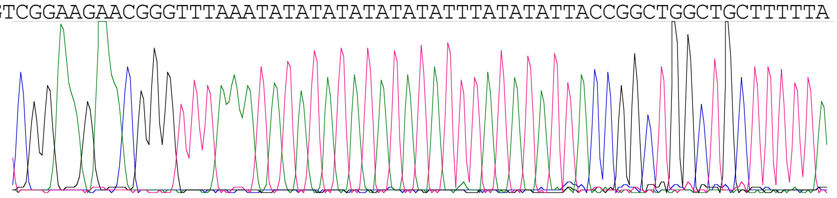

CGGAAGAACGGGTTTAAATATATATATATATATTTATATATTACCGGCTGGCTGCTTTTTA

270 280 290 300 310 320 330 340 350

TGCAAAAAAGACAAAACGGGATTTGATTTCCACTCATAAGGAAGGAAGGTTAGATATCTTTGACAGGGTTGTATTTTTGGTTGAAATG

c9c11(1>1000) → TGCAAAAAAGACAAAACGGGATTTGATTTCCACTCATAAGGAAGGAAGGTTAGATATCTTTGACAGGGTTGTATTTTTGGTTGAAATG
c9c11.ab1(32>590) →

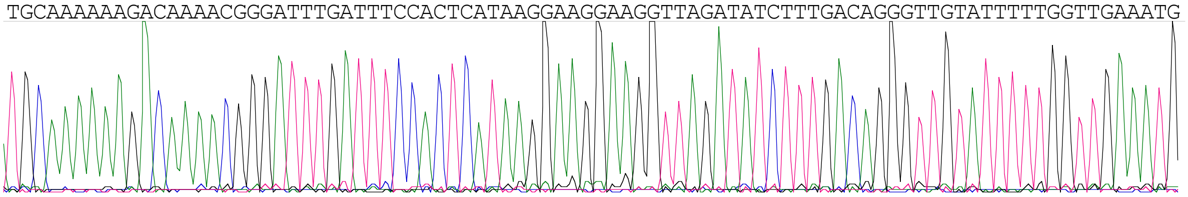

TGCAAAAAAGACAAAACGGGATTTGATTTCCACTCATAAGGAAGGAAGGTTAGATATCTTTGACAGGGTTGTATTTTTGGTTGAAATG

360 370 380 390 400 410 420 430 440

GGATGGTGTTCAAACTCCCGAAATGCAGTCTAGACAGCGGGCCCTCCCCTTTCTGACTGGACGGACTTGGGGAAGGGTCAATCCCCCC

c9c11(1>1000) → GGATGGTGTTCAAACTCCCGAAATGCAGTCTAGACAGCGGGCCCTCCCCTTTCTGACTGGACGGACTTGGGGAAGGGTCAATCCCCCC
c9c11.ab1(32>590) →

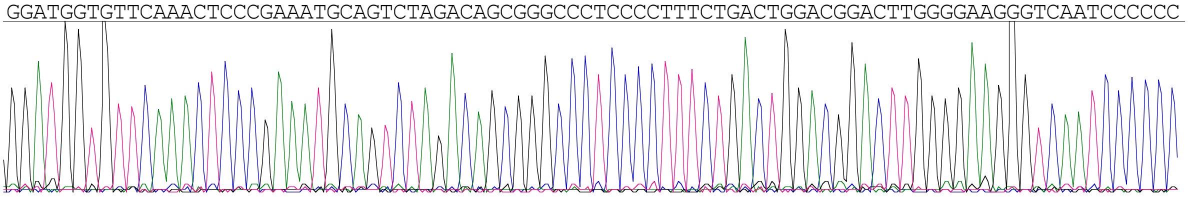

GGATGGTGTTCAAACTCCCGAAATGCAGTCTAGACAGCGGGCCCTCCCCTTTCTGACTGGACGGACTTGGGGAAGGGTCAATCCCCCC

450 460 470 480 490 500 510 520

CGGAGCATGCTTCACAGCCACTCATTCGTCCTGACCAAATAGTAGAATCTATCTCTCAGCGATTCGCGGAGAAAAGAATTGCTGAGAG

c9c11(1>1000) → CGGAGCATGCTTCACAGCCACTCATTCGTCCTGACCAAATAGTAGAATCTATCTCTCAGCGATTCGCGGAGAAAAGAATTGCTGAGAG
c9c11.ab1(32>590) →

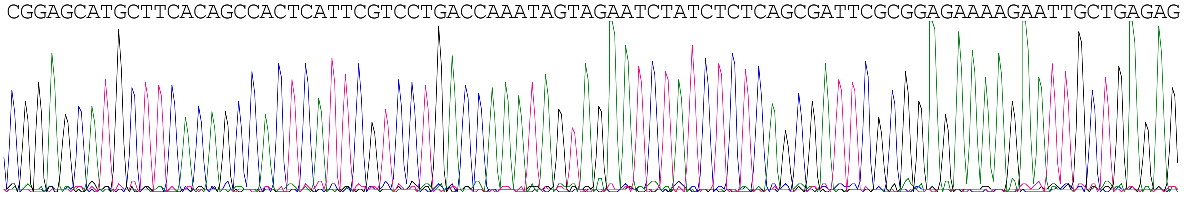

CGGAGCATGCTTCACAGCCACTCATTCGTCCTGACCAAATAGTAGAATCTATCTCTCAGCGATTCGCGGAGAAAAGAATTGCTGAGAG

530 540 550 560 570 580 590 600 610

ATAGATTCTGGTCAGGACCCTCTAATTTAGGGATTAGTCAGAAAGCAAGTCTCTCTCACGAAACAACAGAGAATTTTTTATCCTGTCT

c9c11(1>1000) → ATAGATTCTGGTCAGGACCCTCTAATTTAGGGATTAGTCAGAAAGCAAGTCTCTCTCACGAAACAACAGAGAATTTTTTATCCTGTCT
c9c11.ab1(32>590) →

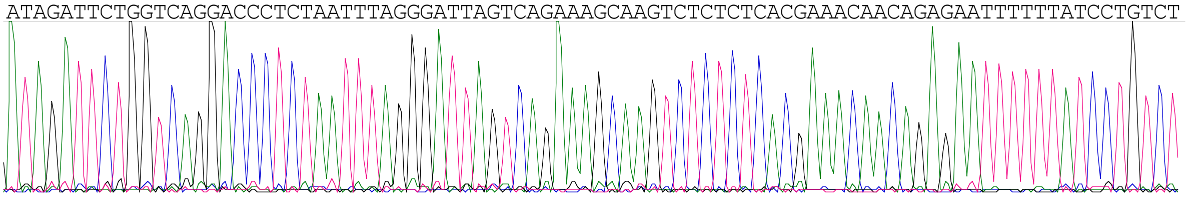

ATAGATTCTGGTCAGGACCCTCTAATTTAGGGATTAGTCAGAAAGCAAGTCTCTCTCACGAAACAACAGAGAATTTTTTATCCTGTCT

620 630 640 650 660 670 680 690 700

AACCTCTGCTACTGCAGGTCTGGTATGATAGAATCCCTAGGTTATCAGTTCACGAAGTGCAACGAGTTCAGTAGGGAACGGCCTGCGA

c9c11(1>1000) AACCTCTGCTACTGCAGGTCTGGTATGATAGAATCCCTAGGTTATCAGTTCACGAAGTGCAACGAGTTCAGTAGGGAACGGCCTGCGA
c9c11.ab1(32>590)

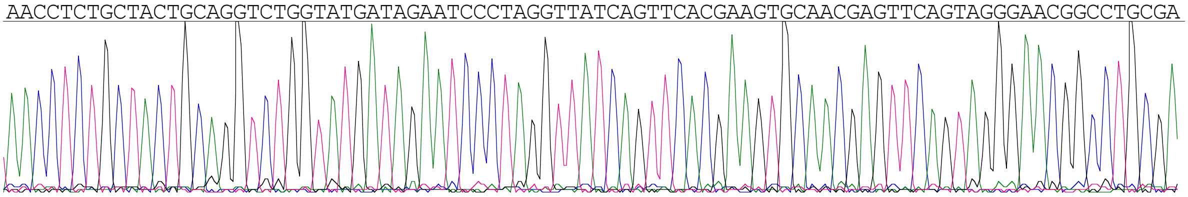

AACCTCTGCTACTGCAGGTCTGGTATGATAGAATCCCTAGGTTATCAGTTCACGAAGTGCAACGAGTTCAGTAGGGAACGGCCTGCGA

710 720 730 740 750 760 770 780 790

GTCATTTTTGGGAAGAGAGCTTAAATAGCTATTTTGAACTATACCATTTAGTAAACAGTCACAAAGGGTTTGCTTTAATTTCATACCT

c9c11(1>1000) GTCATTTTTGGGAAGAGAGCTTAAATAGCTATTTTGAACTATACCATTTAGTAAACAGTCACAAAGGGTTTGCTTTAATTTCATACCT
c9c11.ab1(32>590)

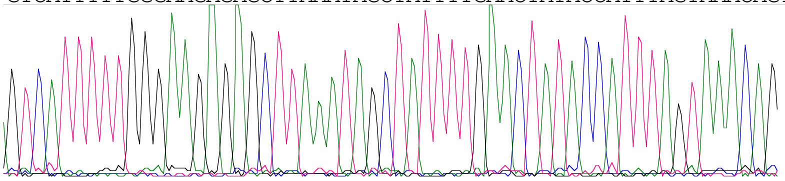

GTCATTTTTGGGAAGAGAGCTTAAATAGCTATTTTGAACTATACCATTTAGTAAACAG

800 810 820 830 840 850 860 870 880

TTCAATCATCAGTCATAATTAGGAAGAGAAGACAGAAAAGCAGCCGGTAGAATAGCTCACTCCATTGGGGAAAGAAAAAAAGTTAGTG

c9c11(1>1000) TTCAATCATCAGTCATAATTAGGAAGAGAAGACAGAAAAGCAGCCGGTAGAATAGCTCACTCCATTGGGGAAAGAAAAAAAGTTAGTG

890 900 910 920 930 940 950 960

ACCTCATCCCCTTCAGGTGGAATCAGAGAGAGAGAGGAGCGGAACAATTGGAAGATCGCATCGGGACACTTTAGCGGGAACAGATCCT

c9c11(1>1000) ACCTCATCCCCTTCAGGTGGAATCAGAGAGAGAGAGGAGCGGAACAATTGGAAGATCGCATCGGGACACTTTAGCGGGAACAGATCCT

970 980 990 1000

CTATATTAGCTCTAGGGGACTAGGGCTCCCTT

c9c11(1>1000) CTATATTAGCTCTAGGGGACTAGGGCTCCCTT

10 20 30 40 50 60 70 80

TTCTTTCCAAATTTGGATGGGTGGGATGAGGCCGGCATCAAAGACTTTTCGAAAATCAATGTATAGACCAGTAATTGCGTAGTATAGT

c11c3(1>1000) TTCTTTCCAAATTTGGATGGGTGGGATGAGGCCGGCATCAAAGACTTTTCGAAAATCAATGTATAGACCAGTAATTGCGTAGTATAGT
c11c3.ab1(22>597)

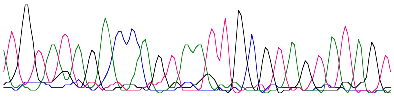

TGTATAGACCAGTAATTGCGTAGTATAGT

90 100 110 120 130 140 150 160 170

AAGTATAGATCAGAGCAGGCACAACATCACTGTCAAGGGCATTCTTATGAGGCAAAAAGCAAAGAGTTGGAGAGGTAAGTAGAAACCC

c11c3(1>1000) AAGTATAGATCAGAGCAGGCACAACATCACTGTCAAGGGCATTCTTATGAGGCAAAAAGCAAAGAGTTGGAGAGGTAAGTAGAAACCC
c11c3.ab1(22>597)

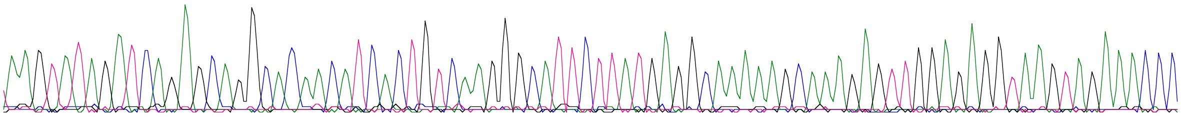

AAGTATAGATCAGAGCAGGCACAACATCACTGTCAAGGGCATTCTTATGAGGCAAAAAGCAAAGAGTTGGAGAGGTAAGTAGAAACCC

180 190 200 210 220 230 240 250 260

GATAGTCACCCCTACATAGTAGGAGGATAGTATGGAAAGCAGGAAGCCCTACTGGCACATCAAATCAAACTTGCTTGGCGCGGGTGCA

c11c3(1>1000) GATAGTCACCCCTACATAGTAGGAGGATAGTATGGAAAGCAGGAAGCCCTACTGGCACATCAAATCAAACTTGCTTGGCGCGGGTGCA
c11c3.ab1(22>597)

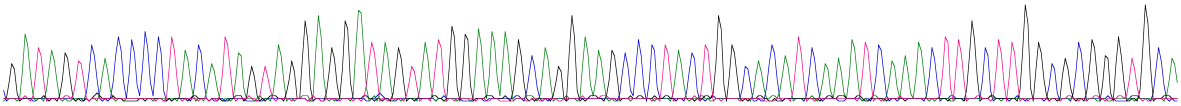

GATAGTCACCCCTACATAGTAGGAGGATAGTATGGAAAGCAGGAAGCCCTACTGGCACATCAAATCAAACTTGCTTGGCGCGGGTGCA

270 280 290 300 310 320 330 340 350

CTAGCTTCTGGCATGGCTGGCGTACTACTTGCTGCCTTGAGGAAGAGGCCCGAGGGACTATCGCAAGAGAGCTGGCAATACTGGATAG

c11c3(1>1000) CTAGCTTCTGGCATGGCTGGCGTACTACTTGCTGCCTTGAGGAAGAGGCCCGAGGGACTATCGCAAGAGAGCTGGCAATACTGGATAG
c11c3.ab1(22>597)

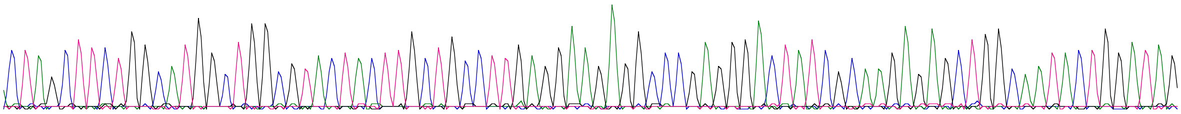

CTAGCTTCTGGCATGGCTGGCGTACTACTTGCTGCCTTGAGGAAGAGGCCCGAGGGACTATCGCAAGAGAGCTGGCAATACTGGATAG

360 370 380 390 400 410 420 430 440

CAAGAACGTTTCAATATTCATCCAACCCTTCCTTTCGTAAATTCCCGTCTTGCTTTTACTTTTAAAGAGTGCAGCCACTTCCGGATCC

c11c3(1>1000) CAAGAACGTTTCAATATTCATCCAACCCTTCCTTTCGTAAATTCCCGTCTTGCTTTTACTTTTAAAGAGTGCAGCCACTTCCGGATCC
c11c3.ab1(22>597)

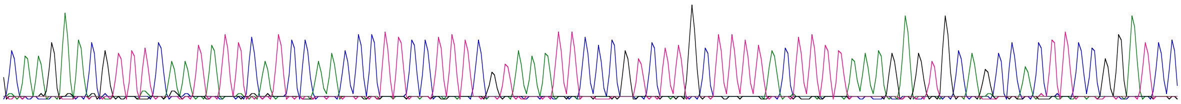

CAAGAACGTTTCAATATTCATCCAACCCTTCCTTTCGTAAATTCCCGTCTTGCTTTTACTTTTAAAGAGTGCAGCCACTTCCGGATCC

450 460 470 480 490 500 510 520

GGATTCAATGATTGTGGAGTGAACGAAGCCAAGTCCAGTATTGTTTTATGAAAAAAAAGGAGATGGGATGGGAACTTATTAGAGTGTG

c11c3(1>1000) GGATTCAATGATTGTGGAGTGAACGAAGCCAAGTCCAGTATTGTTTTATGAAAAAAAAGGAGATGGGATGGGAACTTATTAGAGTGTG
c11c3.ab1(22>597)

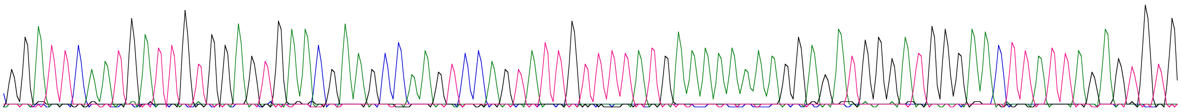

GGATTCAATGATTGTGGAGTGAACGAAGCCAAGTCCAGTATTGTTTTATGAAAAAAAAGGAGATGGGATGGGAACTTATTAGAGTGTG

530 540 550 560 570 580 590 600 610

GCCAAGCCAAGACTCGAGTAGCCAAGTGGATGCAAAGACTAGAAAGCCAGTGAGGGAGGTGCCATCGCTTTAACAGTCTGGAAGCGAC

c11c3(1>1000) → GCCAAGCCAAGACTCGAGTAGCCAAGTGGATGCAAAGACTAGAAAGCCAGTGAGGGAGGTGCCATCGCTTTAACAGTCTGGAAGCGAC

c11c3.ab1(22>597) →

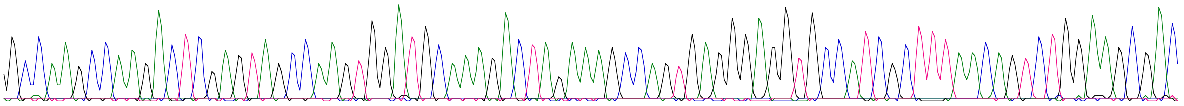

620 630 640 650 660 670 680 690 700

TTCGGCTTGGGTATGCAGAGGCGATAGAAGCAGTGAGGTTACCTGTAAATTGCAAGCGAGTTCTTAGGGTTCCAAACCTTTGAGTTTC

c11c3(1>1000) → TTCGGCTTGGGTATGCAGAGGCGATAGAAGCAGTGAGGTTACCTGTAAATTGCAAGCGAGTTCTTAGGGTTCCAAACCTTTGAGTTTC

c11c3.ab1(22>597) →

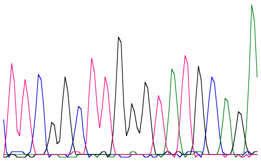

710 720 730 740 750 760 770 780 790

CAGTCTGAGATTGAGATCTTCTTTCACTTTTGGTCTTAGTTAAAAAAGTCTAAAGCGGATAAGGGACTAGCGAGGCGAGTCCAGTTCT

c11c3(1>1000) → CAGTCTGAGATTGAGATCTTCTTTCACTTTTGGTCTTAGTTAAAAAAGTCTAAAGCGGATAAGGGACTAGCGAGGCGAGTCCAGTTCT

800 810 820 830 840 850 860 870 880

CTTCTAGTATGAGGAGGCAAGTTAGTAGTGTCTCTTGCGAGATCTAACCATTCGTTCTAACAAGCGGGTTTCCTTGTTTTTAGGCTTT

c11c3(1>1000) → CTTCTAGTATGAGGAGGCAAGTTAGTAGTGTCTCTTGCGAGATCTAACCATTCGTTCTAACAAGCGGGTTTCCTTGTTTTTAGGCTTT

890 900 910 920 930 940 950 960

TTTTATGTATAGTGCAAACAAGCACGGGAAAAGATACCAATCCAAGAGAGAAAAGAAAGTCCGCGAAATTACATAAAAATAGGATAAA

c11c3(1>1000) → TTTTATGTATAGTGCAAACAAGCACGGGAAAAGATACCAATCCAAGAGAGAAAAGAAAGTCCGCGAAATTACATAAAAATAGGATAAA

970 980 990 1000

GCTTTTTTTATCTAATAAAAGATAGACCAATC

c11c3(1>1000) → GCTTTTTTTATCTAATAAAAGATAGACCAATC

10 20 30 40 50 60 70 80

TTCTTTCCAAATTTGGATGGGTGGGATGAGGCCGGCATCAAAGACTTTTCGAAAATCAATGTATAGACCAGTAATTGCGTAGTATAGT

c11c7(1>1000) → TTCTTTCCAAATTTGGATGGGTGGGATGAGGCCGGCATCAAAGACTTTTCGAAAATCAATGTATAGACCAGTAATTGCGTAGTATAGT

c11c7.ab1(36>578) →

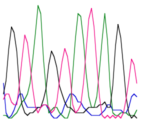

GTAGTATAGT

90 100 110 120 130 140 150 160 170

AAGTATAGATCAGAGCAGGCACAACATCACTGTCAAGGGCATTCTTATGAGGCAAAAAGCAAAGAGTTGGAGAGGTAAGTAGAAACCC

c11c7(1>1000) → AAGTATAGATCAGAGCAGGCACAACATCACTGTCAAGGGCATTCTTATGAGGCAAAAAGCAAAGAGTTGGAGAGGTAAGTAGAAACCC

c11c7.ab1(36>578) →

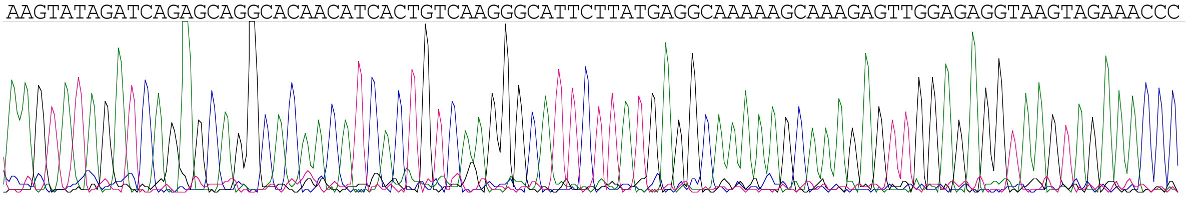

AAGTATAGATCAGAGCAGGCACAACATCACTGTCAAGGGCATTCTTATGAGGCAAAAAGCAAAGAGTTGGAGAGGTAAGTAGAAACCC

180 190 200 210 220 230 240 250 260

GATAGTCACCCCTACATAGTAGGAGGATAGTATGGAAAGCAGGAAGCCCTACTGGCACATCAAATCAAACTTGCTTGGCGCGGGTGCA

c11c7(1>1000) → GATAGTCACCCCTACATAGTAGGAGGATAGTATGGAAAGCAGGAAGCCCTACTGGCACATCAAATCAAACTTGCTTGGCGCGGGTGCA

c11c7.ab1(36>578) →

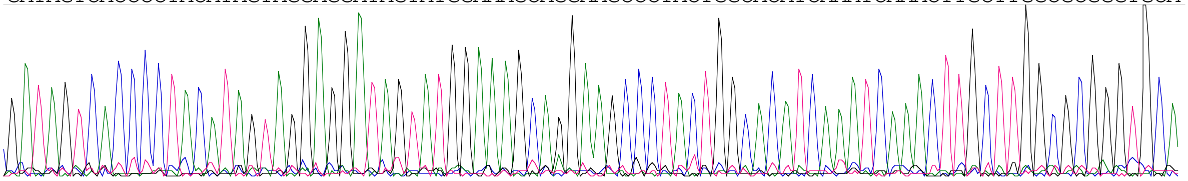

GATAGTCACCCCTACATAGTAGGAGGATAGTATGGAAAGCAGGAAGCCCTACTGGCACATCAAATCAAACTTGCTTGGCGCGGGTGCA

270 280 290 300 310 320 330 340 350

CTAGCTTCTGGCATGGCTGGCGTACTACTTGCTGCCTTGAGGAAGAGGCCCGAGGGACTATCGCAAGAGAGCTGGCAATACTGGATAG

c11c7(1>1000) → CTAGCTTCTGGCATGGCTGGCGTACTACTTGCTGCCTTGAGGAAGAGGCCCGAGGGACTATCGCAAGAGAGCTGGCAATACTGGATAG

c11c7.ab1(36>578) →

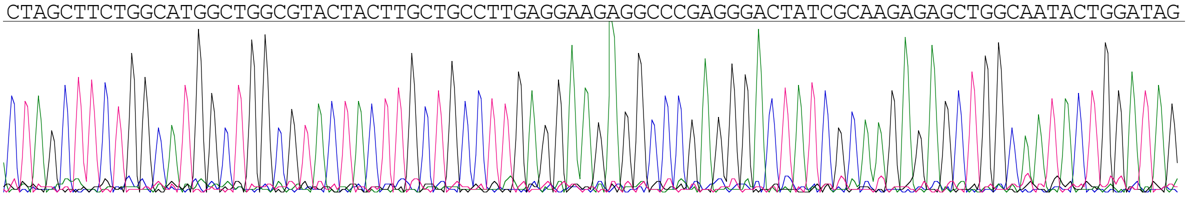

CTAGCTTCTGGCATGGCTGGCGTACTACTTGCTGCCTTGAGGAAGAGGCCCGAGGGACTATCGCAAGAGAGCTGGCAATACTGGATAG

360 370 380 390 400 410 420 430 440

CAAGAACGTTTCAATATTCATCCAACCCTTCCTTTCGTAAATTCCCGTCTTGCTTTTACTTTTAAAGAGTGCAGCCACTTCCGGATCC

c11c7(1>1000) → CAAGAACGTTTCAATATTCATCCAACCCTTCCTTTCGTAAATTCCCGTCTTGCTTTTACTTTTAAAGAGTGCAGCCACTTCCGGATCC

c11c7.ab1(36>578) →

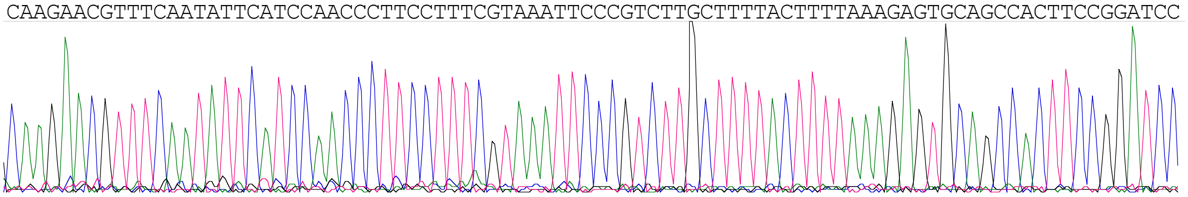

CAAGAACGTTTCAATATTCATCCAACCCTTCCTTTCGTAAATTCCCGTCTTGCTTTTACTTTTAAAGAGTGCAGCCACTTCCGGATCC

450 460 470 480 490 500 510 520

GGATTCAATGATTGTGGAGTGAACGAAGCCAAGTCCAGTATTGTTTTATGAAAAAAAAGGGATCTGGATATCTAATCTAATTTCGTAA

c11c7(1>1000) → GGATTCAATGATTGTGGAGTGAACGAAGCCAAGTCCAGTATTGTTTTATGAAAAAAAAGGGATCTGGATATCTAATCTAATTTCGTAA

c11c7.ab1(36>578) →

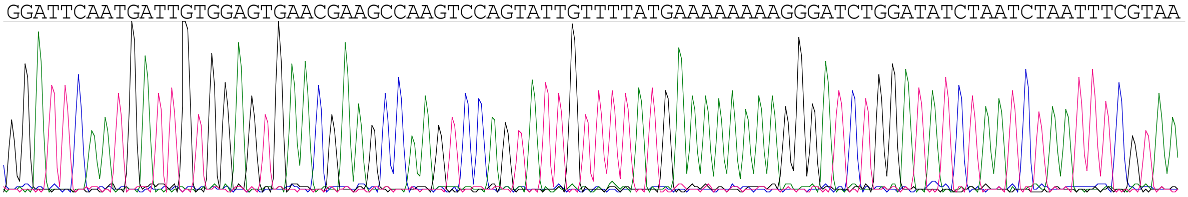

GGATTCAATGATTGTGGAGTGAACGAAGCCAAGTCCAGTATTGTTTTATGAAAAAAAAGGGATCTGGATATCTAATCTAATTTCGTAA

530 540 550 560 570 580 590 600 610

GTAAAAGAAAGTAGCTTTCGCGAGCTATATTTGAGATTCCGTAAGTAACTTAGTGCTTTAAAAACAAGAGTCACGCTCTAGCCCTAAG

c11c7(1>1000) → GTAAAAGAAAGTAGCTTTCGCGAGCTATATTTGAGATTCCGTAAGTAACTTAGTGCTTTAAAAACAAGAGTCACGCTCTAGCCCTAAG

c11c7.ab1(36>578) →

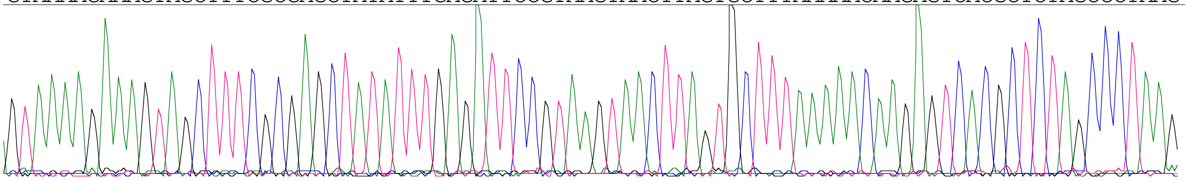

GTAAAAGAAAGTAGCTTTCGCGAGCTATATTTGAGATTCCGTAAGTAACTTAGTGCTTTAAAAACAAGAGTCACGCTCTAGCCCTAAG

620 630 640 650 660 670 680 690 700

AAATAGGCTTCAATCATCGGTACAAATCCAATAAGGGCTTCTTTCTTCCCGGTATACCGCTCTCCAGAGCGAATGAACATAAGTCCAA

c11c7(1>1000) → AAATAGGCTTCAATCATCGGTACAAATCCAATAAGGGCTTCTTTCTTCCCGGTATACCGCTCTCCAGAGCGAATGAACATAAGTCCAA

c11c7.ab1(36>578) →

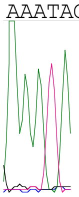

AAATA

710 720 730 740 750 760 770 780 790

CCTAATATCATGAATATCCTTCGCCCGTTATCTCCTCATCTTCCTATTTATAAGCCACAGCTCACTTCGACGTTTCCCATTTTCCGTA

c11c7(1>1000) → CCTAATATCATGAATATCCTTCGCCCGTTATCTCCTCATCTTCCTATTTATAAGCCACAGCTCACTTCGACGTTTCCCATTTTCCGTA

800 810 820 830 840 850 860 870 880

GATCCGTCTTTTCTTTTCTCTTACTTGCGATTGTTTTCATTTTCGTTTATTCGATTATTCTCATGATGGGGCTCGACCCTAACCTATT

c11c7(1>1000) → GATCCGTCTTTTCTTTTCTCTTACTTGCGATTGTTTTCATTTTCGTTTATTCGATTATTCTCATGATGGGGCTCGACCCTAACCTATT

890 900 910 920 930 940 950 960

TACTATTAAAGTAGAATCAATGATCCTAGCGAAGGGACTCTCCTTTTTGTTCCAAAGGATCGGATGTCTTTTTTTGGCAGAAATTATG

c11c7(1>1000) → TACTATTAAAGTAGAATCAATGATCCTAGCGAAGGGACTCTCCTTTTTGTTCCAAAGGATCGGATGTCTTTTTTTGGCAGAAATTATG

970 980 990 1000

ACAGTTGTTTTTCTGGACGAGCTGGCTAACAT

c11c7(1>1000) → ACAGTTGTTTTTCTGGACGAGCTGGCTAACAT

```
                 10        20        30        40        50        60        70        80
                 AAGAACTCAAGGGTTTATTAGAGCAAAATAGCGTTGATTTAGCTCGTTTCTATCGATTAGTAATTTATTAGTTTAGCGGCTTACGAGC
c1-c12c4(1>1000)  →  AAGAACTCAAGGGTTTATTAGAGCAAAATAGCGTTGATTTAGCTCGTTTCTATCGATTAGTAATTTATTAGTTTAGCGGCTTACGAGC

                 90       100       110       120       130       140       150       160       170
                 CAACCAGGATGTATGCTCAAATGCCTTTCACAAGGGCTGAAGTTAGCTTGGGGTAGGGGCTACTAGTGATGGTGCTGCTGCTGGGCAA
c1-c12c4(1>1000)  →  CAACCAGGATGTATGCTCAAATGCCTTTCACAAGGGCTGAAGTTAGCTTGGGGTAGGGGCTACTAGTGATGGTGCTGCTGCTGGGCAA

                 180       190       200       210       220       230       240       250       260
                 GCTGGTTCTTGTAAGGTTGGTTGGGCCGATAGACGGAGATATACGTTGTTGCCGATCCTGCCCATTTCGAGGAAGAAACCTCCGCGCT
c1-c12c4(1>1000)  →  GCTGGTTCTTGTAAGGTTGGTTGGGCCGATAGACGGAGATATACGTTGTTGCCGATCCTGCCCATTTCGAGGAAGAAACCTCCGCGCT
c12c4.ab1(25>567) →
```

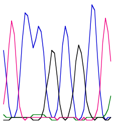

TCCGCGCT

```
                 270       280       290       300       310       320       330       340       350
                 AAGGGATATGGGGAGAATGAACCAGAAGATATAGTTGCATGCCCGGCTAATACGGTTAGATAGACTAGCTGCCCCCGTTCCAACTATG
c1-c12c4(1>1000)  →  AAGGGATATGGGGAGAATGAACCAGAAGATATAGTTGCATGCCCGGCTAATACGGTTAGATAGACTAGCTGCCCCCGTTCCAACTATG
c12c4.ab1(25>567) →
```

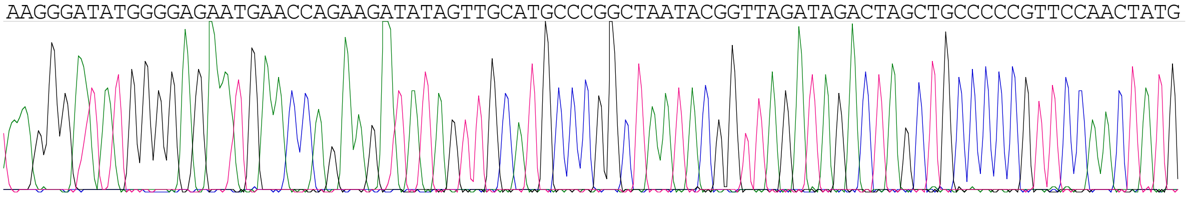

AAGGGATATGGGGAGAATGAACCAGAAGATATAGTTGCATGCCCGGCTAATACGGTTAGATAGACTAGCTGCCCCCGTTCCAACTATG

```
                 360       370       380       390       400       410       420       430       440
                 GGACTGAATCTGGACATTGAGAATGGGTCGACCTATAGAGACTGTCATCCTATCCGGATTGATTGACCGGCCCTCCCACCGGCTATCG
c1-c12c4(1>1000)  →  GGACTGAATCTGGACATTGAGAATGGGTCGACCTATAGAGACTGTCATCCTATCCGGATTGATTGACCGGCCCTCCCACCGGCTATCG
c12c4.ab1(25>567) →
```

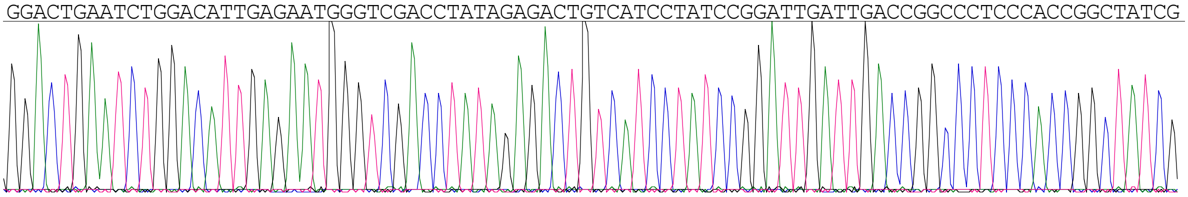

GGACTGAATCTGGACATTGAGAATGGGTCGACCTATAGAGACTGTCATCCTATCCGGATTGATTGACCGGCCCTCCCACCGGCTATCG

```
                 450       460       470       480       490       500       510       520
                 AACTCAACCCGGGCATCCCTCCCTTCCCTTTCTTTAGTACATCTTCCCACCCTTGCTGAAATGGATTGCCTTCAAGCCTCTTTCTTTG
c1-c12c4(1>1000)  →  AACTCAACCCGGGCATCCCTCCCTTCCCTTTCTTTAGTACATCTTCCCACCCTTGCTGAAATGGATTGCCTTCAAGCCTCTTTCTTTG
c12c4.ab1(25>567) →
```

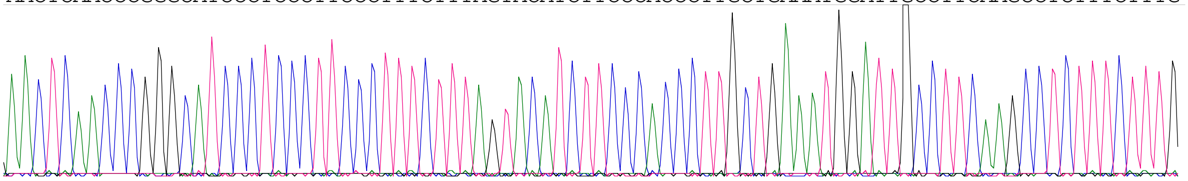

AACTCAACCCGGGCATCCCTCCCTTCCCTTTCTTTAGTACATCTTCCCACCCTTGCTGAAATGGATTGCCTTCAAGCCTCTTTCTTTG

```
                 530       540       550       560       570       580       590       600       610
                 ATTAAGAGGGAGAGTTTGCTAAATCAGATCTGGAAACCTCGACTCTCGTCCTATGTAGGGTATCACATTCAAAGGCTCAACAAGTAAA
c1-c12c4(1>1000)  →  ATTAAGAGGGAGAGTTTGCTAAATCAGATCTGGAAACCTCGACTCTCGTCCTATGTAGGGTATCACATTCAAAGGCTCAACAAGTAAA
c12c4.ab1(25>567) →
```

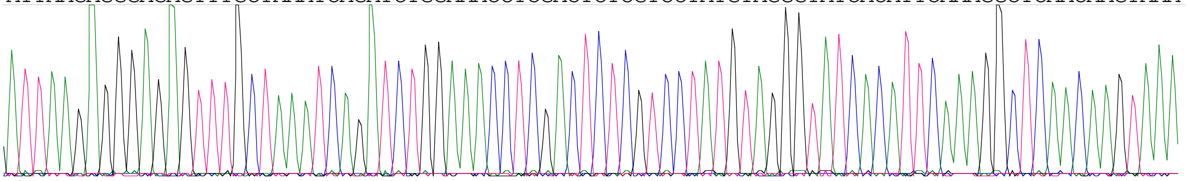

ATTAAGAGGGAGAGTTTGCTAAATCAGATCTGGAAACCTCGACTCTCGTCCTATGTAGGGTATCACATTCAAAGGCTCAACAAGTAAA

620 630 640 650 660 670 680 690 700

ACTGTCATATCAGCAAAGATAGGTCAACCAAGCAAGAGAGCATAACGAGATAAAAGTATGGAGGCTTTCCAACAGTAAAGTAGTAAAG

c1-c12c4(1>1000) → ACTGTCATATCAGCAAAGATAGGTCAACCAAGCAAGAGAGCATAACGAGATAAAAGTATGGAGGCTTTCCAACAGTAAAGTAGTAAAG

c12c4.ab1(25>567) →

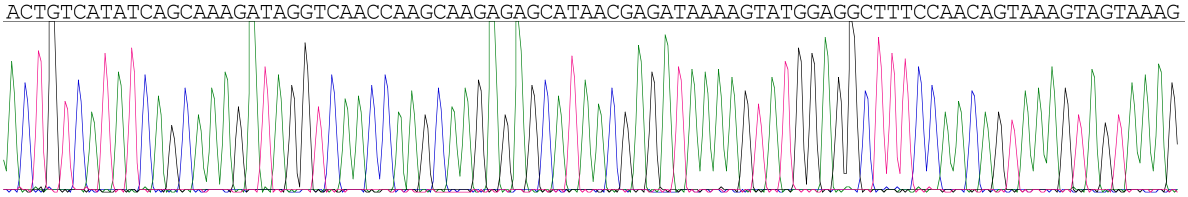

ACTGTCATATCAGCAAAGATAGGTCAACCAAGCAAGAGAGCATAACGAGATAAAAGTATGGAGGCTTTCCAACAGTAAAGTAGTAAAG

710 720 730 740 750 760 770 780 790

TAAGATAATGGGCTTGTTCCCAGCTATTCAATCTAAGCAGACAAAGAGCTGACGAGTCAACGGCGGGAAAGCCCTTTCTTTTCTGGTA

c1-c12c4(1>1000) → TAAGATAATGGGCTTGTTCCCAGCTATTCAATCTAAGCAGACAAAGAGCTGACGAGTCAACGGCGGGAAAGCCCTTTCTTTTCTGGTA

c12c4.ab1(25>567) →

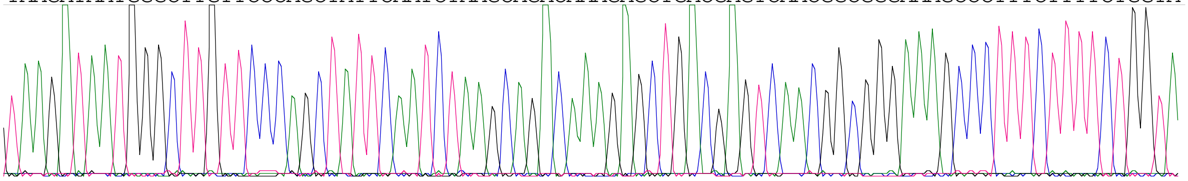

TAAGATAATGGGCTTGTTCCCAGCTATTCAATCTAAGCAGACAAAGAGCTGACGAGTCAACGGCGGGAAAGCCCTTTCTTTTCTGGTA

800 810 820 830 840 850 860 870 880

GACCTTTCATACACGCCTACTTTAGAAGTAACTTCCGGTGCCAATACCGTGTTTTTGGCTTCAAATCGATCTTTTAGCAATCCACTCC

c1-c12c4(1>1000) → GACCTTTCATACACGCCTACTTTAGAAGTAACTTCCGGTGCCAATACCGTGTTTTTGGCTTCAAATCGATCTTTTAGCAATCCACTCC

c12c4.ab1(25>567) →

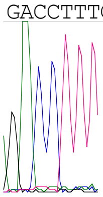

GACCTTT

890 900 910 920 930 940 950 960

ATCTTTGGCAGAGGTAACCAGGTCAACCAGCTGGAGTTTCTGTCCATTCAAAGTACAATCACCACCTCTTCCTGTAACGAAGGTGCCT

c1-c12c4(1>1000) → ATCTTTGGCAGAGGTAACCAGGTCAACCAGCTGGAGTTTCTGTCCATTCAAAGTACAATCACCACCTCTTCCTGTAACGAAGGTGCCT

970 980 990 1000

TCATTTTCTATTCCAGTAGGGCTGCTTGAAGT

c1-c12c4(1>1000) → TCATTTTCTATTCCAGTAGGGCTGCTTGAAGT

10 20 30 40 50 60 70 80

AAGAACTCAAGGGTTTATTAGAGCAAAATAGCGTTGATTTAGCTCGTTTCTATCGATTAGTAATTTATTAGTTTAGCGGCTTACGAG

c12c10(1>1000) → AAGAACTCAAGGGTTTATTAGAGCAAAATAGCGTTGATTTAGCTCGTTTCTATCGATTAGTAATTTATTAGTTTAGCGGCTTACGAG

90 100 110 120 130 140 150 160 170

CCAACCAGGATGTATGCTCAAATGCCTTTCACAAGGGCTGAAGTTAGCTTGGGGTAGGGGCTACTAGTGATGGTGCTGCTGCTGGGC

c12c10(1>1000) → CCAACCAGGATGTATGCTCAAATGCCTTTCACAAGGGCTGAAGTTAGCTTGGGGTAGGGGCTACTAGTGATGGTGCTGCTGCTGGGC

180 190 200 210 220 230 240 250 260

AAGCTGGTTCTTGTAAGGTTGGTTGGGCCGATAGACGGAGATATACGTTGTTGCCGATCCTGCCCATTTCGAGGAAGAAACCTCCGC

c12c10(1>1000) → AAGCTGGTTCTTGTAAGGTTGGTTGGGCCGATAGACGGAGATATACGTTGTTGCCGATCCTGCCCATTTCGAGGAAGAAACCTCCGC

c12c10.ab1(21>631) →

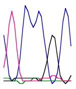

270 280 290 300 310 320 330 340

GCTAAGGGATATGGGGAGAATGAACCAGAAGATATAGTTGCATGCCCGGCTAATACGGTTAGATAGACTAGCTGCCCCCGTTCCAAC

c12c10(1>1000) → GCTAAGGGATATGGGGAGAATGAACCAGAAGATATAGTTGCATGCCCGGCTAATACGGTTAGATAGACTAGCTGCCCCCGTTCCAAC

c12c10.ab1(21>631) →

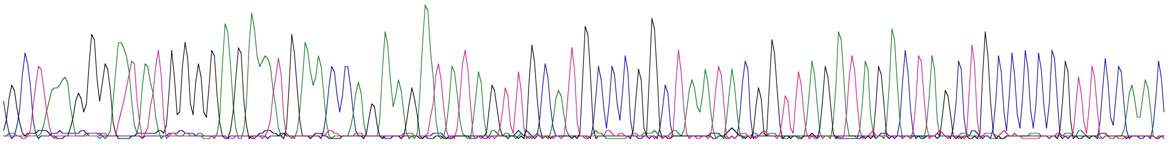

350 360 370 380 390 400 410 420 430

TATGGGACTGAATCTGGACATTGAGAATGGGTCGACCTATAGAGACTGTCATCCTATCCGGATTGATTGACCGGCCCTCCCACCGGC

c12c10(1>1000) → TATGGGACTGAATCTGGACATTGAGAATGGGTCGACCTATAGAGACTGTCATCCTATCCGGATTGATTGACCGGCCCTCCCACCGGC

c12c10.ab1(21>631) →

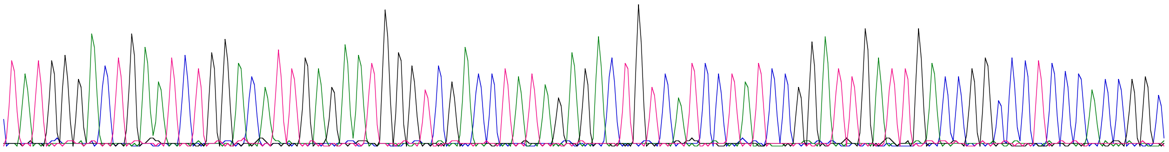

440 450 460 470 480 490 500 510 520

TATCGAACTCAACCCGGGCATCCCTCCCTTCCCTTTCTTTAGTACATCTTCCCACCCTTGCTGAAGCGGCTTGTTGGGCATTTTTCG

c12c10(1>1000) → TATCGAACTCAACCCGGGCATCCCTCCCTTCCCTTTCTTTAGTACATCTTCCCACCCTTGCTGAAGCGGCTTGTTGGGCATTTTTCG

c12c10.ab1(21>631) →

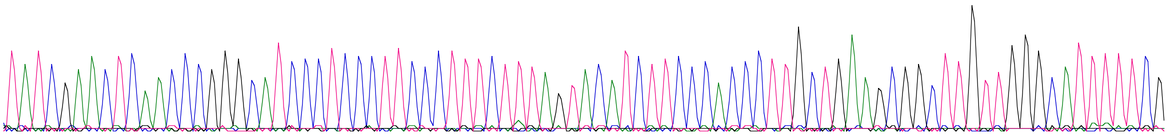

530 540 550 560 570 580 590 600

GATAAGAGGCTATGTCAAAGGCACAAATTACCTACCTATACCTTGGAAGGTGTCAAGATACGTGAACAGCTCAGTTTCAACTATATA

c12c10(1>1000) → GATAAGAGGCTATGTCAAAGGCACAAATTACCTACCTATACCTTGGAAGGTGTCAAGATACGTGAACAGCTCAGTTTCAACTATATA

c12c10.ab1(21>631) →

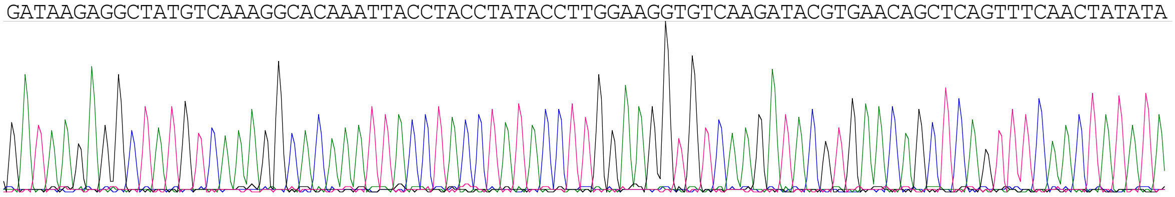

610 620 630 640 650 660 670 680 690

ACGAATACAAAAAGGGTGTAGTTTAGGAGTCAAGAGTACTTTGGACTCGTCACATGACGATGATTCTGCTTACGAATACGAGTATAC

c12c10(1>1000) → ACGAATACAAAAAGGGTGTAGTTTAGGAGTCAAGAGTACTTTGGACTCGTCACATGACGATGATTCTGCTTACGAATACGAGTATAC
c12c10.ab1(21>631) →

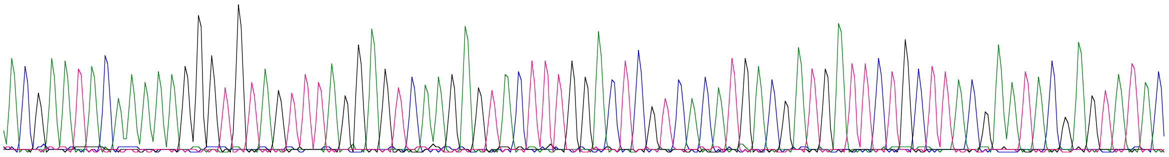

ACGAATACAAAAAGGGTGTAGTTTAGGAGTCAAGAGTACTTTGGACTCGTCACATGACGATGATTCTGCTTACGAATACGAGTATAC

700 710 720 730 740 750 760 770 780

TATCTGCTTACCTTTAAAATACGAAGAATCCTCTAGCTTCCTCCCCTCCGTCAAGGCTATGGGCCGGGAAAACGTGGGTAGGCTGCC

c12c10(1>1000) → TATCTGCTTACCTTTAAAATACGAAGAATCCTCTAGCTTCCTCCCCTCCGTCAAGGCTATGGGCCGGGAAAACGTGGGTAGGCTGCC
c12c10.ab1(21>631) →

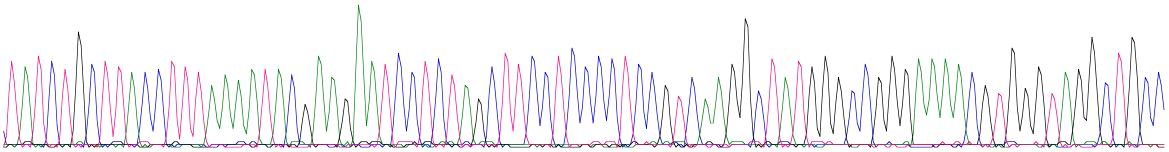

TATCTGCTTACCTTTAAAATACGAAGAATCCTCTAGCTTCCTCCCCTCCGTCAAGGCTATGGGCCGGGAAAACGTGGGTAGGCTGCC

790 800 810 820 830 840 850 860 870

CAACGACCACGGAAGCAAGATAATATGTCCGTCCTGGTTCTCCGCTGATGAGAAATGCAAATCTTTATCTGCACGAGCGGCAAAGCA

c12c10(1>1000) → CAACGACCACGGAAGCAAGATAATATGTCCGTCCTGGTTCTCCGCTGATGAGAAATGCAAATCTTTATCTGCACGAGCGGCAAAGCA
c12c10.ab1(21>631) →

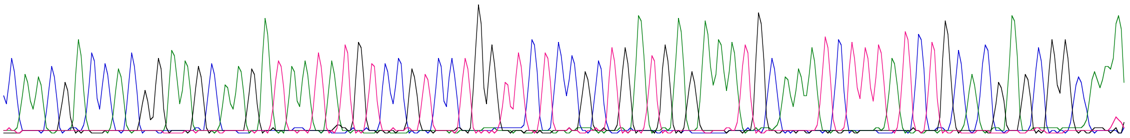

CAACGACCACGGAAGCAAGATAATATGTCCGTCCTGGTTCTCCGCTGATGAGAAATGCAAATCTTTATCTGCACGAGCGGCAAA

880 890 900 910 920 930 940 950

GCAAACGCCAGAGATAGCCCCGCCCGAAACGCACTCACTTCCTCCAAGGAAATTATTAGAATATATTTATTCTATTTATGAAGGGTT

c12c10(1>1000) → GCAAACGCCAGAGATAGCCCCGCCCGAAACGCACTCACTTCCTCCAAGGAAATTATTAGAATATATTTATTCTATTTATGAAGGGTT

960 970 980 990 1000

AATAATATCTTTTTTAAGGGAAGAGTTTCGTCCCCTGCTAGTA

c12c10(1>1000) → AATAATATCTTTTTTAAGGGAAGAGTTTCGTCCCCTGCTAGTA

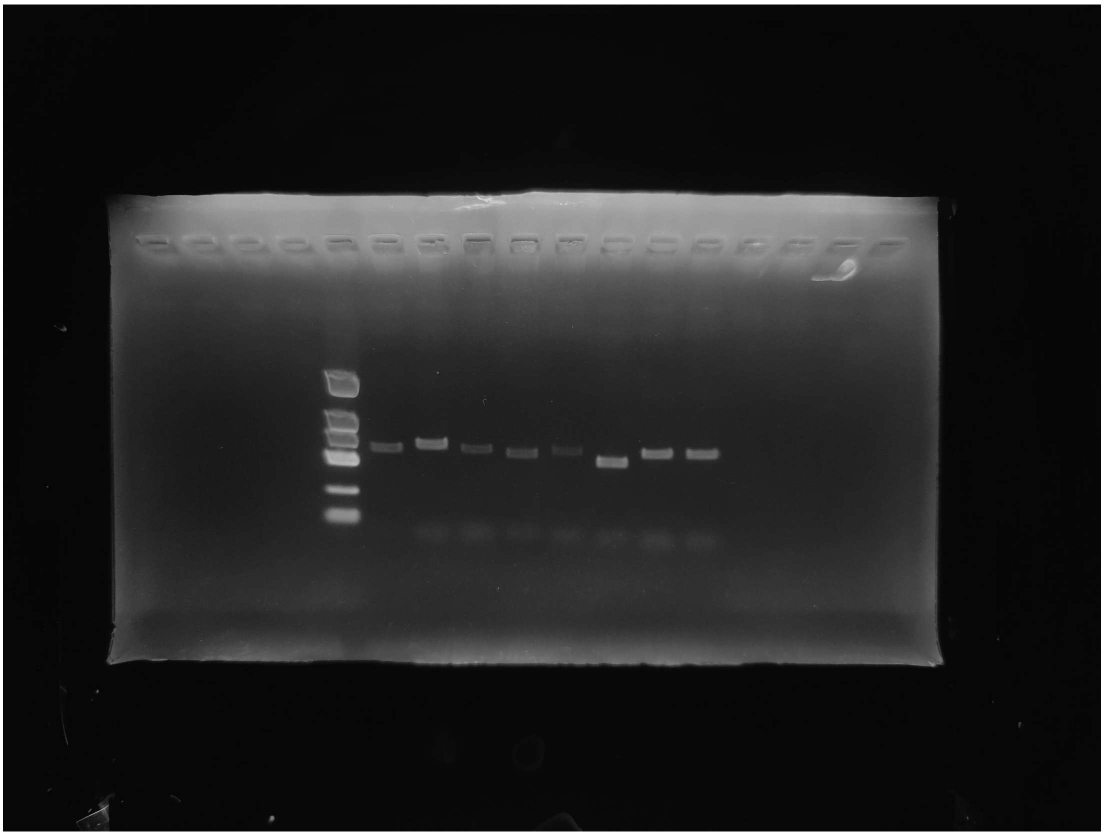

Figure S5. The raw Gel diagram of agarose gel electrophoresis for *Camellia lanceoleosa.*

Figure S6 Graphical results of key linkage positions in the *Camellia lanceoleosa* mitochondrial genome obtained from Sanger sequencing

10 20 30 40 50 60 70 80

AGAAAGTAGCTCGTGGAGAAGAACTCAAGGGTTTATTAGAGCAAAATAGCGTTGATTTAGCTCGTTTCTATCGATTAGTAATTTATTAG

c2c3(1>1000) AGAAAGTAGCTCGTGGAGAAGAACTCAAGGGTTTATTAGAGCAAAATAGCGTTGATTTAGCTCGTTTCTATCGATTAGTAATTTATTAG

90 100 110 120 130 140 150 160 170

TTTAGCGGCTTACGAGCCAACCAGGATGTATGCTCAAATGCCTTTCACAAGGGCTGAAGTTAGCTTGGGGTAGGGGCTACTAGTGATGG

c2c3(1>1000) TTTAGCGGCTTACGAGCCAACCAGGATGTATGCTCAAATGCCTTTCACAAGGGCTGAAGTTAGCTTGGGGTAGGGGCTACTAGTGATGG
c2c3.ab1(25>593)

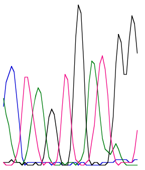

CTAGTGATGG

180 190 200 210 220 230 240 250 260

TGCTGCTGCTGGGCAAGCTGGTTCTTGTAAGGTTGGTTGGGCCGATAGACGGAGATATACGTTGTTGCCGATCCTGCCCATTTCGAGGA

c2c3(1>1000) TGCTGCTGCTGGGCAAGCTGGTTCTTGTAAGGTTGGTTGGGCCGATAGACGGAGATATACGTTGTTGCCGATCCTGCCCATTTCGAGGA
c2c3.ab1(25>593)

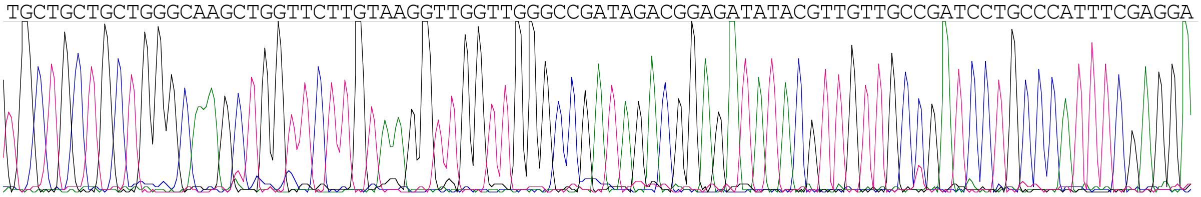

TGCTGCTGCTGGGCAAGCTGGTTCTTGTAAGGTTGGTTGGGCCGATAGACGGAGATATACGTTGTTGCCGATCCTGCCCATTTCGAGGA

270 280 290 300 310 320 330 340 350

AGAAACCTCCGCGCTAAGGGATATGGGGAGAATGAACCAGAAGATATAGTTGCATGCCCGGCTAATACGGTTAGATAGACTAGCTGCCC

c2c3(1>1000) AGAAACCTCCGCGCTAAGGGATATGGGGAGAATGAACCAGAAGATATAGTTGCATGCCCGGCTAATACGGTTAGATAGACTAGCTGCCC
c2c3.ab1(25>593)

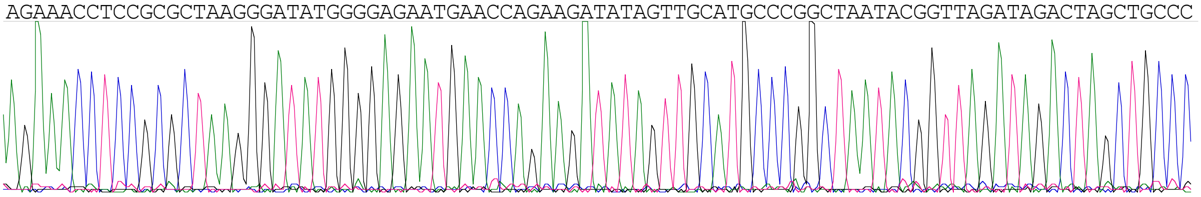

AGAAACCTCCGCGCTAAGGGATATGGGGAGAATGAACCAGAAGATATAGTTGCATGCCCGGCTAATACGGTTAGATAGACTAGCTGCCC

360 370 380 390 400 410 420 430 440

CCGTTCCAACTATGGGACTGAATCTGGACATTGAGAATGGGTCGACCTATAGAGACTGTCATCCTATCCGGATTGATTGACCGGCCCTC

c2c3(1>1000) CCGTTCCAACTATGGGACTGAATCTGGACATTGAGAATGGGTCGACCTATAGAGACTGTCATCCTATCCGGATTGATTGACCGGCCCTC
c2c3.ab1(25>593)

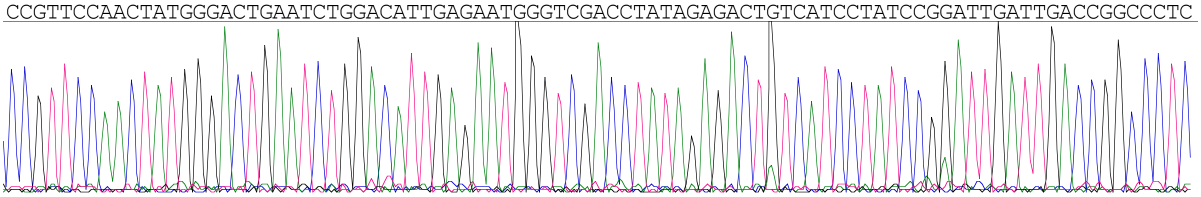

CCGTTCCAACTATGGGACTGAATCTGGACATTGAGAATGGGTCGACCTATAGAGACTGTCATCCTATCCGGATTGATTGACCGGCCCTC

450 460 470 480 490 500 510 520 530

CCACCGGCTATCGAACTCAACCCGGGCATCCCTCCCTTCCCTTTCTTTAGTACATCTTCCCACCCTTGCTGAAATGGATTGCCTTCAAG

c2c3(1>1000) CCACCGGCTATCGAACTCAACCCGGGCATCCCTCCCTTCCCTTTCTTTAGTACATCTTCCCACCCTTGCTGAAATGGATTGCCTTCAAG
c2c3.ab1(25>593)

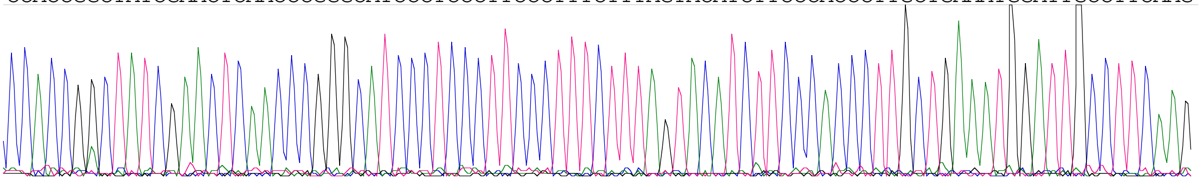

CCACCGGCTATCGAACTCAACCCGGGCATCCCTCCCTTCCCTTTCTTTAGTACATCTTCCCACCCTTGCTGAAATGGATTGCCTTCAAG

540 550 560 570 580 590 600 610 620

CCTCTTTCTTTGATTAAGAGGGAGAGTTTGCTAAATCAGATCTGGAAACCTCGACTCTCGTCCTATGTAGGGTATCACATTCAAAGGCT

c2c3(1>1000) → CCTCTTTCTTTGATTAAGAGGGAGAGTTTGCTAAATCAGATCTGGAAACCTCGACTCTCGTCCTATGTAGGGTATCACATTCAAAGGCT

c2c3.ab1(25>593) →

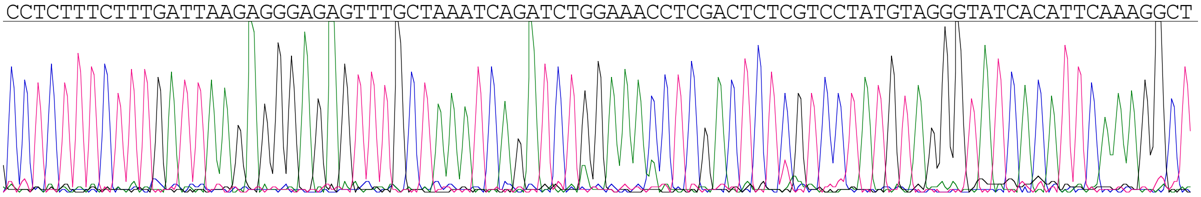

CCTCTTTCTTTGATTAAGAGGGAGAGTTTGCTAAATCAGATCTGGAAACCTCGACTCTCGTCCTATGTAGGGTATCACATTCAAAGGCT

630 640 650 660 670 680 690 700 710

CAACAAGTAAAACTGTCATATCAGCAAAGATAGGTCAACCAAGCAAGAGAGCATAACGAGATAAAAGTATGGAGGCTTTCCAACAGTAA

c2c3(1>1000) → CAACAAGTAAAACTGTCATATCAGCAAAGATAGGTCAACCAAGCAAGAGAGCATAACGAGATAAAAGTATGGAGGCTTTCCAACAGTAA

c2c3.ab1(25>593) →

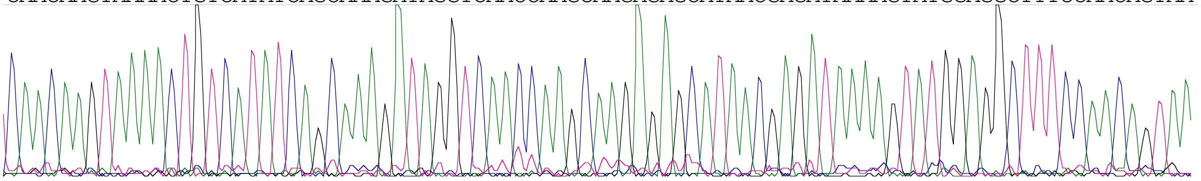

CAACAAGTAAAACTGTCATATCAGCAAAGATAGGTCAACCAAGCAAGAGAGCATAACGAGATAAAAGTATGGAGGCTTTCCAACAGTAA

720 730 740 750 760 770 780 790 800

AGTAGTAAAGTAAGATAATGGGCTTGTTCCCAGCTATTCAATCTAAGCAGACAAAGAGCTGACGAGTCAACGGCGGGAAAGCCCTTTCT

c2c3(1>1000) → AGTAGTAAAGTAAGATAATGGGCTTGTTCCCAGCTATTCAATCTAAGCAGACAAAGAGCTGACGAGTCAACGGCGGGAAAGCCCTTTCT

c2c3.ab1(25>593) →

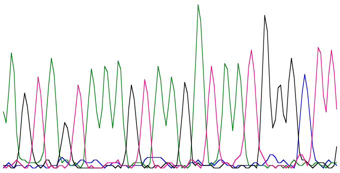

AGTAGTAAAGTAAGATAATGGGCTT

810 820 830 840 850 860 870 880 890

TTTCTGGTAGACCTTTCATACACGCCTACTTTAGAAGTAACTTCCGGTGCCAATACCGTGTTTTTGGCTTCAAATCGATCTTTTAGCAA

c2c3(1>1000) → TTTCTGGTAGACCTTTCATACACGCCTACTTTAGAAGTAACTTCCGGTGCCAATACCGTGTTTTTGGCTTCAAATCGATCTTTTAGCAA

900 910 920 930 940 950 960 970

TCCACTCCATCTTTGGCAGAGGTAACCAGGTCAACCAGCTGGAGTTTCTGTCCATTCAAAGTACAATCACCACCTCTTCCTGTAACGAA

c2c3(1>1000) → TCCACTCCATCTTTGGCAGAGGTAACCAGGTCAACCAGCTGGAGTTTCTGTCCATTCAAAGTACAATCACCACCTCTTCCTGTAACGAA

980 990 1000

GGTGCCTTCATTTTCTATTCC

c2c3(1>1000) → GGTGCCTTCATTTTCTATTCC

10 20 30 40 50 60 70 80

GTACTAGCAGGGGACGAAACTCTTCCCTTAAAAAAGATATTATTAACCCTTCATAAATAGAATAAATATATTCTAATAATTTCCTTGGA

c2c6(1>1000) → GTACTAGCAGGGGACGAAACTCTTCCCTTAAAAAAGATATTATTAACCCTTCATAAATAGAATAAATATATTCTAATAATTTCCTTGGA

90 100 110 120 130 140 150 160 170

GGAAGTGAGTGCGTTTCGGGCGGGGCTATCTCTGGCGTTTGCTGCTTTGCCGCTCGTGCAGATAAAGATTTGCATTTCTCATCAGCGGA

c2c6(1>1000) → GGAAGTGAGTGCGTTTCGGGCGGGGCTATCTCTGGCGTTTGCTGCTTTGCCGCTCGTGCAGATAAAGATTTGCATTTCTCATCAGCGGA

180 190 200 210 220 230 240 250 260

GAACCAGGACGGACATATTATCTTGCTTCCGTGGTCGTTGGGCAGCCTACCCACGTTTTCCCGGCCCATAGCCTTGACGGAGGGGAGGA

c2c6(1>1000) → GAACCAGGACGGACATATTATCTTGCTTCCGTGGTCGTTGGGCAGCCTACCCACGTTTTCCCGGCCCATAGCCTTGACGGAGGGGAGGA
c2c6.ab1(23>636) →

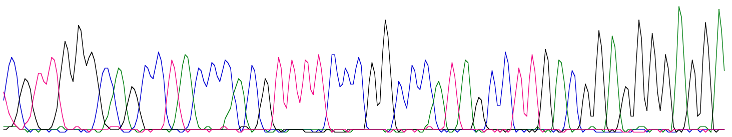

CGTTGGGCAGCCTACCCACGTTTTCCCGGCCCATAGCCTTGACGGAGGGGAGGA

270 280 290 300 310 320 330 340 350

AGCTAGAGGATTCTTCGTATTTTAAAGGTAAGCAGATAGTATACTCGTATTCGTAAGCAGAATCATCGTCATGTGACGAGTCCAAAGTA

c2c6(1>1000) → AGCTAGAGGATTCTTCGTATTTTAAAGGTAAGCAGATAGTATACTCGTATTCGTAAGCAGAATCATCGTCATGTGACGAGTCCAAAGTA
c2c6.ab1(23>636) →

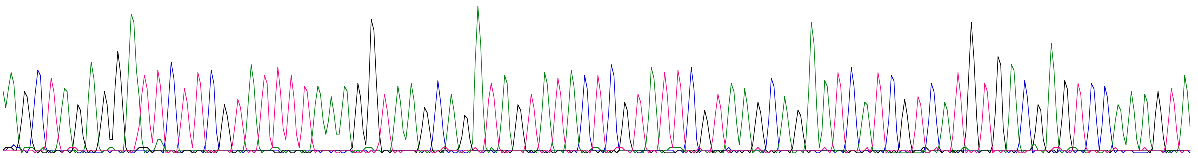

AGCTAGAGGATTCTTCGTATTTTAAAGGTAAGCAGATAGTATACTCGTATTCGTAAGCAGAATCATCGTCATGTGACGAGTCCAAAGTA

360 370 380 390 400 410 420 430 440

CTCTTGACTCCTAAACTACACCCTTTTTGTATTCGTTATATAGTTGAAACTGAGCTGTTCACGTATCTTGACACCTTCCAAGGTATAGG

c2c6(1>1000) → CTCTTGACTCCTAAACTACACCCTTTTTGTATTCGTTATATAGTTGAAACTGAGCTGTTCACGTATCTTGACACCTTCCAAGGTATAGG
c2c6.ab1(23>636) →

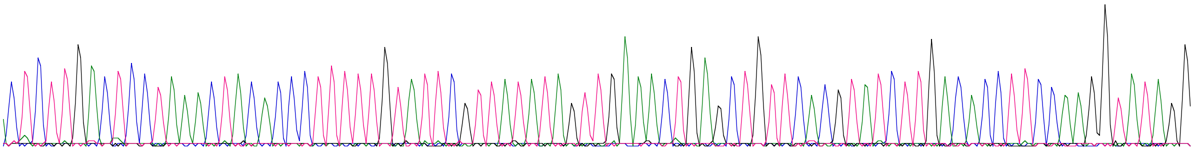

CTCTTGACTCCTAAACTACACCCTTTTTGTATTCGTTATATAGTTGAAACTGAGCTGTTCACGTATCTTGACACCTTCCAAGGTATAGG

450 460 470 480 490 500 510 520 530

TAGGTAATTTGTGCCTTTGACATAGCCTCTTATCCGAAAAATGCCCAACAAGCCGCTTCCCACCCTTGCTGAAATGGATTGCCTTCAAG

c2c6(1>1000) → TAGGTAATTTGTGCCTTTGACATAGCCTCTTATCCGAAAAATGCCCAACAAGCCGCTTCCCACCCTTGCTGAAATGGATTGCCTTCAAG
c2c6.ab1(23>636) →

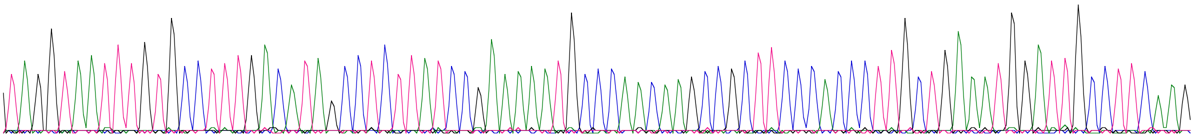

TAGGTAATTTGTGCCTTTGACATAGCCTCTTATCCGAAAAATGCCCAACAAGCCGCTTCCCACCCTTGCTGAAATGGATTGCCTTCAAG

540 550 560 570 580 590 600 610 620

CCTCTTTCTTTGATTAAGAGGGAGAGTTTGCTAAATCAGATCTGGAAACCTCGACTCTCGTCCTATGTAGGGTATCACATTCAAAGGCT

c2c6(1>1000) → CCTCTTTCTTTGATTAAGAGGGAGAGTTTGCTAAATCAGATCTGGAAACCTCGACTCTCGTCCTATGTAGGGTATCACATTCAAAGGCT
c2c6.ab1(23>636) →

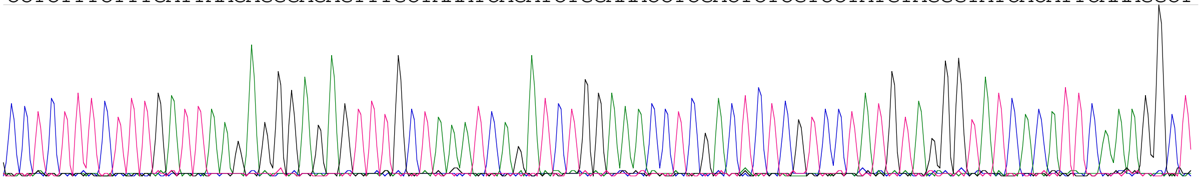

CCTCTTTCTTTGATTAAGAGGGAGAGTTTGCTAAATCAGATCTGGAAACCTCGACTCTCGTCCTATGTAGGGTATCACATTCAAAGGCT

630 640 650 660 670 680 690 700 710

CAACAAGTAAAACTGTCATATCAGCAAAGATAGGTCAACCAAGCAAGAGAGCATAACGAGATAAAAGTATGGAGGCTTTCCAACAGTAA

c2c6(1>1000) → CAACAAGTAAAACTGTCATATCAGCAAAGATAGGTCAACCAAGCAAGAGAGCATAACGAGATAAAAGTATGGAGGCTTTCCAACAGTAA

c2c6.ab1(23>636) →

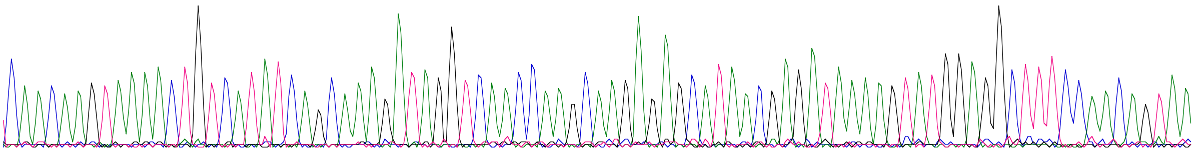

CAACAAGTAAAACTGTCATATCAGCAAAGATAGGTCAACCAAGCAAGAGAGCATAACGAGATAAAAGTATGGAGGCTTTCCAACAGTAA

720 730 740 750 760 770 780 790 800

AGTAGTAAAGTAAGATAATGGGCTTGTTCCCAGCTATTCAATCTAAGCAGACAAAGAGCTGACGAGTCAACGGCGGGAAAGCCCTTTCT

c2c6(1>1000) → AGTAGTAAAGTAAGATAATGGGCTTGTTCCCAGCTATTCAATCTAAGCAGACAAAGAGCTGACGAGTCAACGGCGGGAAAGCCCTTTCT

c2c6.ab1(23>636) →

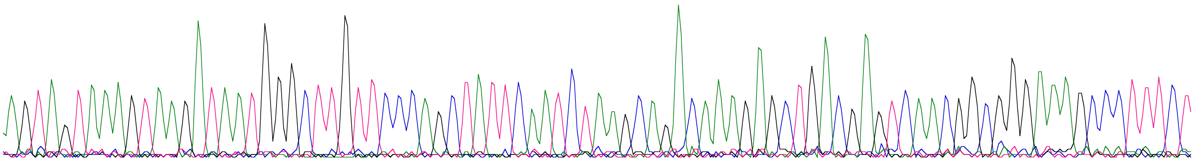

AGTAGTAAAGTAAGATAATGGGCTTGTTCCCAGCTATTCAATCTAAGCAGACAAAGAGCTGACGAGTCAACGGCGGGAAAGCCCTTTCT

810 820 830 840 850 860 870 880 890

TTTCTGGTAGACCTTTCATACACGCCTACTTTAGAAGTAACTTCCGGTGCCAATACCGTGTTTTTGGCTTCAAATCGATCTTTTAGCAA

c2c6(1>1000) → TTTCTGGTAGACCTTTCATACACGCCTACTTTAGAAGTAACTTCCGGTGCCAATACCGTGTTTTTGGCTTCAAATCGATCTTTTAGCAA

c2c6.ab1(23>636) →

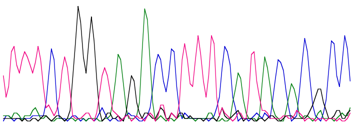

TTTCTGGTAGACCTTTCATACACGCC

900 910 920 930 940 950 960 970

TCCACTCCATCTTTGGCAGAGGTAACCAGGTCAACCAGCTGGAGTTTCTGTCCATTCAAAGTACAATCACCACCTCTTCCTGTAACGAA

c2c6(1>1000) → TCCACTCCATCTTTGGCAGAGGTAACCAGGTCAACCAGCTGGAGTTTCTGTCCATTCAAAGTACAATCACCACCTCTTCCTGTAACGAA

980 990 1000

GGTGCCTTCATTTTCTATTCC

c2c6(1>1000) → GGTGCCTTCATTTTCTATTCC

10 20 30 40 50 60 70 80

AGTAGCTGGTTATAGAGCTTCCGCTGGGTGACGGAGTGCGGGCATCGATCGAAAGAATAAGGGGGATCTAAAGTCTGGAAGGGCCTTCT

c2c7(1>1000) AGTAGCTGGTTATAGAGCTTCCGCTGGGTGACGGAGTGCGGGCATCGATCGAAAGAATAAGGGGGATCTAAAGTCTGGAAGGGCCTTCT

90 100 110 120 130 140 150 160 170

CCTGAGTCGGCCTTTGTTTTTGTTTAAAAGCGTGCTTCCGGTCTTGCATCTCAATACGGTATGGTGCTTTGGCGTCCTGCCTTCCCGAT

c2c7(1>1000) CCTGAGTCGGCCTTTGTTTTTGTTTAAAAGCGTGCTTCCGGTCTTGCATCTCAATACGGTATGGTGCTTTGGCGTCCTGCCTTCCCGAT

180 190 200 210 220 230 240 250 260

TTCTTCTCTGGAGAGATATCCATAGGCTTTCTTTTCTTCTGATTTTTCTCGGACTCTCTTAAGCCTTTCAACGAGAGTTTGGATATCCT

c2c7(1>1000) TTCTTCTCTGGAGAGATATCCATAGGCTTTCTTTTCTTCTGATTTTTCTCGGACTCTCTTAAGCCTTTCAACGAGAGTTTGGATATCCT
c2c7.ab1(34>590)

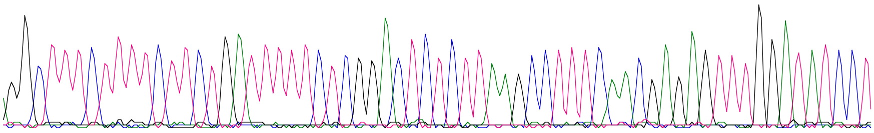

GGCTTTCTTTTCTTCTGATTTTTCTCGGACTCTCTTAAGCCTTTCAACGAGAGTTTGGATATCCT

270 280 290 300 310 320 330 340 350

CATGAGTAAAGGGGTTGTAGTGGACATAACATATAAGTTATTAAAACTGGTGTAGGAACAAAGTACCGATAAAGCAGAATACGCGCCTT

c2c7(1>1000) CATGAGTAAAGGGGTTGTAGTGGACATAACATATAAGTTATTAAAACTGGTGTAGGAACAAAGTACCGATAAAGCAGAATACGCGCCTT
c2c7.ab1(34>590)

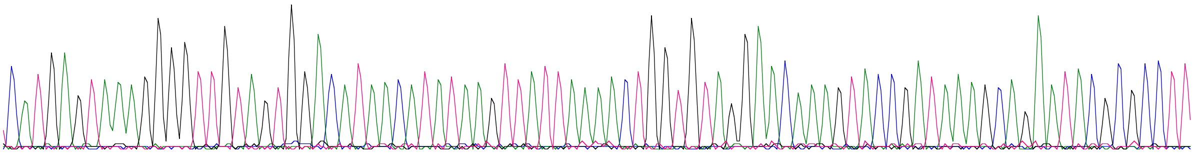

CATGAGTAAAGGGGTTGTAGTGGACATAACATATAAGTTATTAAAACTGGTGTAGGAACAAAGTACCGATAAAGCAGAATACGCGCCTT

360 370 380 390 400 410 420 430 440

CAAGCAACCGGAATAACCAATTTAATATATAGAGAATATCCCCCTACTTCTCTTAGGAGCTGTTAATAAATAAGAAATAGGAGGGGCTA

c2c7(1>1000) CAAGCAACCGGAATAACCAATTTAATATATAGAGAATATCCCCCTACTTCTCTTAGGAGCTGTTAATAAATAAGAAATAGGAGGGGCTA
c2c7.ab1(34>590)

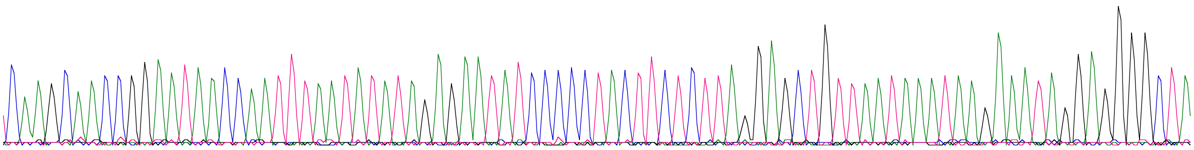

CAAGCAACCGGAATAACCAATTTAATATATAGAGAATATCCCCCTACTTCTCTTAGGAGCTGTTAATAAATAAGAAATAGGAGGGGCTA

450 460 470 480 490 500 510 520 530

GATATGGATCATCCTTCTTTGTTACCCAGGTGGGAACTGGTTTCGTAGAAGAAGTAGTCCGCACCGGTCTTGTGTTACCTCACTAGCTC

c2c7(1>1000) GATATGGATCATCCTTCTTTGTTACCCAGGTGGGAACTGGTTTCGTAGAAGAAGTAGTCCGCACCGGTCTTGTGTTACCTCACTAGCTC
c2c7.ab1(34>590)

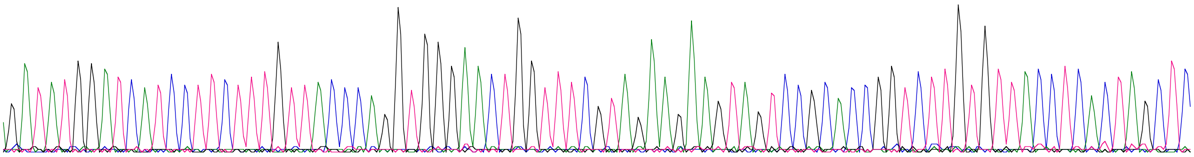

GATATGGATCATCCTTCTTTGTTACCCAGGTGGGAACTGGTTTCGTAGAAGAAGTAGTCCGCACCGGTCTTGTGTTACCTCACTAGCTC

540 550 560 570 580 590 600 610 620

ACCGACCCCGGCATCGCTACGTTCGTTCACTCAAAGAAAACTTCCCCCTTACTGGAGGGGAACTTTCTCATAAGTAATGGGCGCAGTCC

c2c7(1>1000) ACCGACCCCGGCATCGCTACGTTCGTTCACTCAAAGAAAACTTCCCCCTTACTGGAGGGGAACTTTCTCATAAGTAATGGGCGCAGTCC
c2c7.ab1(34>590)

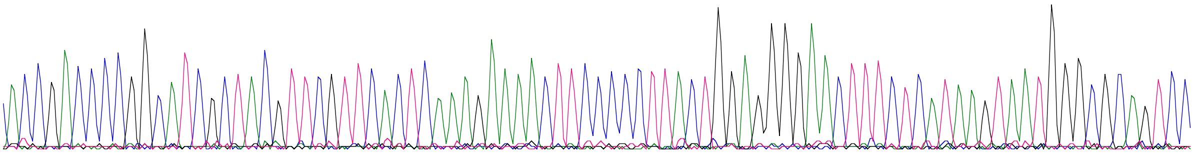

ACCGACCCCGGCATCGCTACGTTCGTTCACTCAAAGAAAACTTCCCCCTTACTGGAGGGGAACTTTCTCATAAGTAATGGGCGCAGTCC

630 640 650 660 670 680 690 700 710

ATATAGGAGACTCGACCAGATCTCTTACCCATTCTCATTTCAAGAGGAAGGCATCCTTGGTTAAGGTATAGACCGAAACCGGTCCAACT

c2c7(1>1000) → ATATAGGAGACTCGACCAGATCTCTTACCCATTCTCATTTCAAGAGGAAGGCATCCTTGGTTAAGGTATAGACCGAAACCGGTCCAACT
c2c7.ab1(34>590) →

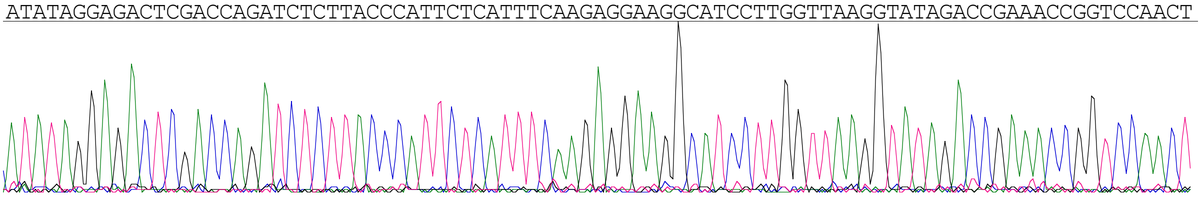

ATATAGGAGACTCGACCAGATCTCTTACCCATTCTCATTTCAAGAGGAAGGCATCCTTGGTTAAGGTATAGACCGAAACCGGTCCAACT

720 730 740 750 760 770 780 790 800

GGTCAAGAAAGCAACCGTCCATTTGCCCTCATTTCACAGGTTGAGCGAAGCTTAGGACAGCATTCAGCTTTCATATGCATTTCTTACAG

c2c7(1>1000) → GGTCAAGAAAGCAACCGTCCATTTGCCCTCATTTCACAGGTTGAGCGAAGCTTAGGACAGCATTCAGCTTTCATATGCATTTCTTACAG
c2c7.ab1(34>590) →

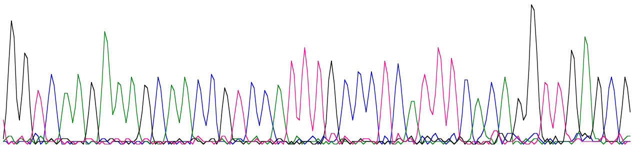

GGTCAAGAAAGCAACCGTCCATTTGCCCTCATTTCACAGGTTGAGCG

810 820 830 840 850 860 870 880 890

ATCGGGATTCTTCGATTACAGCATAGCTATCATCAGAAGCAGTACTTCCGAGTCACTCTATAACTGAGAGCTTGTAGTTGGGGATTTAC

c2c7(1>1000) → ATCGGGATTCTTCGATTACAGCATAGCTATCATCAGAAGCAGTACTTCCGAGTCACTCTATAACTGAGAGCTTGTAGTTGGGGATTTAC

900 910 920 930 940 950 960 970

CAGATCTCTAAGATAGAGTCTATCCGACGTATCACCCGTAATTGGGAGTTCAAACGAGGCCTCAACGTAGATGCTGGATGGGAACTTCT

c2c7(1>1000) → CAGATCTCTAAGATAGAGTCTATCCGACGTATCACCCGTAATTGGGAGTTCAAACGAGGCCTCAACGTAGATGCTGGATGGGAACTTCT

980 990 1000

ATTCTACCTGGCTTATTTATA

c2c7(1>1000) → ATTCTACCTGGCTTATTTATA

10 20 30 40 50 60 70 80

GCAAAGGAGACGTACTATAGGAGATAGAGAACTGCTGCCCGTGGTTTTTATCCCTCGCTCCTGAACTGGCGCACTCCGGCAAGAAGGAA

c3c4(1>1000) → GCAAAGGAGACGTACTATAGGAGATAGAGAACTGCTGCCCGTGGTTTTTATCCCTCGCTCCTGAACTGGCGCACTCCGGCAAGAAGGAA

90 100 110 120 130 140 150 160 170

CTAACGAGAAGTAGGAAAGGTCACACCACTCTTCTGACTAAAGAAAGGGGCTATTTCAGTTCATCTGTTTGCTCGTTGAAAGAGACTGC

c3c4(1>1000) → CTAACGAGAAGTAGGAAAGGTCACACCACTCTTCTGACTAAAGAAAGGGGCTATTTCAGTTCATCTGTTTGCTCGTTGAAAGAGACTGC

180 190 200 210 220 230 240 250 260

TTCAACAAAGCCGTATCTAGCTGGGCCGTAGTTTGCTGATTCGGAGGATAAGTCCTGAGGGGTAGGGACTTTCATCAAGGGTTCTGGGA

c3c4(1>1000) → TTCAACAAAGCCGTATCTAGCTGGGCCGTAGTTTGCTGATTCGGAGGATAAGTCCTGAGGGGTAGGGACTTTCATCAAGGGTTCTGGGA

270 280 290 300 310 320 330 340 350

ACGCCCCTATCTATAGGGATAGCTCCCGACATCAATATCTATGGATTAAAGCAACTCCCTCTCGTAGTTGATCCCTTGTTCACAGCTTT

c3c4(1>1000) → ACGCCCCTATCTATAGGGATAGCTCCCGACATCAATATCTATGGATTAAAGCAACTCCCTCTCGTAGTTGATCCCTTGTTCACAGCTTT

c3c4.ab1(32>550) →

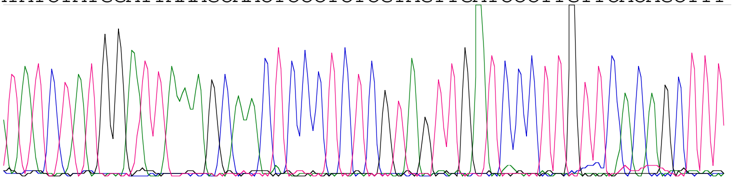

TATCTATGGATTAAAGCAACTCCCTCTCGTAGTTGATCCCTTGTTCACAGCTTT

360 370 380 390 400 410 420 430 440

ACTCGGCTCATGCACTCGGCCCGTATGTAAGAGACGGGGACTCCTACTTCCTTGTTAGCAACTTATCCGCTCGGGTCAAAGCCCTTCTC

c3c4(1>1000) → ACTCGGCTCATGCACTCGGCCCGTATGTAAGAGACGGGGACTCCTACTTCCTTGTTAGCAACTTATCCGCTCGGGTCAAAGCCCTTCTC

c3c4.ab1(32>550) →

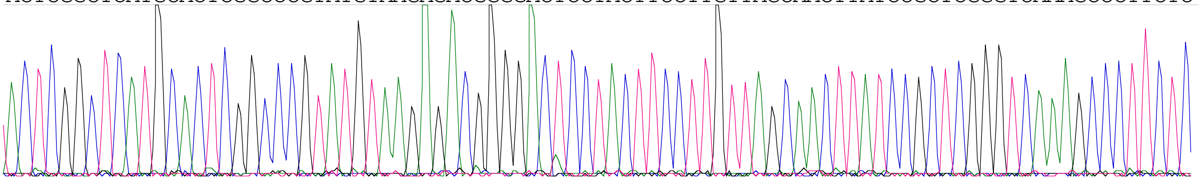

ACTCGGCTCATGCACTCGGCCCGTATGTAAGAGACGGGGACTCCTACTTCCTTGTTAGCAACTTATCCGCTCGGGTCAAAGCCCTTCTC

450 460 470 480 490 500 510 520 530

TTCTTAGTCTGGTGCTTCCTTCATCGAATGAGTATTCATCCGTTGAGCTATCCCTCGAATCAGCAAACAGAATAAAAGCAAGATACGGC

c3c4(1>1000) → TTCTTAGTCTGGTGCTTCCTTCATCGAATGAGTATTCATCCGTTGAGCTATCCCTCGAATCAGCAAACAGAATAAAAGCAAGATACGGC

c3c4.ab1(32>550) →

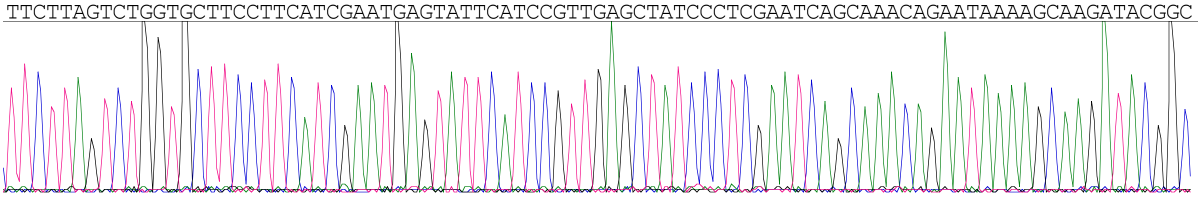

TTCTTAGTCTGGTGCTTCCTTCATCGAATGAGTATTCATCCGTTGAGCTATCCCTCGAATCAGCAAACAGAATAAAAGCAAGATACGGC

540 550 560 570 580 590 600 610 620

TTATCAGCATACTTATTGATCTCCTGAATATTCGGCTAAATCCCCTGGCTATTGAACTGAGCTATCCTCACCTACTGACCGGAAAGCAC

c3c4(1>1000) → TTATCAGCATACTTATTGATCTCCTGAATATTCGGCTAAATCCCCTGGCTATTGAACTGAGCTATCCTCACCTACTGACCGGAAAGCAC

c3c4.ab1(32>550) →

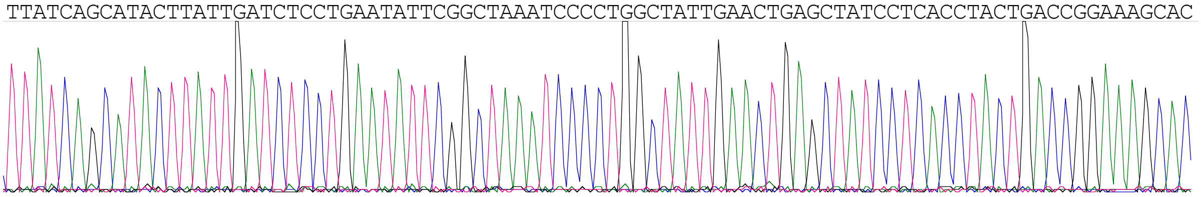

TTATCAGCATACTTATTGATCTCCTGAATATTCGGCTAAATCCCCTGGCTATTGAACTGAGCTATCCTCACCTACTGACCGGAAAGCAC

630 640 650 660 670 680 690 700 710

GGAACCTGATAGAAAAGTAGGCAATGTCAAGTTAAGCCGTAGTTTGGCTACGCGCTAACTAAGGCCCCCCTGACTACACTACTTCTCTT

c3c4(1>1000) → GGAACCTGATAGAAAAGTAGGCAATGTCAAGTTAAGCCGTAGTTTGGCTACGCGCTAACTAAGGCCCCCCTGACTACACTACTTCTCTT

c3c4.ab1(32>550) →

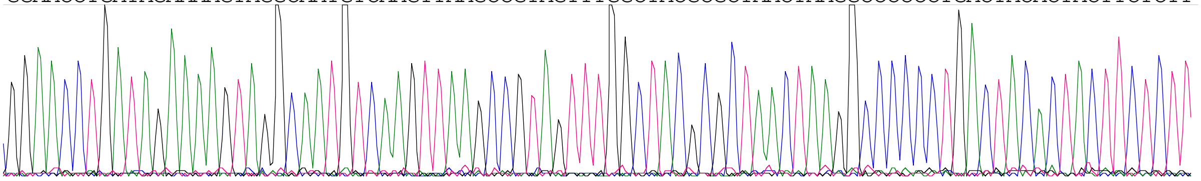

GGAACCTGATAGAAAAGTAGGCAATGTCAAGTTAAGCCGTAGTTTGGCTACGCGCTAACTAAGGCCCCCCTGACTACACTACTTCTCTT

720 730 740 750 760 770 780 790 800

CGGCCAAGCAAGATCGGATATACATCCCCGTTTATAAGGGTATGACATGGAAGTGGTCTAGTCACTACCGAAAGCACCCCCTTCACATG

c3c4(1>1000) → CGGCCAAGCAAGATCGGATATACATCCCCGTTTATAAGGGTATGACATGGAAGTGGTCTAGTCACTACCGAAAGCACCCCCTTCACATG

c3c4.ab1(32>550) →

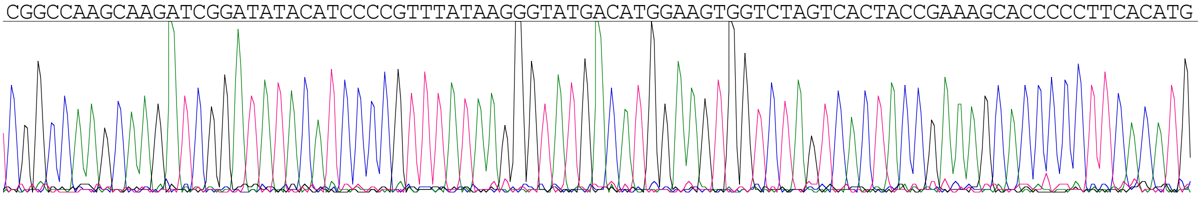

CGGCCAAGCAAGATCGGATATACATCCCCGTTTATAAGGGTATGACATGGAAGTGGTCTAGTCACTACCGAAAGCACCCCCTTCACATG

810 820 830 840 850 860 870 880 890

AAAAAGAGAAAGAGGCGGAGTGACGGGGGGGTATTGCCTTAACTCTCTTTCCGGACCTACTCATCGAAAGATGCCCAAGCTCTTTGATA

c3c4(1>1000) → AAAAAGAGAAAGAGGCGGAGTGACGGGGGGGTATTGCCTTAACTCTCTTTCCGGACCTACTCATCGAAAGATGCCCAAGCTCTTTGATA

c3c4.ab1(32>550) →

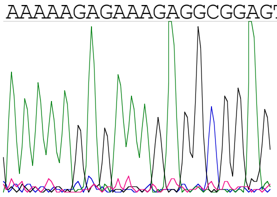

AAAAAGAGAAAGAGGCGGAG

900 910 920 930 940 950 960 970

GAAGAAGCTTCAAATGCGCAGTTAGGACAAAAAAAAAGGCTGTTTGCAAGAATAGGCTCTGGGACTTTATCAAAGGGAATGATTGGACAA

c3c4(1>1000) → GAAGAAGCTTCAAATGCGCAGTTAGGACAAAAAAAAAGGCTGTTTGCAAGAATAGGCTCTGGGACTTTATCAAAGGGAATGATTGGACAA

980 990 1000

AGAGAAGAAGGGCAACTAGTC

c3c4(1>1000) → AGAGAAGAAGGGCAACTAGTC

10 20 30 40 50 60 70 80

GCAAAGGAGACGTACTATAGGAGATAGAGAACTGCTGCCCGTGGTTTTTATCCCTCGCTCCTGAACTGGCGCACTCCGGCAAGAAGGAA

c3c5(1>1000) GCAAAGGAGACGTACTATAGGAGATAGAGAACTGCTGCCCGTGGTTTTTATCCCTCGCTCCTGAACTGGCGCACTCCGGCAAGAAGGAA

90 100 110 120 130 140 150 160 170

CTAACGAGAAGTAGGAAAGGTCACACCACTCTTCTGACTAAAGAAAGGGGCTATTTCAGTTCATCTGTTTGCTCGTTGAAAGAGACTGC

c3c5(1>1000) CTAACGAGAAGTAGGAAAGGTCACACCACTCTTCTGACTAAAGAAAGGGGCTATTTCAGTTCATCTGTTTGCTCGTTGAAAGAGACTGC
c3c5.ab1(40>595)

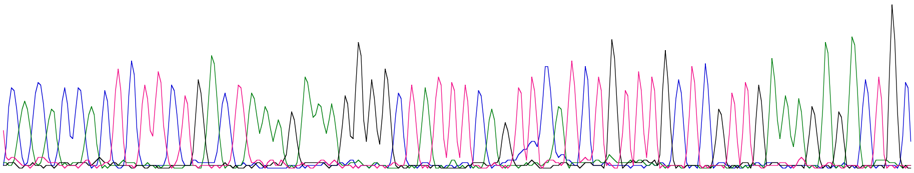

CACACCACTCTTCTGACTAAAGAAAGGGGCTATTTCAGTTCATCTGTTTGCTCGTTGAAAGAGACTGC

180 190 200 210 220 230 240 250 260

TTCAACAAAGCCGTATCTAGCTGGGCCGTAGTTTGCTGATTCGGAGGATAAGTCCTGAGGGGTAGGGACTTTCATCAAGGGTTCTGGGA

c3c5(1>1000) TTCAACAAAGCCGTATCTAGCTGGGCCGTAGTTTGCTGATTCGGAGGATAAGTCCTGAGGGGTAGGGACTTTCATCAAGGGTTCTGGGA
c3c5.ab1(40>595)

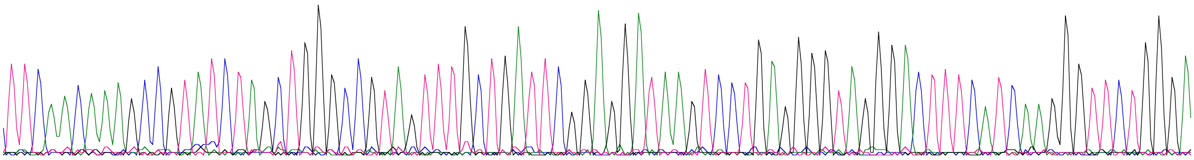

TTCAACAAAGCCGTATCTAGCTGGGCCGTAGTTTGCTGATTCGGAGGATAAGTCCTGAGGGGTAGGGACTTTCATCAAGGGTTCTGGGA

270 280 290 300 310 320 330 340 350

ACGCCCCTATCTATAGGGATAGCTCCCGACATCAATATCTATGGATTAAAGCAACTCCCTCTCGTAGTTGATCCCTTGTTCACAGCTTT

c3c5(1>1000) ACGCCCCTATCTATAGGGATAGCTCCCGACATCAATATCTATGGATTAAAGCAACTCCCTCTCGTAGTTGATCCCTTGTTCACAGCTTT
c3c5.ab1(40>595)

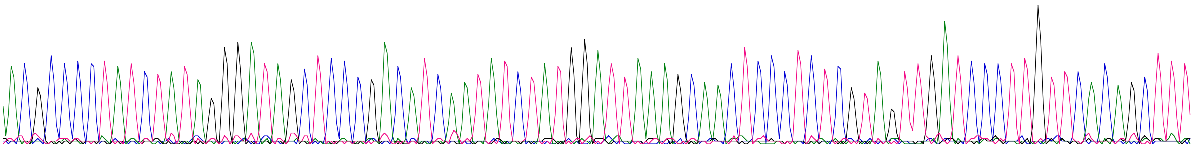

ACGCCCCTATCTATAGGGATAGCTCCCGACATCAATATCTATGGATTAAAGCAACTCCCTCTCGTAGTTGATCCCTTGTTCACAGCTTT

360 370 380 390 400 410 420 430 440

ACTCGGCTCATGCACTCGGCCCGTATGTAAGAGACGGGGACTCCTACTTCCTTGTTAGCAACTTATCCGCTCGGGTCAAAGCCCTTCTC

c3c5(1>1000) ACTCGGCTCATGCACTCGGCCCGTATGTAAGAGACGGGGACTCCTACTTCCTTGTTAGCAACTTATCCGCTCGGGTCAAAGCCCTTCTC
c3c5.ab1(40>595)

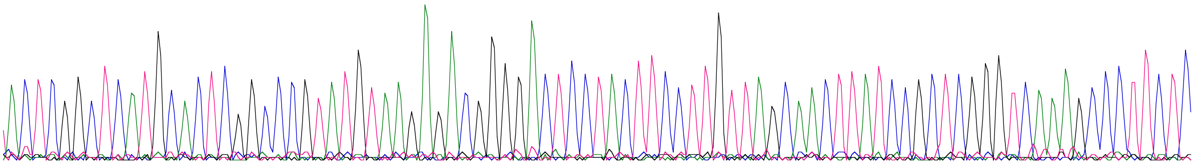

ACTCGGCTCATGCACTCGGCCCGTATGTAAGAGACGGGGACTCCTACTTCCTTGTTAGCAACTTATCCGCTCGGGTCAAAGCCCTTCTC

450 460 470 480 490 500 510 520 530

TTCTTAGTCTGGTGCTTCCTTCATCGAATGAGTATTCATCCGTTGAGCTATCCCTTTCTTAATGTGAAAGCTACGTAAAGAAAGAAGGT

c3c5(1>1000) TTCTTAGTCTGGTGCTTCCTTCATCGAATGAGTATTCATCCGTTGAGCTATCCCTTTCTTAATGTGAAAGCTACGTAAAGAAAGAAGGT
c3c5.ab1(40>595)

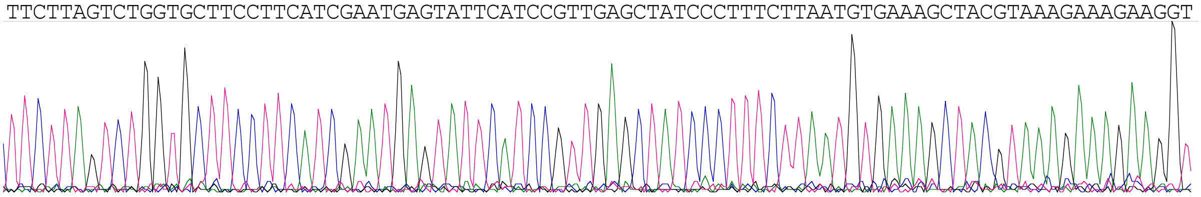

TTCTTAGTCTGGTGCTTCCTTCATCGAATGAGTATTCATCCGTTGAGCTATCCCTTTCTTAATGTGAAAGCTACGTAAAGAAAGAAGGT

540 550 560 570 580 590 600 610 620

GAGATGGGCCTCGTCTTGTTCTATGCCGATGGTTCCCTGGAAGGGGGAGTCTAACTCGCCTTCTATCTCGATGCTGAAATACCTTGAAA

c3c5(1>1000) GAGATGGGCCTCGTCTTGTTCTATGCCGATGGTTCCCTGGAAGGGGGAGTCTAACTCGCCTTCTATCTCGATGCTGAAATACCTTGAAA

c3c5.ab1(40>595)

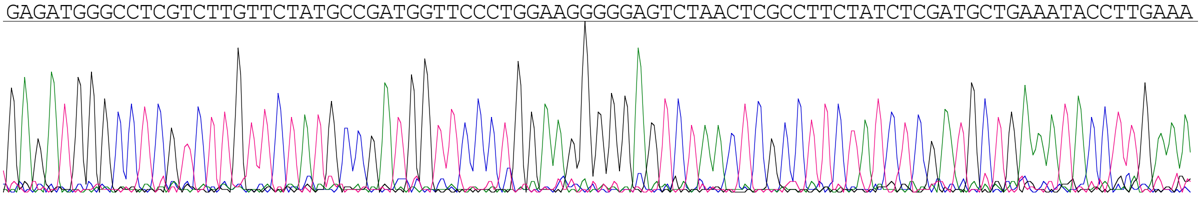

GAGATGGGCCTCGTCTTGTTCTATGCCGATGGTTCCCTGGAAGGGGGAGTCTAACTCGCCTTCTATCTCGATGCTGAAATACCTTGAAA

630 640 650 660 670 680 690 700 710

GAAATGAATTACTTCATCGAGAGCTACGTGAGATGCCGGACCAATATATGTGGATTAGAGCAGCTTCATTAACTGATAAGAGATCCCTT

c3c5(1>1000) GAAATGAATTACTTCATCGAGAGCTACGTGAGATGCCGGACCAATATATGTGGATTAGAGCAGCTTCATTAACTGATAAGAGATCCCTT

c3c5.ab1(40>595)

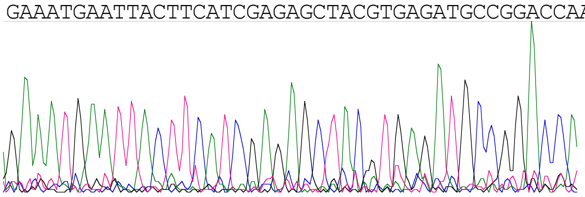

GAAATGAATTACTTCATCGAGAGCTACGTGAGATGCCGGACCA

720 730 740 750 760 770 780 790 800

TTGACAGAAGAATGAAGCTCACGCAACAAATGCCTAGGATCAGGATGTGAACGATAGGGATCGTCATTCTATCCTTGAATGAGCCTTTG

c3c5(1>1000) TTGACAGAAGAATGAAGCTCACGCAACAAATGCCTAGGATCAGGATGTGAACGATAGGGATCGTCATTCTATCCTTGAATGAGCCTTTG

810 820 830 840 850 860 870 880 890

CTAGCCAACGAGAGCGGAAGAAAGAGCATTTGCCTTTCTTTCCAGGCATTGATTTCGAAGAACATCTTAGAGTAAGTTTTTGGTGCCCG

c3c5(1>1000) CTAGCCAACGAGAGCGGAAGAAAGAGCATTTGCCTTTCTTTCCAGGCATTGATTTCGAAGAACATCTTAGAGTAAGTTTTTGGTGCCCG

900 910 920 930 940 950 960 970

ATCTTGATCGACTACTTTGTCTCGAAGAAATACGGGTGACTTTCTCAAAAGCTCTTACCGCTCCGTGGGCCTAGGCTACACGGAAGAGC

c3c5(1>1000) ATCTTGATCGACTACTTTGTCTCGAAGAAATACGGGTGACTTTCTCAAAAGCTCTTACCGCTCCGTGGGCCTAGGCTACACGGAAGAGC

980 990 1000

ATACGATTTACAGCTTTTAGC

c3c5(1>1000) ATACGATTTACAGCTTTTAGC

10 20 30 40 50 60 70 80

GCCCTACCTAAACCAATCATCATATCGGTCCCTAGGCCCCATTGCTGGAAAGGCTCGGCTTCAAAACCGTACGTGGAGCTTCCGCCTCA

c4c7(1>1000) → GCCCTACCTAAACCAATCATCATATCGGTCCCTAGGCCCCATTGCTGGAAAGGCTCGGCTTCAAAACCGTACGTGGAGCTTCCGCCTCA

90 100 110 120 130 140 150 160 170

TACGGCTCCTCTAGGGATGGAGGTAGGCCCAGCCCAGGCTTGTGCGGTTAAGGTTGTTGTACACGACCTCATAAGAAAAGAATGAAAAA

c4c7(1>1000) → TACGGCTCCTCTAGGGATGGAGGTAGGCCCAGCCCAGGCTTGTGCGGTTAAGGTTGTTGTACACGACCTCATAAGAAAAGAATGAAAAA

180 190 200 210 220 230 240 250 260

AACGATGTTTCCATCTTTCAGAGCGGACCCTTCCTCATAGTTTTCACTAATCGGGGAAGTGATCCTTAATGAGGGATTTCAGGAGAGCT

c4c7(1>1000) → AACGATGTTTCCATCTTTCAGAGCGGACCCTTCCTCATAGTTTTCACTAATCGGGGAAGTGATCCTTAATGAGGGATTTCAGGAGAGCT
c4c7.ab1(37>474) →

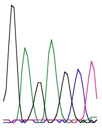

GAGAGCT

270 280 290 300 310 320 330 340 350

GGGTCTTTGACGGCCTCGTTCCAGCTTCGGAGGGTGTCTGCATCCATCTCCGAAGAGATCTCCAAATCCCCCATCAAGTTCCTTGCCCC

c4c7(1>1000) → GGGTCTTTGACGGCCTCGTTCCAGCTTCGGAGGGTGTCTGCATCCATCTCCGAAGAGATCTCCAAATCCCCCATCAAGTTCCTTGCCCC
c4c7.ab1(37>474) →

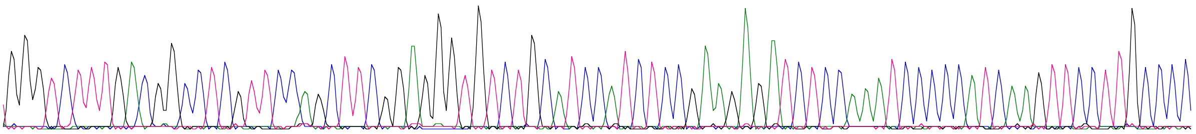

GGGTCTTTGACGGCCTCGTTCCAGCTTCGGAGGGTGTCTGCATCCATCTCCGAAGAGATCTCCAAATCCCCCATCAAGTTCCTTGCCCC

360 370 380 390 400 410 420 430 440

CTGGGCGTAGATTTCGTCTTGCTCGGGAAAGCGAGACATCCCCCTCCGCCACGGTTGACGTTCACAACGGTGCCGAATAAGTTCACCTA

c4c7(1>1000) → CTGGGCGTAGATTTCGTCTTGCTCGGGAAAGCGAGACATCCCCCTCCGCCACGGTTGACGTTCACAACGGTGCCGAATAAGTTCACCTA
c4c7.ab1(37>474) →

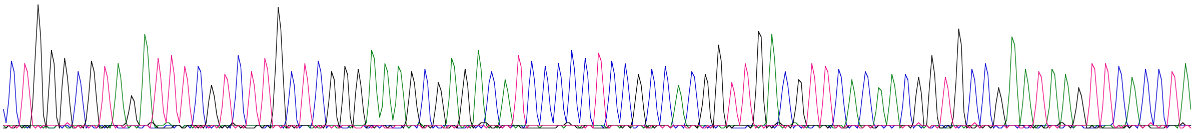

CTGGGCGTAGATTTCGTCTTGCTCGGGAAAGCGAGACATCCCCCTCCGCCACGGTTGACGTTCACAACGGTGCCGAATAAGTTCACCTA

450 460 470 480 490 500 510 520 530

TTTGAGCGCGTATTGACCGGAGAAGTTCCTCCCCTTGGTGAACTTCCGCCGGAGCCTCGCCCTTTTTTCTCATTATCGAAGGAGCAGGA

c4c7(1>1000) → TTTGAGCGCGTATTGACCGGAGAAGTTCCTCCCCTTGGTGAACTTCCGCCGGAGCCTCGCCCTTTTTTCTCATTATCGAAGGAGCAGGA
c4c7.ab1(37>474) →

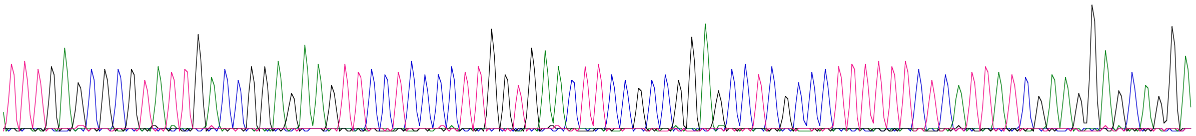

TTTGAGCGCGTATTGACCGGAGAAGTTCCTCCCCTTGGTGAACTTCCGCCGGAGCCTCGCCCTTTTTTCTCATTATCGAAGGAGCAGGA

540 550 560 570 580 590 600 610 620

AGAGCTCAAAAAAACCGTTAAACGCCTTCGCTATTATAAAGGGATGCTCAATTATTGGGAGGGGGAAATGGATAAGTGTATTCAGAAAG

c4c7(1>1000) → AGAGCTCAAAAAAACCGTTAAACGCCTTCGCTATTATAAAGGGATGCTCAATTATTGGGAGGGGGAAATGGATAAGTGTATTCAGAAAG
c4c7.ab1(37>474) →

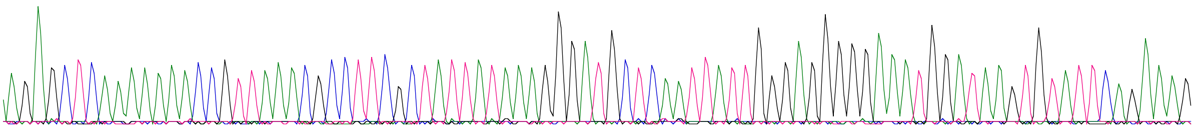

AGAGCTCAAAAAAACCGTTAAACGCCTTCGCTATTATAAAGGGATGCTCAATTATTGGGAGGGGGAAATGGATAAGTGTATTCAGAAAG

```
          630       640       650       660       670       680       690       700       710
ACATGGAGAGGCGCGAAAAGAGGCGGAGGGGCGGAGGAGGGCTCTAAGCGGATTAAAAAAGTCGATCTATACGGTCGACCCCGCTCGGC
c4c7(1>1000)       → ACATGGAGAGGCGCGAAAAGAGGCGGAGGGGCGGAGGAGGGCTCTAAGCGGATTAAAAAAGTCGATCTATACGGTCGACCCCGCTCGGC
c4c7.ab1(37>474)   →
```

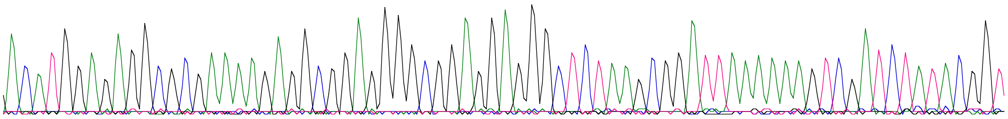

```
ACATGGAGAGGCGCGAAAAGAGGCGGAGGGGCGGAGGAGGGCTCTAAGCGGATTAAAAAAGTCGATCTATACGGT

           720       730       740       750       760       770       780       790       800
TGGCATCCGATTATGCCCCCGGACGTATGCCCTTTAATTTAGTCATTCATTGAAGAGGAATCATTCCATTTTGAATGGAAGCTGTGAAA
c4c7(1>1000)       → TGGCATCCGATTATGCCCCCGGACGTATGCCCTTTAATTTAGTCATTCATTGAAGAGGAATCATTCCATTTTGAATGGAAGCTGTGAAA

            810       820       830       840       850       860       870       880       890
CCAATCCCGATGAAGCTCTTTTTTATTTTTTTAGTGAAAAAAAAAGATTGCTGCGTCCTATAGGAACTGCCGGGATTTCCACATGAGAG
c4c7(1>1000)       → CCAATCCCGATGAAGCTCTTTTTTATTTTTTTAGTGAAAAAAAAAGATTGCTGCGTCCTATAGGAACTGCCGGGATTTCCACATGAGAG

             900       910       920       930       940       950       960       970
AAAAAATGGAAATGGAACAATTGAACACCATGATCCAAGATCTGAAACAGAAAAGATCCGCGTCCTCGACCTTTCATTCAATTGTCCAG
c4c7(1>1000)       → AAAAAATGGAAATGGAACAATTGAACACCATGATCCAAGATCTGAAACAGAAAAGATCCGCGTCCTCGACCTTTCATTCAATTGTCCAG

980       990       1000
GCTTGTTTCCCTCCCGATACT
c4c7(1>1000)       → GCTTGTTTCCCTCCCGATACT
```

10 20 30 40 50 60 70 80

TAGTGAGTTAGGCCGGCAAACACCAAAACCTCATGGCCTTTGCCAGATAAATTACTAACATTCCCCAGGTGCCAATCACTAAAGAGGAA

c5c7(1>1000) → TAGTGAGTTAGGCCGGCAAACACCAAAACCTCATGGCCTTTGCCAGATAAATTACTAACATTCCCCAGGTGCCAATCACTAAAGAGGAA

90 100 110 120 130 140 150 160 170

TACCCCGAAAAGGGGATGTTTTCATGAACAAATCTCTCCAGATCTTGCCCACACCGCTGAACGCTCCGCTCATACGGATAAAGCCCACT

c5c7(1>1000) → TACCCCGAAAAGGGGATGTTTTCATGAACAAATCTCTCCAGATCTTGCCCACACCGCTGAACGCTCCGCTCATACGGATAAAGCCCACT

180 190 200 210 220 230 240 250 260

CAAAGCCGCCCCTAACCGAGTTTGTGGGAGAAAAAAAGACCCGGACGAAGCGCTTCGCCTTTAAGCCCTAAAAAGAAAGGTGCTACACT

c5c7(1>1000) → CAAAGCCGCCCCTAACCGAGTTTGTGGGAGAAAAAAAGACCCGGACGAAGCGCTTCGCCTTTAAGCCCTAAAAAGAAAGGTGCTACACT
c5c7.ab1(38>552) →

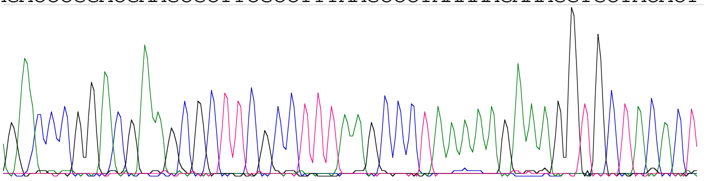

GACCCGGACGAAGCGCTTCGCCTTTAAGCCCTAAAAAGAAAGGTGCTACACT

270 280 290 300 310 320 330 340 350

AATTGTAGTAACCAGAACAGGGCCTTGGCCCCAGATGCTATGGAACACCTTTCCTTCCTTAGCACAAGCGCTAAGCTATAATAATTCCT

c5c7(1>1000) → AATTGTAGTAACCAGAACAGGGCCTTGGCCCCAGATGCTATGGAACACCTTTCCTTCCTTAGCACAAGCGCTAAGCTATAATAATTCCT
c5c7.ab1(38>552) →

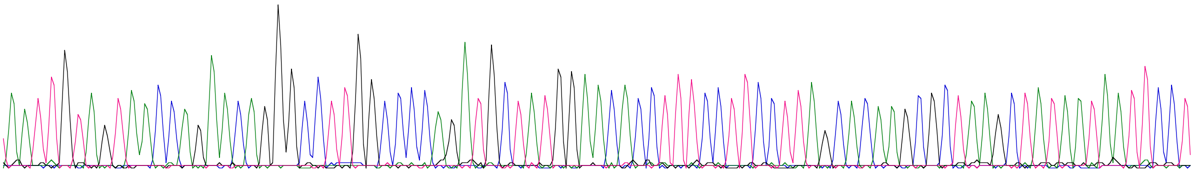

AATTGTAGTAACCAGAACAGGGCCTTGGCCCCAGATGCTATGGAACACCTTTCCTTCCTTAGCACAAGCGCTAAGCTATAATAATTCCT

360 370 380 390 400 410 420 430 440

TGCCAACCAGAATTCATTCACCTTCTTTTTCAGGCAGGAGAAGGGCGGCTTCATCCTCATCAGATGCAGCGGACCGAGCGATCGAAGTT

c5c7(1>1000) → TGCCAACCAGAATTCATTCACCTTCTTTTTCAGGCAGGAGAAGGGCGGCTTCATCCTCATCAGATGCAGCGGACCGAGCGATCGAAGTT
c5c7.ab1(38>552) →

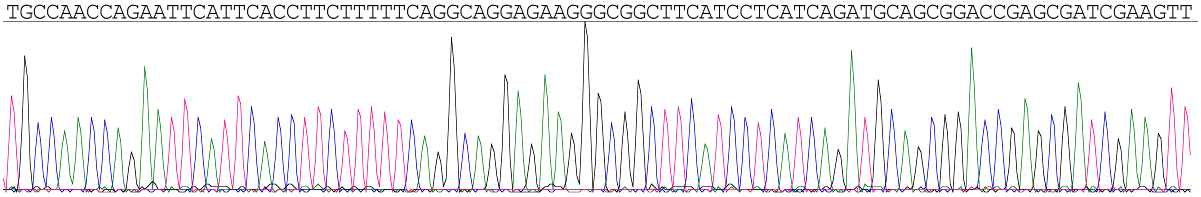

TGCCAACCAGAATTCATTCACCTTCTTTTTCAGGCAGGAGAAGGGCGGCTTCATCCTCATCAGATGCAGCGGACCGAGCGATCGAAGTT

450 460 470 480 490 500 510 520 530

CAGTGAAGTAGGCATTACCTTTGACACACACATGGGTGCAATAGGAAAGGGAGCAAGTCCGCACCGGTCTTGTGTTACCTCACTAGCTC

c5c7(1>1000) → CAGTGAAGTAGGCATTACCTTTGACACACACATGGGTGCAATAGGAAAGGGAGCAAGTCCGCACCGGTCTTGTGTTACCTCACTAGCTC
c5c7.ab1(38>552) →

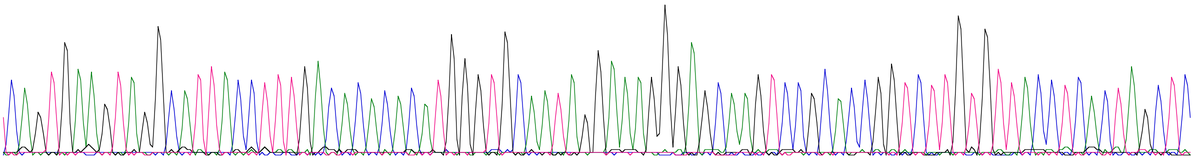

CAGTGAAGTAGGCATTACCTTTGACACACACATGGGTGCAATAGGAAAGGGAGCAAGTCCGCACCGGTCTTGTGTTACCTCACTAGCTC

540 550 560 570 580 590 600 610 620

ACCGACCCCGGCATCGCTACGTTCGTTCACTCAAAGAAAACTTCCCCCTTACTGGAGGGGAACTTTCTCATAAGTAATGGGCGCAGTCC

c5c7(1>1000) → ACCGACCCCGGCATCGCTACGTTCGTTCACTCAAAGAAAACTTCCCCCTTACTGGAGGGGAACTTTCTCATAAGTAATGGGCGCAGTCC
c5c7.ab1(38>552) →

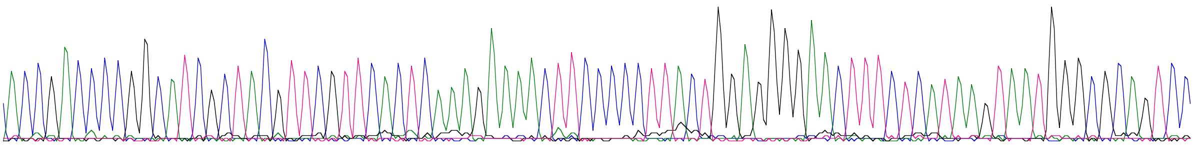

ACCGACCCCGGCATCGCTACGTTCGTTCACTCAAAGAAAACTTCCCCCTTACTGGAGGGGAACTTTCTCATAAGTAATGGGCGCAGTCC

630 640 650 660 670 680 690 700 710

ATATAGGAGACTCGACCAGATCTCTTACCCATTCTCATTTCAAGAGGAAGGCATCCTTGGTTAAGGTATAGACCGAAACCGGTCCAACT

c5c7(1>1000) → ATATAGGAGACTCGACCAGATCTCTTACCCATTCTCATTTCAAGAGGAAGGCATCCTTGGTTAAGGTATAGACCGAAACCGGTCCAACT

c5c7.ab1(38>552) →

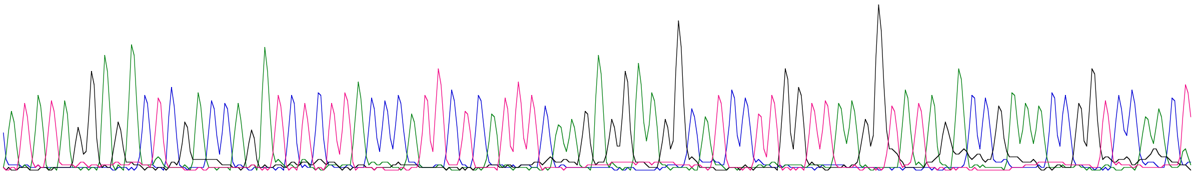

ATATAGGAGACTCGACCAGATCTCTTACCCATTCTCATTTCAAGAGGAAGGCATCCTTGGTTAAGGTATAGACCGAAACCGGTCCAACT

720 730 740 750 760 770 780 790 800

GGTCAAGAAAGCAACCGTCCATTTGCCCTCATTTCACAGGTTGAGCGAAGCTTAGGACAGCATTCAGCTTTCATATGCATTTCTTACAG

c5c7(1>1000) → GGTCAAGAAAGCAACCGTCCATTTGCCCTCATTTCACAGGTTGAGCGAAGCTTAGGACAGCATTCAGCTTTCATATGCATTTCTTACAG

c5c7.ab1(38>552) →

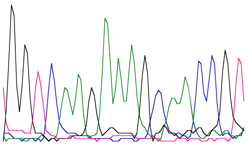

GGTCAAGAAAGCAACCGT

810 820 830 840 850 860 870 880 890

ATCGGGATTCTTCGATTACAGCATAGCTATCATCAGAAGCAGTACTTCCGAGTCACTCTATAACTGAGAGCTTGTAGTTGGGGATTTAC

c5c7(1>1000) → ATCGGGATTCTTCGATTACAGCATAGCTATCATCAGAAGCAGTACTTCCGAGTCACTCTATAACTGAGAGCTTGTAGTTGGGGATTTAC

900 910 920 930 940 950 960 970

CAGATCTCTAAGATAGAGTCTATCCGACGTATCACCCGTAATTGGGAGTTCAAACGAGGCCTCAACGTAGATGCTGGATGGGAACTTCT

c5c7(1>1000) → CAGATCTCTAAGATAGAGTCTATCCGACGTATCACCCGTAATTGGGAGTTCAAACGAGGCCTCAACGTAGATGCTGGATGGGAACTTCT

980 990 1000

ATTCTACCTGGCTTATTTATA

c5c7(1>1000) → ATTCTACCTGGCTTATTTATA

10 20 30 40 50 60 70 80

GCCCTACCTAAACCAATCATCATATCGGTCCCTAGGCCCCATTGCTGGAAAGGCTCGGCTTCAAAACCGTACGTGGAGCTTCCGCCTCA

c6c7(1>1000) → GCCCTACCTAAACCAATCATCATATCGGTCCCTAGGCCCCATTGCTGGAAAGGCTCGGCTTCAAAACCGTACGTGGAGCTTCCGCCTCA

90 100 110 120 130 140 150 160 170

TACGGCTCCTCTAGGGATGGAGGTAGGCCCAGCCCAGGCTTGTGCGGTTAAGGTTGTTGTACACGACCTCATAAGAAAAGAATGAAAAA

c6c7(1>1000) → TACGGCTCCTCTAGGGATGGAGGTAGGCCCAGCCCAGGCTTGTGCGGTTAAGGTTGTTGTACACGACCTCATAAGAAAAGAATGAAAAA

180 190 200 210 220 230 240 250 260

AACGATGTTTCCATCTTTCAGAGCGGACCCTTCCTCATAGTTTTCACTAATCGGGGAAGTGATCCTTAATGAGGGATTTCAGGAGAGCT

c6c7(1>1000) → AACGATGTTTCCATCTTTCAGAGCGGACCCTTCCTCATAGTTTTCACTAATCGGGGAAGTGATCCTTAATGAGGGATTTCAGGAGAGCT
c6c7.ab1(35>572) →

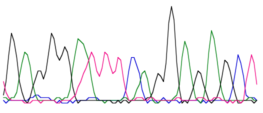

270 280 290 300 310 320 330 340 350

GGGTCTTTGACGGCCTCGTTCCAGCTTCGGAGGGTGTCTGCATCCATCTCCGAAGAGATCTCCAAATCCCCCATCAAGTTCCTTGCCCC

c6c7(1>1000) → GGGTCTTTGACGGCCTCGTTCCAGCTTCGGAGGGTGTCTGCATCCATCTCCGAAGAGATCTCCAAATCCCCCATCAAGTTCCTTGCCCC
c6c7.ab1(35>572) →

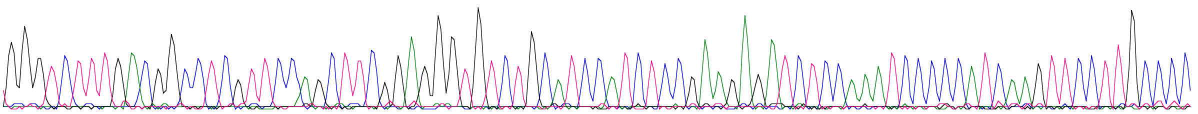

360 370 380 390 400 410 420 430 440

CTGGGCGTAGATTTCGTCTTGCTCGGGAAAGCGAGACATCCCCCTCCGCCACGGTTGACGTTCACAACGGTGCCGAATAAGTTCACCTA

c6c7(1>1000) → CTGGGCGTAGATTTCGTCTTGCTCGGGAAAGCGAGACATCCCCCTCCGCCACGGTTGACGTTCACAACGGTGCCGAATAAGTTCACCTA
c6c7.ab1(35>572) →

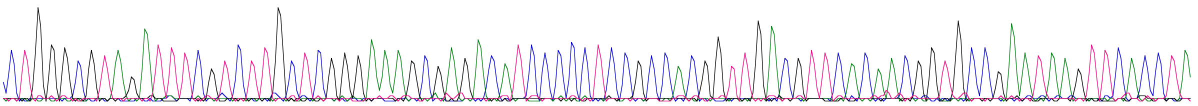

450 460 470 480 490 500 510 520 530

TTTGAGCGCGTATTGACCGGAGAAGTTCCTCCCCTTGGTGAACTTCCGCCGGAGCATTCTCCGGCGCAGGCTCAGCAGGAGGGGCCGGC

c6c7(1>1000) → TTTGAGCGCGTATTGACCGGAGAAGTTCCTCCCCTTGGTGAACTTCCGCCGGAGCATTCTCCGGCGCAGGCTCAGCAGGAGGGGCCGGC
c6c7.ab1(35>572) →

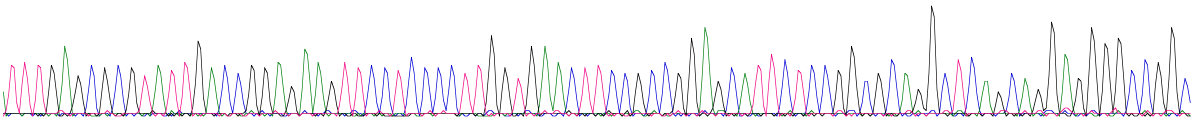

540 550 560 570 580 590 600 610 620

GCAGGCTCAGCAGGAGGGGCCGGCTCAGGCTCAGCAGGAGGGGGGCCGTTGAGATCTAGCCCCGGCTCAGGAGCTGGAGGTTGGTTTAG

c6c7(1>1000) → GCAGGCTCAGCAGGAGGGGCCGGCTCAGGCTCAGCAGGAGGGGGGCCGTTGAGATCTAGCCCCGGCTCAGGAGCTGGAGGTTGGTTTAG
c6c7.ab1(35>572) →

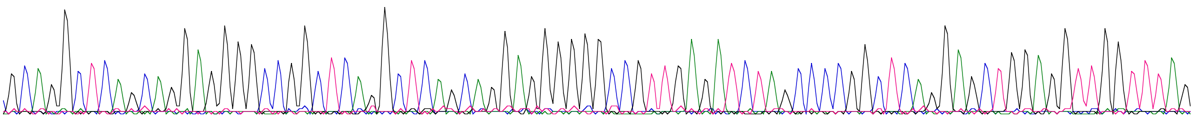

630 640 650 660 670 680 690 700 710

ATCGAGCTCCGGGCGCCCCGAACTCGAAGGCCCGGCATCGCTTCCGCCGCCTCCAACGGAAAGAGGGAGTCTGTCCATGGAAGACAGAA

c6c7(1>1000) ATCGAGCTCCGGGCGCCCCGAACTCGAAGGCCCGGCATCGCTTCCGCCGCCTCCAACGGAAAGAGGGAGTCTGTCCATGGAAGACAGAA

c6c7.ab1(35>572)

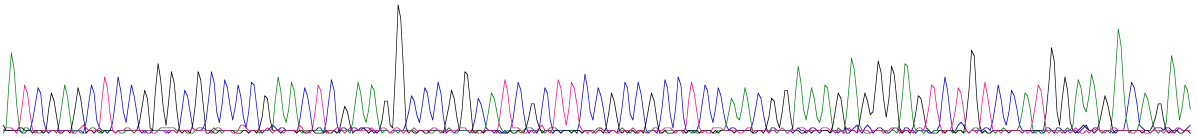

ATCGAGCTCCGGGCGCCCCGAACTCGAAGGCCCGGCATCGCTTCCGCCGCCTCCAACGGAAAGAGGGAGTCTGTCCATGGAAGACAGAA

720 730 740 750 760 770 780 790 800

TCCAATCGATCCAGTCCACAGAGAAAACAAAGAAAGGCCAAGTAAAGCCCCATGCAAGCATCAGGAACGAAATCAAGAAGAAAATAAAG

c6c7(1>1000) TCCAATCGATCCAGTCCACAGAGAAAACAAAGAAAGGCCAAGTAAAGCCCCATGCAAGCATCAGGAACGAAATCAAGAAGAAAATAAAG

c6c7.ab1(35>572)

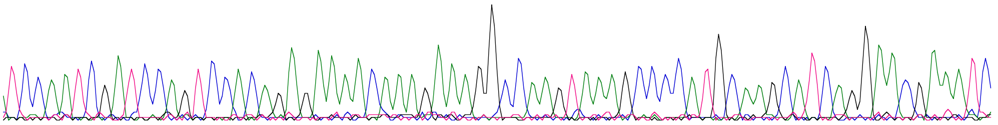

TCCAATCGATCCAGTCCACAGAGAAAACAAAGAAAGGCCAAGTAAAGCCCCATGCAAGCATCAGGAACGAAATC

810 820 830 840 850 860 870 880 890

TGAATAAATTATTATTAGAATCGCTTTTCTTTCCATAGCGCAAGTCCTTCCAGTATCTATAGAATGAGCACTGTAAACTATCTGCTTCA

c6c7(1>1000) TGAATAAATTATTATTAGAATCGCTTTTCTTTCCATAGCGCAAGTCCTTCCAGTATCTATAGAATGAGCACTGTAAACTATCTGCTTCA

900 910 920 930 940 950 960 970

AAAAGAGAGTTGTAAGAATGAAACTTCTTCGATCGGAATACGGATGAGATCAAAAAGGCCATCATTGGGGCAAACGATGCAATAGCTTC

c6c7(1>1000) AAAAGAGAGTTGTAAGAATGAAACTTCTTCGATCGGAATACGGATGAGATCAAAAAGGCCATCATTGGGGCAAACGATGCAATAGCTTC

980 990 1000

GGTTAGAGCAAAGCCCATATT

c6c7(1>1000) GGTTAGAGCAAAGCCCATATT

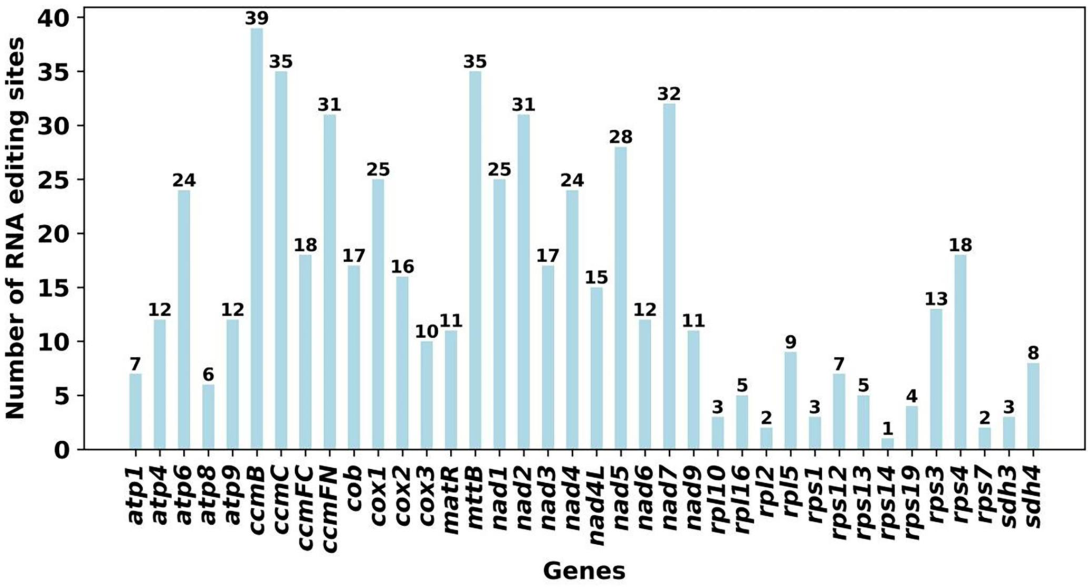

Figure S7. Numbers of predicted RNA editing sites in mitochondria of *Camellia oleifera* and *Camellia lanceoleosa.*
